# Maximize Resolution or Minimize Error? Using Genotyping-By-Sequencing to Investigate the Recent Diversification of *Helianthemum* (Cistaceae)

**DOI:** 10.3389/fpls.2019.01416

**Published:** 2019-11-11

**Authors:** Sara Martín-Hernanz, Abelardo Aparicio, Mario Fernández-Mazuecos, Encarnación Rubio, J. Alfredo Reyes-Betancort, Arnoldo Santos-Guerra, María Olangua-Corral, Rafael G. Albaladejo

**Affiliations:** ^1^Departamento de Biología Vegetal y Ecología, Universidad de Sevilla, Sevilla, Spain; ^2^Departamento de Biodiversidad y Conservación, Real Jardín Botánico (RJB-CSIC), Madrid, Spain; ^3^Jardín de Aclimatación de la Orotava, Instituto Canario de Investigaciones Agrarias (ICIA), Santa Cruz de Tenerife, Spain; ^4^Departamento de Biología Reproductiva y Micro-morfología, Jardín Botánico Canario ‘Viera y Clavijo’—Unidad Asociada CSIC (Cabildo de Gran Canaria), Las Palmas de Gran Canaria, Spain

**Keywords:** branch length, diversification, evolutionary radiation, genotyping-by-sequencing, *Helianthemum*, phylogenetic resolution, phylogenomics

## Abstract

A robust phylogenetic framework, in terms of extensive geographical and taxonomic sampling, well-resolved species relationships and high certainty of tree topologies and branch length estimations, is critical in the study of macroevolutionary patterns. Whereas Sanger sequencing-based methods usually recover insufficient phylogenetic signal, especially in recently diversified lineages, reduced-representation sequencing methods tend to provide well-supported phylogenetic relationships, but usually entail remarkable bioinformatic challenges due to the inherent trade-off between the number of SNPs and the magnitude of associated error rates. The genus *Helianthemum* (Cistaceae) is a species-rich and taxonomically complex Palearctic group of plants that diversified mainly since the Upper Miocene. It is a challenging case study since previous attempts using Sanger sequencing were unable to resolve the intrageneric phylogenetic relationships. Aiming to obtain a robust phylogenetic reconstruction based on genotyping-by-sequencing (GBS), we established a rigorous methodological workflow in which we i) explored how variable settings during dataset assembly have an impact on error rates and on the degree of resolution under concatenation and coalescent approaches, ii) assessed the effect of two extreme parameter configurations (minimizing error rates vs. maximizing phylogenetic resolution) on tree topology and branch lengths, and iii) evaluated the effects of these two configurations on estimates of divergence times and diversification rates. Our analyses produced highly supported topologically congruent phylogenetic trees for both configurations. However, minimizing error rates did produce more reliable branch lengths, critically affecting the accuracy of downstream analyses (i.e. divergence times and diversification rates). In addition to recommending a revision of intrageneric systematics, our results enabled us to identify three highly diversified lineages in *Helianthemum* in contrasting geographical areas and ecological conditions, which started radiating in the Upper Miocene.

## Introduction

The establishment of a robust phylogenetic framework is the initial step for the study of macroevolutionary patterns of specific lineages and requires extensive geographical and taxonomic representativeness, strong statistical support for species relationships and accurate estimates of tree topology and branch lengths. Usually, these goals cannot be achieved in phylogenetic analyses of recently diversified lineages when Sanger sequencing approaches are used. Such techniques typically rely on a small set of relatively slowly evolving loci, which frequently provide insufficient synapomorphies for resolving species relationships. Furthermore, with a small number of loci it is difficult to deal with inconsistencies related to incomplete lineage sorting (ILS; DeFilippis and Moore, 2000; Whitfield and Kjer, 2008) and inter-specific gene flow (Shaw, 2002). As a result, poor resolution and low statistical support are often obtained (DeFilippis and Moore, 2000).

Alternatively, reduced-representation sequencing methods such as restriction-site associated DNA sequencing (RADseq; Miller et al., 2007; Baird et al., 2008; Rowe et al., 2011) and genotyping-by-sequencing (GBS; Elshire et al., 2011) have been shown to be highly efficient in phylogenetic reconstructions of recently diversified lineages given that they allow for the discovery of thousands of genetic markers in non-model species (e.g. Nadeau et al., 2013; Wagner et al., 2013; Fernández-Mazuecos et al., 2018). However, these methods based on Next-Generation Sequencing (NGS) present notable methodological challenges that include i) the high DNA quality generally required (Andrews et al., 2016), ii) the complexity of the assembly and bioinformatic processing (Shafer et al., 2017), iii) the constraints and assumptions of the two approaches currently used in phylogenomics (i.e. concatenation and coalescent approaches; Meiklejohn et al., 2016), iv) the limits of available computing power (Glor, 2010), and v) the biological limitations on data collection (i.e. allele dropout because of mutations at restriction sites; Andrews et al., 2016; **Table S1**).

The assembly and bioinformatic processing of data derived from reduced-representation sequencing methods require many steps and decisions to convert data into a format ready for analysis, which can entail a trade-off between the numbers of loci and SNPs (single-nucleotide polymorphisms) recovered and the magnitude of associated error rates, especially when studying recently diversified lineages (Mastretta-Yanes et al., 2015; Anderson et al., 2017; Lee et al., 2018). Non-optimized values of key assembly parameters such as the clustering threshold, minimum sample coverage and minimum taxon coverage may lead to errors in genotyping and large amounts of missing data (Mastretta-Yanes et al., 2015; Anderson et al., 2017; see **Table S1**), which, in turn, may have an unpredictable impact on phylogenetic inferences in terms of degree of resolution, topology, and branch length estimation (Lemmon et al., 2009; Roure et al., 2013; Mastretta-Yanes et al., 2015; Darriba et al., 2016; Anderson et al., 2017). Furthermore, concatenation and coalescent approaches, frequently used in phylogenomics, are also prone to a number of sources of error that need to be taken into account when reduced-representation sequencing data are used. The concatenation approach, in which all gene alignments are concatenated into a single matrix assuming that all trees share the same history (e.g. Nadeau et al., 2013; Wagner et al., 2013; Cruaud et al., 2014), has been shown to be robust for phylogenetic inference from reduced-representation sequencing data by certain simulations (Rivers et al., 2016). However, other studies indicate that the resulting trees can be misleading in terms of species relationships and tree support (e.g. strong bootstrap support for incorrect relationships) (Kubatko and Degnan, 2007; McVay and Carstens, 2013; **Table S1**) and that this approach is unable to address the problem of ILS (Kubatko and Degnan, 2007). Conversely, the coalescent approach is capable of dealing with ILS and can also be used for constructing species trees in large-scale phylogenomic studies. Within this approach, there are several families of methods, including "summary methods," in which all genes are analysed separately and the resulting gene tree topologies are subsequently or simultaneously used to construct a species tree based on coalescent theory (Liu and Yu, 2011); and "site-based methods," which do not try to estimate gene trees but estimate the species tree directly from the observed site pattern frequencies using properties of the multispecies coalescent model (Chifman and Kubatko, 2014; Vachaspati and Warnow, 2018). Nonetheless, summary methods are sensitive to errors in gene tree estimation (Dupuis et al., 2017) due to insufficient variable sites per locus, and both families of methods may be computationally intensive (reviewed by Liu et al., 2015; Solís-Lemus and Ané, 2016). In general, the limits of available computing power have led researchers to focus on estimating phylogenies of small clades when using reduced-representation sequencing methods (e.g. Jones et al., 2013, Nadeau et al., 2013, Anderson et al., 2017). Taxon-rich clades have been addressed less frequently, even though sampling more taxa affords a wider comparative framework needed for downstream analyses of evolutionary patterns (e.g. divergence time estimates, diversification rate calculations; Hughes et al., 2015; Eaton et al., 2017).

Despite being a challenging case from both systematic and evolutionary standpoints, the genus *Helianthemum* Mill. (Cistaceae) is suitable for testing the trade-off between phylogenetic information and error rates under the two described phylogenomic approaches. *Helianthemum* is by far the largest genus in the Cistaceae, constituting a monophyletic, complex and species-rich Palearctic plant clade with *c.* 140 taxa (104 species and 36 subspecies). Its diversification has probably been driven by the major palaeoclimatic events that have affected the Mediterranean Basin since the Upper Miocene (i.e. the Messinian salinity crisis, the infilling of the Mediterranean Basin and the climatic cycles during the Pleistocene; Aparicio et al., 2017). Despite high geographical and taxonomical representativeness, a previous attempt to infer phylogenetic relationships in *Helianthemum* based on Sanger sequencing of combined ITS and cpDNA sequences (Aparicio et al., 2017) resulted in very low resolution and low statistical support for shallow nodes. However, support was recovered for three main clades with intriguing systematic and evolutionary patterns. In particular, the internal topologies of these three clades were similar, each including a species-rich subclade (corresponding with the three largest taxonomical sects. *Eriocarpum*, *Pseudocistus*, and *Helianthemum*) sister to poorly diversified subclades, an asymmetry that can be an indicator of recent and rapid radiations (Nee et al., 1996; Sanderson and Donoghue, 1996; Pybus and Harvey, 2000).

**Figure 1:**
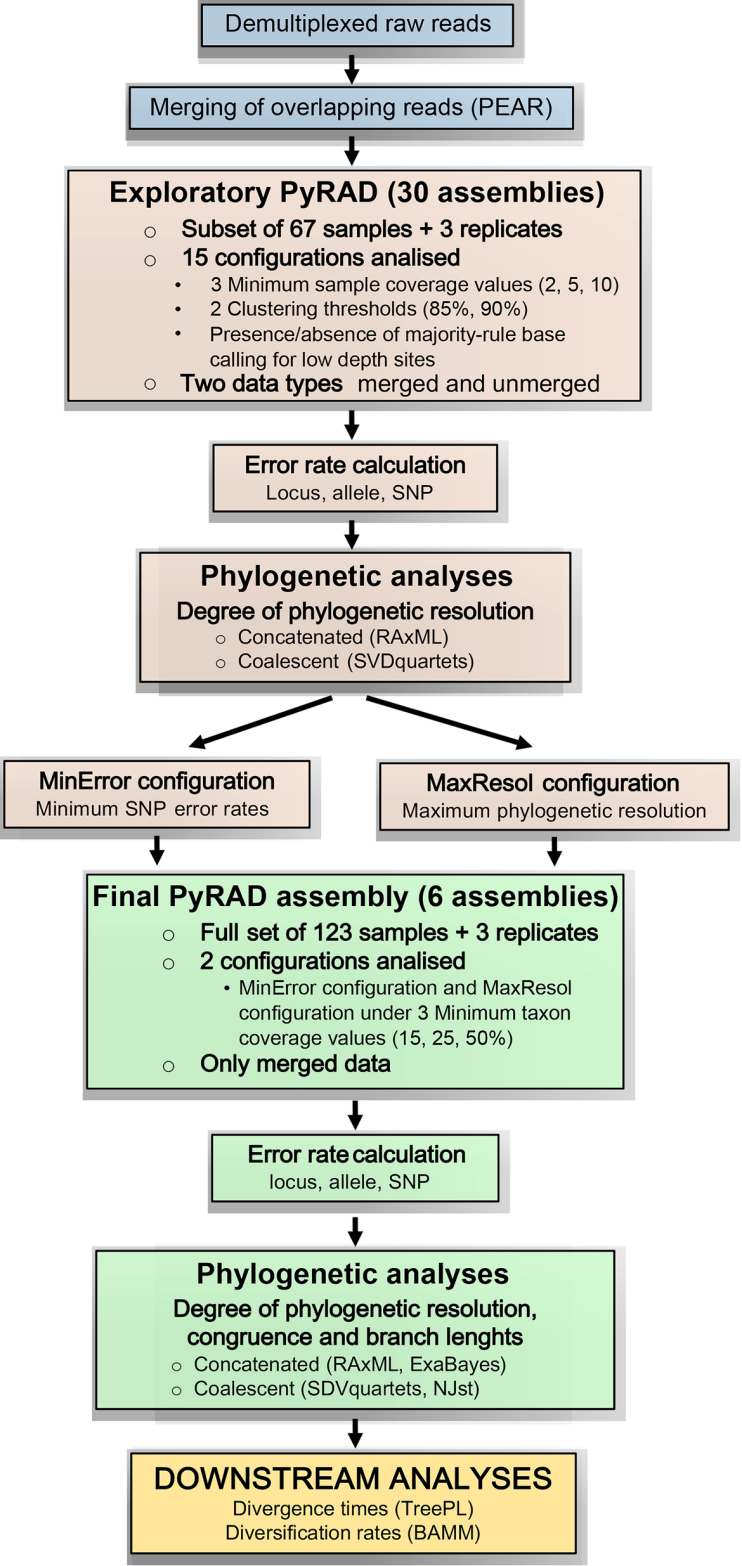
Bioinformatic and analytical workflow used to process genotyping-by-sequencing (GBS) data for the genus *Helianthemum* (modified from Anderson et al., 2017). Blue rectangles represent the pre-processing of raw reads applied to all studied samples; brown rectangles represent the exploratory analyses applied to a subset of the studied samples; green rectangles represent the final analyses applied to the full set of studied samples; and the yellow rectangle represents the downstream analyses also applied to the full set of studied samples.

The main aim of this study was to generate a robust species and subspecies-level phylogenetic reconstruction of the genus *Helianthemum* based on the analysis of paired-end GBS data. For this purpose, we conducted an extensive geographical and taxonomic sampling, including over 70% of the species and subspecies of *Helianthemum*, and representing all the supraspecific taxa (2 subgenera, 10 sections). Thus, our study provides the most comprehensive phylogenetic hypothesis for the genus *Helianthemum* and one of the largest trees reconstructed to date based on reduced-representation sequencing (e.g. Wagner et al., 2013; Ebel et al., 2015). This phylogeny was generated by following a rigorous methodological workflow (see **Figure 1**) in which we aimed to i) explore how bioinformatic decisions affect error rates (locus, allele and SNP error) and degree of resolution in phylogenetic inferences using concatenation and coalescent approaches; ii) assess the effects of two extreme configurations of assembly parameters (minimizing error rates vs. maximizing phylogenetic resolution) on tree topology and branch length estimation; and iii) evaluate the effects of these configurations on estimates of divergence times and diversification rates.

The robust phylogenetic framework here established provides, for the first time, the opportunity to address questions about the macroevolutionary patterns of the genus *Helianthemum*. Specifically, we tested if the large number of species and subspecies in the genus is the result of low extinction rates or, conversely, of recent and rapid independent radiations corresponding with the three largest sections. With the powerful insights provided by the molecular phylogenies comes the possibility of detecting rapid and recent radiations in particular groups based on three operational criteria: i) a recent common ancestor, ii) species-poor sister lineages, and iii) significant bursts of diversification (Nee et al., 1996; Sanderson and Donoghue, 1996; Pybus and Harvey, 2000; Schluter, 2000; Glor, 2010; Bouchenak-Khelladi et al., 2015). Since the recent common ancestry of each of the three largest sections of *Helianthemum*, as well as diversity asymmetries with their sister clades have already been suggested (Aparicio et al., 2017), here we aim to explore if significant bursts of diversification are detectable during the evolutionary history of the genus. In this regard, we asked: i) How high is the diversification rate in *Helianthemum* and in the three largest sections compared to other recently diversified Mediterranean lineages? ii) Is there any detectable acceleration of diversification rates in the course of *Helianthemum* evolution? If so, iii) do these accelerations correspond with the origin of the three largest sections and thus provide additional evidence of recent and rapid radiations? And iv) are these alleged independent radiations characterised by contrasting diversification patterns?

## Materials and Methods

### Taxon Sampling

One hundred and twenty-eight samples were used in this study (**Table S2**). The ingroup consisted of 98 taxa (73 species, 25 subspecies; 124 accessions; **Tables S2 and S3**) from the whole distribution range of the genus *Helianthemum*, including all supraspecific taxonomic ranks (2 subgenera, 10 sections). Given the large geographical and taxonomic scope, all species and subspecies were represented by a single sample each, except those belonging to monospecific or species-poor sections and those not included in the previous phylogenetic reconstruction of the genus (Aparicio et al., 2017), for which two samples were included. Replicates from three individual samples representing the three main lineages of *Helianthemum* (Aparicio et al., 2017; **Table S2**) were also included to optimize bioinformatic processing (see *Materials and Methods*, *Bioinformatics Workflow*). The outgroup consisted of four species belonging to other genera of Cistaceae, one representing an early-diverging lineage within the family (*Fumana*) and the other three (*Cistus*, *Halimium* and *Tuberaria*) representing the well-supported sister clade to *Helianthemum* (Aparicio et al., 2017). The inclusion of this outgroup enabled the implementation of two of the three fossil calibration points in the dating analysis (see *Materials and Methods*, *Downstream Analyses*). Except for four samples obtained from herbarium collections, all the plant material used in this study was freshly collected in the field from natural populations and stored in silica gel until DNA extraction (**Table S2**).

### DNA Extraction, Library Preparation and NGS

DNA was extracted from the silica-dried leaf material using the Bioline Isolate II Plant DNA Kit (Bioline, London, UK) following the manufacturer’s protocol. The concentration and quality of each sample were assessed using a Qubit dsDNA BR Assay kit (Thermo Fisher Scientific), and 260/280 and 260/230 absorbance ratios were measured on a NanoDrop spectrophotometer (Thermo Fisher Scientific). Paired-end genotyping-by-sequencing (PE GBS) multiplexed libraries were constructed and sequenced by CNAG (Centro de Análisis Genómicos, Barcelona, Spain) following the protocol used by Elshire et al. (2011) with improvements from Poland et al. (2012) and Sonah et al. (2013). The restriction enzyme *Ape*K1 was chosen for digestion of genomic DNA based on a small-scale experiment. Two lanes of Illumina HiSeq 2000, with a read length of 2x125bp, were used to increase sequencing coverage. Image analysis, base calling and quality scoring of the run were conducted using the manufacturer’s software Real Time Analysis (RTA 1.18.66.3), followed by generation of FASTQ sequence files by CASSAVA (see **Methods S1** for details).

### Bioinformatics Workflow

Due to the complexity of the proposed methodology, which contains three main steps (exploratory PyRAD assembly, final PyRAD assembly and downstream analyses) and several analyses within each one (error rate calculations, concatenated and coalescent phylogenetic analyses, branch length estimation, divergence time estimation and diversification rate analyses), the bioinformatics and analytical workflow followed in this study is summarized in **Figure 1**, based on Anderson et al. (2017).

#### Demultiplexing and Merging of Overlapping Reads

Demultiplexing was carried out using a custom script developed by CNAG in which GBS and Illumina barcodes as well as reads shorter than 25 bases were removed. The demultiplexed Illumina FASTQ reads were run on PEAR v. 0.9.8 (Zhang et al., 2014) to check for and merge overlapping reads using default settings except 33 bp as the minimum possible length of the assembled sequences (-n option) and 33 bp as the minimum length of reads after trimming the low quality part (-t option). Merging the reads is advisable to reduce duplication in the dataset and increase the reliability of each nucleotide position, especially at the ends of the reads which tend to have higher error rates (Eaton, 2014; Andrews et al., 2016; Anderson et al., 2017).

#### Exploratory PyRAD Assembly

Reads were assembled *de novo* using the PyRAD pipeline v. 3.0.6 (Eaton, 2014) since no reference genome was available for the family Cistaceae. Before the assembly, a quality filtering step was run in which bases with a FASTQ quality score below 20 were replaced with N and sequences having more than 4% of Ns were discarded. Merged and unmerged output files generated by PEAR were assembled and analysed separately by setting the data type to "merged" or "pairend" respectively in the PyRAD parameter file (parameter 11).

To determine the appropriate assembly settings, we followed the approach of Mastretta-Yanes et al. (2015) using replicates to assess the error rates associated with different parameter configurations (three pairs of replicates, six samples in total), as well as the approach used by Anderson et al. (2017) to analyse the impact of different parameter values on the degree of resolution of resulting phylogenetic trees in terms of number of supported nodes (see *Materials and Methods*, *Phylogenetic Analysis*). In particular, Mastretta-Yanes et al.'s approach was built on the idea that individual sample replicates (consisting of two DNA extractions from the same sample that are sequenced, processed and analysed independently), under the expectation of identical genotypes, allow the quantification of genotyping errors as the differences between replicates at the locus, allele, and SNP levels in the absence of a reference genome. Thus, locus error represents the number of loci missing from one replicate but not from the other relative to the total number of loci; allele error is the number of shared loci differing in sequence between the replicates relative to the total number of shared loci; and SNP error is the number of SNPs differing between replicates (hard error when differing in both alleles and heterozygous error when differing in one allele) relative to the total number of shared SNPs. Because replicates derived from the same DNA sample should have the same genotype, one can evaluate which parameter values of the assembly pipeline maximize the number of loci while minimizing differences between replicate pairs (see Appendix S1 from Mastretta-Yanes et al., 2015).

The bioinformatic parameters evaluated were the type of data (merged or unmerged), the clustering threshold, the base calling method (statistical base calling or majority-rule base calling), the minimum sample coverage and the minimum taxon coverage (Eaton, 2014; see **Methods S2** for details). All other parameters were set to default values. To reduce computing time and simultaneously allow a robust evaluation of assembly settings, these exploratory analyses were carried out for a subset of 70 samples representing all suprageneric taxonomic ranks. The subset was run through 15 parameter configurations (30 assemblies in total including merged and unmerged data): a minimum sample coverage of 2, 5, or 10 per individual locus, presence/absence of majority-rule base calling for low depth sites (from a minimum sample coverage below 5 or 10), clustering threshold at 85%, 90%, and a combination of 90% in step 3 (clustering within samples) and 85% in step 6 (clustering among samples). The minimum taxon coverage was kept at 15%. Locus error, allele error, and SNP (hard and heterozygous) error rates were calculated with modified python and R scripts used by Anderson et al. (2017) (scripts 5–7 contained in Supporting Information S3 of that article) and ape v. 3.3. (Paradis et al., 2004) for each of the three replicated samples and then averaged for each configuration.

#### Final PyRAD Assembly

We selected two extreme parameter configurations to analyse the full set of samples: the first one minimizing allele and SNP error rates (MinError configuration) and the second one maximizing phylogenetic resolution (MaxResol configuration). The latter was defined as the configuration that provided the highest number of supported nodes in phylogenetic analyses (see *Materials and Methods*, *Phylogenetic Analyses*). The resulting MinError configuration had a minimum sample coverage of 10, no majority-rule base calling, a clustering threshold of 90% and was based on merged data. The MaxResol configuration had minimum sample coverage of 10, majority-rule base calling, a clustering threshold of 85% and was based on merged data (**Table 1**). Both configurations were applied to the full set of samples, and outputs were generated at minimum taxon coverage values of 15, 25 and 50% (six assemblies in total; see **Methods S2** for details) to assess the impact of the amount of missing data on the degree of resolution (number of supported nodes), congruence between phylogenetic trees and branch length estimates (see *Materials and Methods*, *Phylogenetic Analyses*).

**Table 1:** Assembly information obtained from the exploratory PyRAD assembly using the subset of 70 samples.

PYRAD PARAMETERS				ASSEMBLY RESULTS										PHYLOGENETIC INFERENCES RESULTS	
Data type	Majority-rule base calling	Minimum sample coverage	Clustering threshold	Base pairs	Number of loci	Number of SNPs	N° of phylogenetically informative sites	% missing data	Locus error	Allele error	SNP error	Hard error	Het error	RaxML resolution	SVD quartets resolution
**Merged**	No	2	85	2509874	20565	448042	240782	69.10%	**0.0778**	0.1101	0.0053	0.0033	0.002	91.18%	**78.33%**
		2	90	2302252	19212	318202	152422	*70.50%*	0.0737	0.0901	0.0048	0.0024	0.0023	88.24%	70.00%
		2	90_85	2054634	17118	355695	190512	68.90%	0.0803	0.1	0.0044	0.0024	0.002	92.65%	*73.33%*
		5	85	1154519	9793	213781	116633	68.70%	0.1201	0.0852	0.0038	0.0022	0.0016	88.20%	*63.33%*
		5	90	1038637	8982	149295	72971	69.80%	0.1133	0.0657	0.0032	0.0016	0.0015	92.65%	63.33%
		5	90_85	1032374	8859	190199	104427	68.80%	0.1229	0.0792	0.0034	0.0019	0.0016	86.76%	71.67%
		10	85	484189	4210	90658	50295	67.80%	0.2014	0.0609	0.0042	0.003	0.0011	*78.00%*	76.67%
		10	90	*424790*	*3758*	*62176*	53795	68.40%	0.1866	**0.0421**	**0.0025**	**0.0014**	**0.0011**	79.40%	*63.33%*
		10	90_85	461589	4021	86212	*45637*	*67.80%*	*0.2065*	0.054	0.0028	0.001663	0.0011	79.41%	73.77%
	Yes	5	85	2717090	22238	504878	270162	69.10%	0.0808	0.1303	0.0073	0.0047	0.0026	97.06%	73.33%
		5	90	2468923	20496	355049	170002	70.40%	0.0737	0.1086	0.0069	0.0037	0.0032	91.18%	73.33%
		5	90_85	2207222	18313	393910	211306	68.80%	0.0798	0.1204	0.0066	0.0038	0.0028	94.12%	76.67%
		10	85	**3421305**	**28311**	**645213**	**3421305**	**67.40%**	0.0801	*0.1338*	*0.0092*	*0.0064*	0.0028	**100%**	73.33%
		10	90	3169491	26363	462983	230582	69.50%	0.0739	0.115	0.0092	0.0057	*0.0035*	94.12%	71.67%
		10	90_85	2668014	22254	477571	262214	67.50%	0.0803	0.1257	0.0082	0.0054	0.0027	91.18%	71.67%
**Unmerged**	No	2	85	472248	2135	69171	38676	71.00%	0.1717	0.1359	0.0057	0.0036	0.0021	**80.88%**	**66.67%**
		2	90	227656	1022	21605	11112	70.90%	**0.1494**	0.1186	0.0039	0.0016	0.0023	64.71%	53.33%
		2	90_85	410904	1858	58964	33289	70.70%	0.1713	0.1292	0.0036	0.002	0.0017	73.53%	63.33%
		5	85	59990	271	6613	3642	64.20%	0.1968	0.0714	0.011	0.0073	0.0037	44.10%	25.00%
		5	90	39745	179	2203	1094	59.40%	0.1639	0.0421	0.0027	0.0027	0	39.70%	18.33%
		5	90_85	55234	250	5822	55234	62.00%	0.212	0.0479	0.0038	0.0016	0.0022	47.06%	26.67%
		10	85	22353	101	1002	574	55.40%	0.1617	0.0263	0.0072	0	0.0072	41.50%	10.00%
		10	90	20399	*92*	*566*	*292*	57.10%	0.1558	0.0057	0	0	0	36.80%	*8.33%*
		10	90_85	*20359*	*92*	699	393	**55.10%**	0.1703	**0.0056**	0	0	0	*33.82%*	13.33%
	Yes	5	85	389645	3049	86228	47461	74.30%	0.2823	*0.2729*	0.0351	0.0306	*0.0044*	54.41%	43.33%
		5	90	145568	1120	23778	13160	72.60%	0.2604	0.1386	0.0195	0.0192	0.0003	50.00%	18.33%
		5	90_85	189955	1444	37927	21722	73.10%	0.2595	0.1819	*0.0351*	*0.0346*	0.0005	58.82%	38.24%
		10	85	**573516**	**5186**	**133793**	**573516**	74.30%	0.3011	0.1541	0.0252	0.0251	0.0001	70.58%	46.67%
		10	90	474711	4277	95182	53795	74.30%	*0.3013*	0.1437	0.0182	0.0182	0	63.24%	50.00%
		10	90_85	527721	4766	120850	69793	*74.40%*	0.3	0.1563	0.0221	0.0221	0	66.67%	48.33%

Numbers in italic indicate the worst values and numbers in bold indicate the best ones. The MaxResol configuration corresponds with minimum sample coverage (md) = 10 and majority-rule base calling under this coverage and clustering threshold (ct) = 85 from merged data. The MinError configuration corresponds with md = 10 and ct = 90 from merged data.

### Phylogenetic Analyses

To analyse the impact of assembly parameters (see *Materials and Methods*, *Bioinformatic workflow*) on phylogenetic resolution, we applied two phylogenetic methods to the subset assemblies resulting from the exploratory PyRAD analyses: a concatenated approach using maximum likelihood (ML) in RAxML 7.2.8 (Stamatakis, 2006) and a coalescent approach using the quartet-based method SVDquartets (Chifman and Kubatko, 2014) implemented in PAUP* 4 (Swofford, 2002). ML analyses were conducted using the GTR+GAMMA nucleotide substitution model. This widely used model was chosen because it usually fits real data better than other simpler alternative models (Sumner et al., 2012). At the same time it is practical for large data sets compared to more complex models (e.g. GMM by Barry and Hartigan, 1987; SBH and RBH models by Jayaswal et al., 2011). We applied a rapid bootstrap with automatic bootstrap stopping criterion and calculation of extended majority-rule consensus tree, followed by search for the best-scoring ML tree. No partition scheme was applied. The quartet-based method SVDquartets was selected given its computational efficiency, which makes it highly suitable for estimation of species trees of large taxon sets. The SVDquartets analysis was run under the multispecies coalescent using the concatenated alignment, evaluating one million quartets. One thousand bootstrap replicates were conducted and results were summarised in a 50% majority-rule consensus tree. After evaluating the degree of resolution provided by merged and unmerged data separately (see details below), we combined both types of data and checked whether this resulted in an improvement in phylogenetic resolution. Since no significant improvement was obtained and given that the error rates were substantially higher for unmerged data (see *Results*), we only analysed merged data for the full set of samples.

We performed the same analyses (RAxML and SVDquartets) for the two selected configurations (MinError and MaxResol) using the full set of samples under three values of the minimum taxon coverage parameter (15%, 25%, and 50%). We implemented an additional concatenated analysis using Bayesian inference (BI) in ExaBayes 1.4.1 (Aberer et al., 2014), as well as a further coalescent-based analysis using the NJst method (Liu and Yu, 2010). BI was implemented with the GTR+GAMMA substitution model and one or two runs (until convergence was reached) with four Metropolis-coupled Monte Carlo Markov Chains (MCMCs) each, and trees sampled every 500 generations for 500 000 generations. Convergence was assessed with Tracer 1.7.1 (Rambaut et al., 2018) using summary statistics calculated from the parameter files. We checked that a minimum value of 200 had been reached for the effective sample sizes (EES) of all parameters. Fifty-percent majority-rule consensus phylograms and posterior probabilities were obtained using the *consense* command with a burn-in fraction of 10%.

Amongst available summary methods accounting for ILS, we selected NJst because it is able to infer the species tree from unrooted gene trees (outgroup samples would be absent from many gene trees in our dataset, impeding the rooting of gene trees) and it can accommodate missing data. To build the species trees under the NJst method, we firstly estimated gene trees using RAxML with the GTR+GAMMA substitution model and 200 bootstrap replicates for all loci showing variability. One hundred multilocus bootstrap replicates (Seo, 2008; Mallo, 2015) were generated, thus resampling nucleotides within loci, as well as loci within the dataset. The NJst method was implemented on the one hundred bootstrapped matrices using the R script NJstM (Mallo, 2016), which relies on the phybase package (Liu and Yu, 2010). A 50% majority-rule consensus tree was then built from the 100 bootstrap replicates in PAUP* 4 (Swofford, 2002).

All phylogenetic analyses and the bioinformatic processing in PyRAD (see *Materials and Methods*, *Bioinformatics Workflow*) were performed using the computer clusters at the Centro Informático Científico de Andalucía (CICA, Seville, Spain) and the Consejo Superior de Investigaciones Científicas (cluster Trueno, CSIC, Madrid, Spain).

We evaluated the degree of resolution in the trees inferred from all parameter configurations (subset and the full set of samples) by calculating the quotient of the number of resolved nodes (bootstrap support BS > 70; posterior probability PP > 0.90; Hillis and Bull, 1993; Salichos et al., 2014), relative to the total number of nodes in the tree. Since traditional branch support metrics (BS, PP) present problems of tractability and interpretation when applied to phylogenomic datasets (Pease et al., 2018), we additionally implemented the recently developed Quartet Sampling (QS) method (Pease et al., 2018) using the MinError and MaxResol Bayesian trees. This method represents a generalized framework to quantify phylogenetic uncertainty (specifically branch support) that distinguishes branches with low information from those with multiple highly supported, but mutually exclusive, phylogenetic histories by calculating three metrics: Quartet Concordance (QC) score, Quartet Differential (QD) score, and Quartet Informativeness (QI) score (Pease et al., 2018). For each analysis, we ran 100 replicates per internal branch. We were most interested in QC, the frequency of quartets sampled that are concordant with the consensus tree.

For the full-set assemblies, we assessed the congruence among trees resulting from the two configurations following two approaches: i) by comparing Bayesian trees from ExaBayes (because of their highest resolution; see *Results*) using the relative Robinson–Foulds (RF) distance (Robinson and Foulds, 1981) and the Kuhner–Felsenstein branch score difference (BSc) (Kuhner and Felsenstein, 1994), calculated with the "RF.dist" and "KF.dist" functions of the R package phangorn v. 2.5.3 (Schliep, 2011); and ii) by visually inspecting incongruent placements of individual samples or whole clades (Pirie, 2015). Finally, we evaluated the potential influence of error rates and proportion of missing data (resulting from the three values of minimum taxon coverage: 15%, 25%, and 50%) on branch length estimates in the RaxML and ExaBayes trees for the full-set assemblies and the two extreme configurations. Thus, for each tree we calculated median values of terminal branch lengths and median values of internal branch lengths divided by the total branch length of the tree (relative branch lengths) using ape v. 3.3 (Paradis et al., 2004). The R package ggplot2 v.3.1.1 (Wickham, 2009) was used to visualize the results.

### Downstream Analyses

#### Divergence Times

Divergence times were estimated using the penalized likelihood (PL) approach implemented in the program TreePL v. 1.0 (Smith and O'Meara, 2012). Penalized likelihood (Sanderson, 2002) uses a tree with branch lengths and age constraints for time calibration without prior parametric distributions. It considers rates to be auto-correlated and further accounts for among-branch rate heterogeneity, using a so-called smoothing parameter (Sanderson, 2002). TreePL is a modiﬁed and speed-enhanced version of the program r8s (Sanderson, 2003) using stochastic optimization and hill-climbing gradient-based methods, more suitable for very large data sets. We utilized TreePL because most other approaches for divergence time estimation (e.g. the uncorrelated lognormal relaxed clock approach in BEAST; Drummond et al., 2006; Drummond and Rambaut, 2007) would not be practical given the large number of taxa and loci analysed here.

We used the phylogenetic trees resulting from ExaBayes as input (except that resulting from the MinError configuration under 50% minimum taxon coverage due to its low resolution). As penalized likelihood does not automatically provide conﬁdence intervals, we conducted the analysis using the majority-rule consensus trees resulting from the Bayesian analyses in ExaBayes (see above) and 900 trees from the Bayesian distribution of the same analyses after a 10% burnin. Trees were pruned to include only one terminal per species. A "priming" analysis was first conducted to optimize the set of parameters. Based on these results, the values of gradient-based, auto-differentiation-based, and auto-differentiation cross-validation-based optimizers were all set to two.

For the implementation of fossil calibration points, PL approaches need either a deﬁned ﬁxed age of a node, or a minimum and/or a maximum age constraint on a node. We applied four minimum and maximum age constraints as calibration points (N1: stem node of genus *Tuberaria*, min = 3.02 Myr, max = 10.53 Myr; N2: stem node of genus *Helianthemum*, min = 7.07 Myr, max = 23.86 Myr; N3: crown node of genus *Helianthemum*, min = 3.56 Myr, max = 14.08 Myr; and N4: stem node of *Helianthemum nummularium* complex, min = 0.32, max = 3.61). The minimum ages used in N1, N2, and N4 are fossil-based age constraints (Naud and Suc, 1975; Menke, 1976; Hrynowiecka and Winter, 2016) while the maximum ages in those calibration points as well as the minimum and maximum ages used in N3 are estimates obtained from a previously-published dated phylogeny of Cistaceae (Aparicio et al., 2017) using BEAST (Drummond et al., 2012).

The analysis was set to be thorough to make sure that it continued to iterate until convergence. We selected a smoothing parameter with values between 1x10^-199^ and 1x10^-9^ depending on the tree, following the random subsample and replicate cross-validation approach (RSRCV) as implemented in TreePL, in which 235 values from 1x10^-226^ to 1x10^8^ were tested. RSRCV produces similar results to those using standard cross-validation (i.e. removing one taxon), but is capable of handling trees with thousands of taxa within a reasonable time frame (Smith and O'Meara, 2012). The chronograms resulting from the 900 Bayesian trees were then summarized with TreeAnnotator v1.7.5 (Drummond et al., 2012), and 95% conﬁdence intervals were represented on the chronogram resulting from the majority-rule consensus tree to incorporate topological and branch length uncertainty.

#### Diversification Rates

First, we estimated absolute net diversification rates for the genus *Helianthemum* and for the three largest sections, and compared them with the most rapid episodes of hyper-diversification reported for other Mediterranean plant lineages (Vargas et al., 2018). We used the standardized whole-clade method of Magallón and Sanderson (2001) implemented in the R package geiger v. 2.0.6.1 (Harmon et al., 2008). Rates were calculated for the mean crown ages obtained from a previously published chronogram (Aparicio et al., 2017) because these ages were estimated using a Bayesian relaxed clock analysis of specific DNA regions obtained by Sanger sequencing, as in most of the other Mediterranean examples used here for comparison.

Secondly, we applied a Bayesian approach implemented in BAMM v. 2.5.0 (Bayesian analysis of macroevolutionary mixtures: Rabosky et al., 2013; Rabosky et al., 2014a; Shi and Rabosky, 2015) to detect significant changes in diversification dynamics (speciation and extinction rates). A significant increase in diversification rate is considered an evidence of the initiation of a radiation (Bouchenak-Khelladi et al., 2015). BAMM uses 'reversible jump' Markov chain Monte Carlo (rjMCMC) to account for rate variation through time and among lineages (Rabosky, 2014). BAMM was applied using both TreePL chronograms and MCMC analyses were run with four chains for 10x10^6^ generations, sampling every 5000 generations. To account for the non-random sampling of our data set, we assigned sampling fractions at section level (**Table S3**). The prior distributions on speciation (λ) and extinction (µ) rates were estimated with the R package BAMMTOOLS v. 2.1.0 (Rabosky et al., 2014b) using the ‘setBAMMprior’ command. Likewise, calculation of ESS for the log-likelihood and the number of shift events, as well as post-run analyses and visualization of results were conducted with BAMMTOOLS. Diversification rate variation among the clades of our *Helianthemum* tree was evaluated with the following approaches: i) mean diversification rates at any point along every branch of the tree were displayed as a phylorate plot, ii) the best overall shift configuration was estimated as the maximum shift credibility (MSC) configuration, which maximizes the marginal probability of rate shifts along individual branches, and iii) speciation rates of the three largest sections were visualized as rate-through-time plots.

## Results

### Exploratory and Final PyRAD Assemblies

The number of read pairs, the number of merged, unmerged and discarded reads in PEAR and the number of loci recovered in PyRAD for each sample under both parameter configurations are shown in **Table S4**. The total number of loci recovered from the *exploratory PyRAD assembly* using the subset of 70 samples ranged from 3758 to 28311 in merged datasets and from 92 to 5186 in unmerged datasets, demonstrating the dramatic effect of parameter selection on the amount of resulting data (**Table 1**). In particular, the number of SNPs and PIS (phylogenetically informative sites) in the assembly decreased as the minimum sample coverage and clustering threshold increased. The implementation of majority-rule base calling resulted in larger datasets than statistical base calling alone. The recovered error rates based on three replicate samples also varied considerably (**Table 1**). In this case, as minimum sample coverage increased, locus error rates increased and allele and SNP error rates decreased. Furthermore, a similarity threshold of 90% always recovered error rates lower than those obtained under the 85% threshold and under the combination of 90% in step 3, and 85% in step 6. Finally, error rates were always lower in analyses of merged data than in analyses of unmerged data under the same parameter values (**Table 1**).

Regarding the full-set assemblies, the proportion of missing data varied between 33.7%, and 77.1%; fewer missing data were recovered as the minimum taxon coverage increased (**Table 2**). In the same way, the number of SNPs and PIS decreased as the minimum taxon coverage increased, especially from 25% to 50%. Lastly, although locus error increased with increasing minimum taxon coverage, allele and SNP error rates decreased.

**Table 2:** Characteristics of assembled genotyping-by-sequencing datasets from the *final PyRAD assembly*.

			MaxResol configuration			MinError configuration		
			MinCov15%	MinCov25%	MinCov50%	MinCov15%	MinCov25%	MinCov50%
**Assembly information**		Number of bp	3596013	1263524	239766	630754	158884	31706
		Number of loci	30351	10968	2214	5768	1471	295
		Number of SNPs	735769	309885	71477	96241	27130	4191
		Number of PIS	409337	182405	46097	47402	14055	2349
		Number of singleton sites	265805	102808	19809	27865	6954	891
		Percentage of missing data	74.40%	60.30%	34.70%	77.10%	61.10%	33.70%
**Error rates**		Locus error	0.0718	0.0889	0.1101	0.1450	0.1981	0.1718
		Allele error	0.1274	0.1089	0.0849	0.0408	0.0291	0.0133
		SNP error	0.0086	0.0063	0.0053	0.0022	0.0014	0.0006
		Hard error	0.0062	0.0045	0.0040	0.0012	0.0006	0.0006
		Heterozygous error	0.0024	0.0018	0.0013	0.0011	0.0008	0.0000
**Phylogenetic analyses**	RAxML	Resolution	95.04%	96.69%	90.08%	79.34%	62.81%	48.76%
		Total branch length	1.8256	1.7033	1.2817	0.7288	0.5218	0.2451
		Mean branch length	0.0073	0.0068	0.0051	0.0029	0.0021	0.0010
	ExaBayes	Resolution	94.21%	100%	98.35%	97.52%	86.78%	52.89%
		Total branch length	1.8197	1.7007	1.2846	0.7311	0.5271	0.2599
		Mean branch length	0.0073	0.0068	0.0051	0.0029	0.0021	0.0010
	SVDquartets	Resolution	77.69%	76.03%	71.70%	58.68%	49.59%	24.79%
	NJst	Resolution	82.65%	79.59%	54.98%	30.93%	26.80%	14.43%

Assembly information obtained from the final PyRAD assemblies using the full set of 126 taxa. Error rates and phylogenetic analysis information were obtained from two extreme parameter configurations (MaxResol, maximizing phylogenetic resolution; and MinError, minimizing error rates) under three minimum taxon coverage percentages (15, 25 and 50%). SNP, single-nucleotide polymorphism. PIS, phylogenetically informative sites.

### Phylogenetic Analyses

#### Degree of Resolution, Congruence and Branch Length Estimation

**Figure 2:**
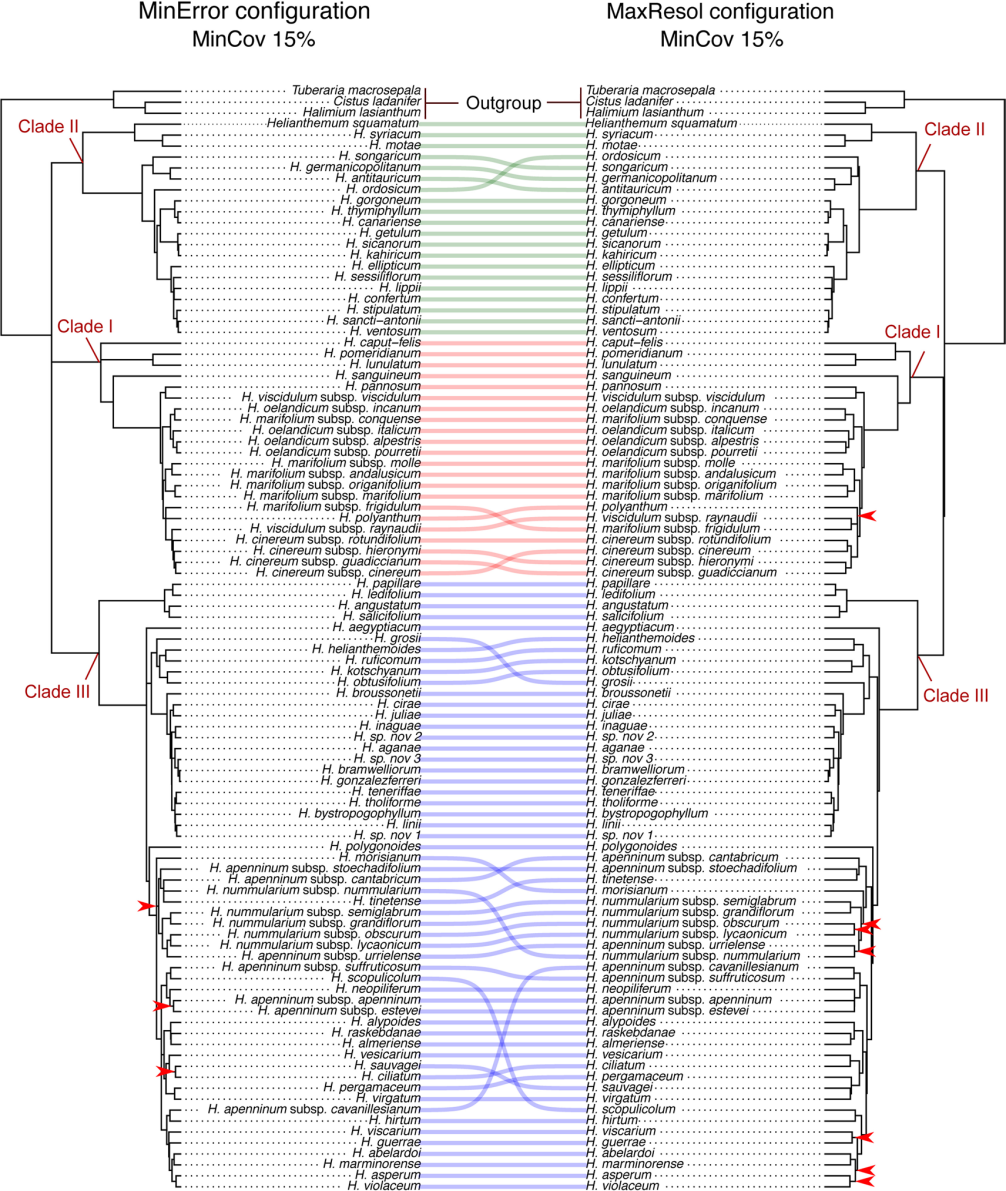
Comparison of 50% majority-rule consensus trees resulting from Bayesian analyses of *Helianthemum* GBS data in ExaBayes using the two extreme parameter configurations (MaxResol, maximizing phylogenetic resolution; and MinError, minimizing allele and SNP error rates) under 15% minimum taxon coverage. Red arrows indicate unsupported clades (PP < 0.95). Supported incongruences between analyses are highlighted with defined coloured lines, green in Clade II, red in Clade I, and blue in Clade III. Clades I, II and III are coincident with those in Aparicio et al., 2017.

**Figure 3:**
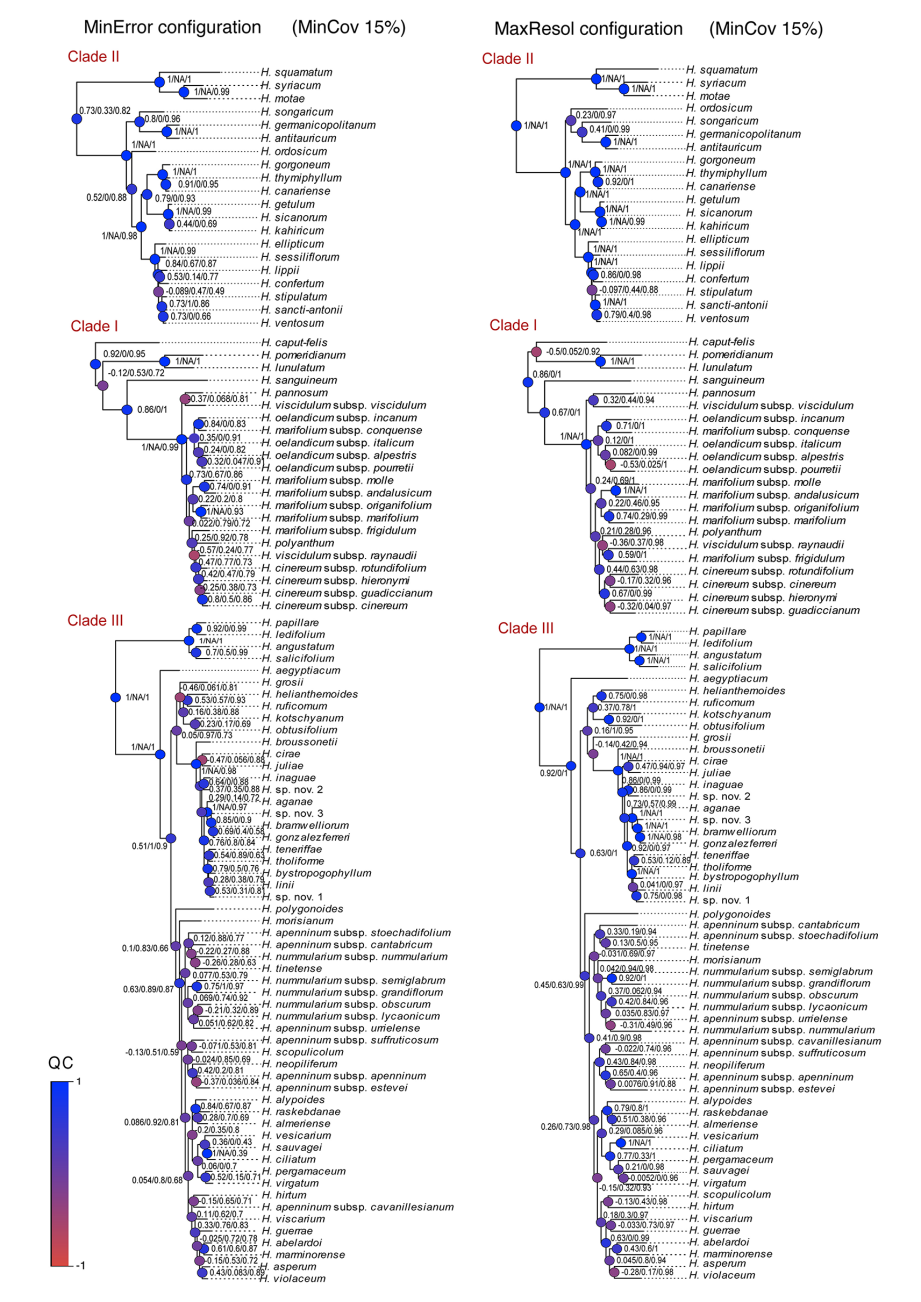
Quartet sampling score for branches of the two Bayesian trees generated under the extreme parameter configurations (MaxResol, maximizing phylogenetic resolution; and MinError, minimizing allele and SNP error rates) under 15% minimum taxon coverage. Scores shown for each branch are in this order, QC/QD/QI. Node are coloured according to QC scores. Clades I, II and III are coincident with those in Aparicio et al., 2017.

Phylogenetic method, data type (merged vs. unmerged), minimum sample coverage and minimum taxon coverage all significantly impacted the degree of resolution of phylogenetic trees (**Tables 1** and **2**). Tree resolution resulting from the concatenated analyses was higher than that obtained from coalescent analyses, especially in sects. *Pseudocistus* and *Helianthemum* (see below), and improved as the amount of data increased. In particular, MaxResol configuration assemblies recovered a higher degree of resolution in most of the analyses than MinError configuration assemblies. In the same way, the minimum taxon coverage parameter had a serious effect on the degree of resolution, particularly for the smallest assembly (MinError configuration, minimum taxon coverage = 50%), in which there was essentially no resolution within the three largest sections of the inferred phylogeny, probably due to a dramatic loss of phylogenetic information (**Table 2**). However, the MinError configuration yielded well-resolved phylogenetic trees under the two concatenation methods when minimum taxon coverage was 15% (RAxML: 79.34%; ExaBayes: 97.52%), which does not differ greatly from the results under the MaxResol configuration (RAxML: 90.00%; ExaBayes: 97.87%) (**Figure S1**). The exceptions were some minor incongruences that were well supported based on BS and PP metrics and mainly involved shallow nodes within sects. *Helianthemum* and *Pseudocisuts* (**Figure 2**). Consistent with these incongruences, the quartet sampling analyses displayed negative QC scores for these conflictive nodes (**Figure 3**). Negative scores imply that one of the discordant topologies is the most commonly resampled quartet. Despite these few topological discordances, QC and QI scores were high for most of the nodes, indicating a generally robust phylogenetic inference in both configurations and a strong topological consensus between them.

**Figure 4:**
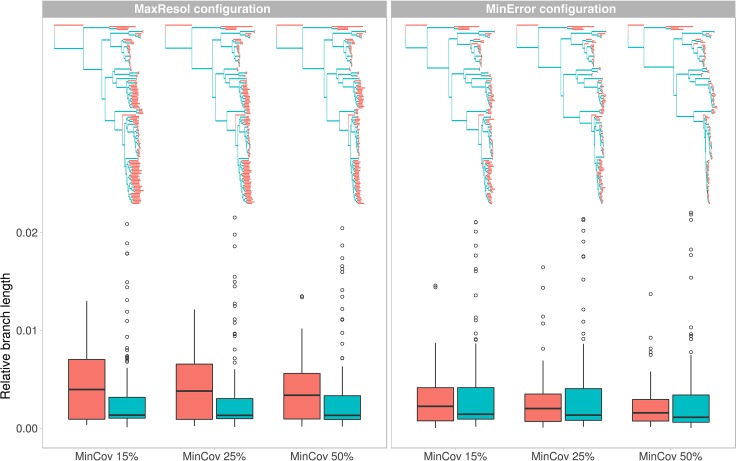
Phylogenetic trees and variation in relative branch lengths (shown as boxplots) resulting from two extreme parameter configurations (MaxResol, maximizing phylogenetic resolution; and MinError, minimizing allele and SNP error rates) under three minimum taxon coverage percentages (MinCov 15%, 25%, and 50%) used in the assembly of GBS data of *Helianthemum*. Red represents terminal branches while blue represents internal branches.

Total and mean branch lengths were substantially higher for the MaxResol than for the MinError configuration, and decreased as minimum taxon coverage increased for both configurations (**Table 2**). However, relative internal branch lengths stayed essentially constant across assemblies while relative terminal branch lengths were considerably longer under MaxResol than under MinError (**Figure 4**).

RF distances between assemblies within the MaxResol configuration were lower than within the MinError configuration or between assemblies from different configurations (**Table 3A**). BSc distances, a more appropriate measure in our context (because it takes branch length differences into account), were lower between assemblies within the MaxResol and MinError configurations than between assemblies from different configurations (**Table 3B**).

**Table 3:** Robinson Foulds (RF) and Branch Score (BSc) distances between Bayesian trees from MinError and MaxResol assemblies estimated in ExaBayes.

(A) Robinson Foulds (RF) distances.							
		MaxResol			MinError		
		MinCov15%	MinCov25%	MinCov50%	MinCov15%	MinCov25%	MinCov50%
MaxResol	MinCov15%						
	MinCov25%	32					
	MinCov50%	36	20				
MinError	MinCov15%	50	56	54			
	MinCov25%	78	80	78	60		
	MinCov50%	110	110	116	100	90	
(B) Branch Score (BS) distances							
		MaxResol			MinError		
		MinCov15%	MinCov25%	MinCov50%	MinCov15%	MinCov25%	MinCov50%
MaxResol	MinCov15%						
	MinCov25%	0.0294					
	MinCov50%	0.0569	0.0428				
MinError	MinCov15%	0.1130	0.1081	0.0716			
	MinCov25%	0.1311	0.1267	0.0892	0.0223		
	MinCov50%	0.1612	0.1582	0.1211	0.0550	0.0350	

Overall, tree topology and branch length estimates were more affected by parameter configuration (defined by base calling method, minimum sample coverage and clustering threshold) than by the amount of missing data (dependent on the minimum taxa coverage) (**Figure 4**; see **Methods S2** for more details regarding definition of PyRAD patameters).

#### The Most Robust Configuration

Even though the MaxResol configuration provided a higher degree of phylogenetic resolution than the MinError configuration under the three percentages of minimum taxon coverage (15%, 25%, and 50%; **Figure S1**, **Table 2**), MaxResol trees had high allele and SNP error rates (between four and 10 times higher than under MinError, **Table 2**), which can presumably bias terminal branch lengths (**Figure 4**). This bias would have an adverse effect on downstream analyses (**Figures S2–S4**). On the other hand, the MinError configuration under minimum taxon coverages of 25 and 50% retrieved some relationships that were biologically unreasonable and incongruent with those obtained from the rest of the assemblies, probably due to an extreme loss of phylogenetic signal in samples with a low starting number of reads (e.g. *H. sauvagei*, *H. kotschyanum*, *H. nummularium* subsp. *lycaonicum*; **Tables 3A, B**; **Figure S1**; **Table S4**).

**Figure 5:**
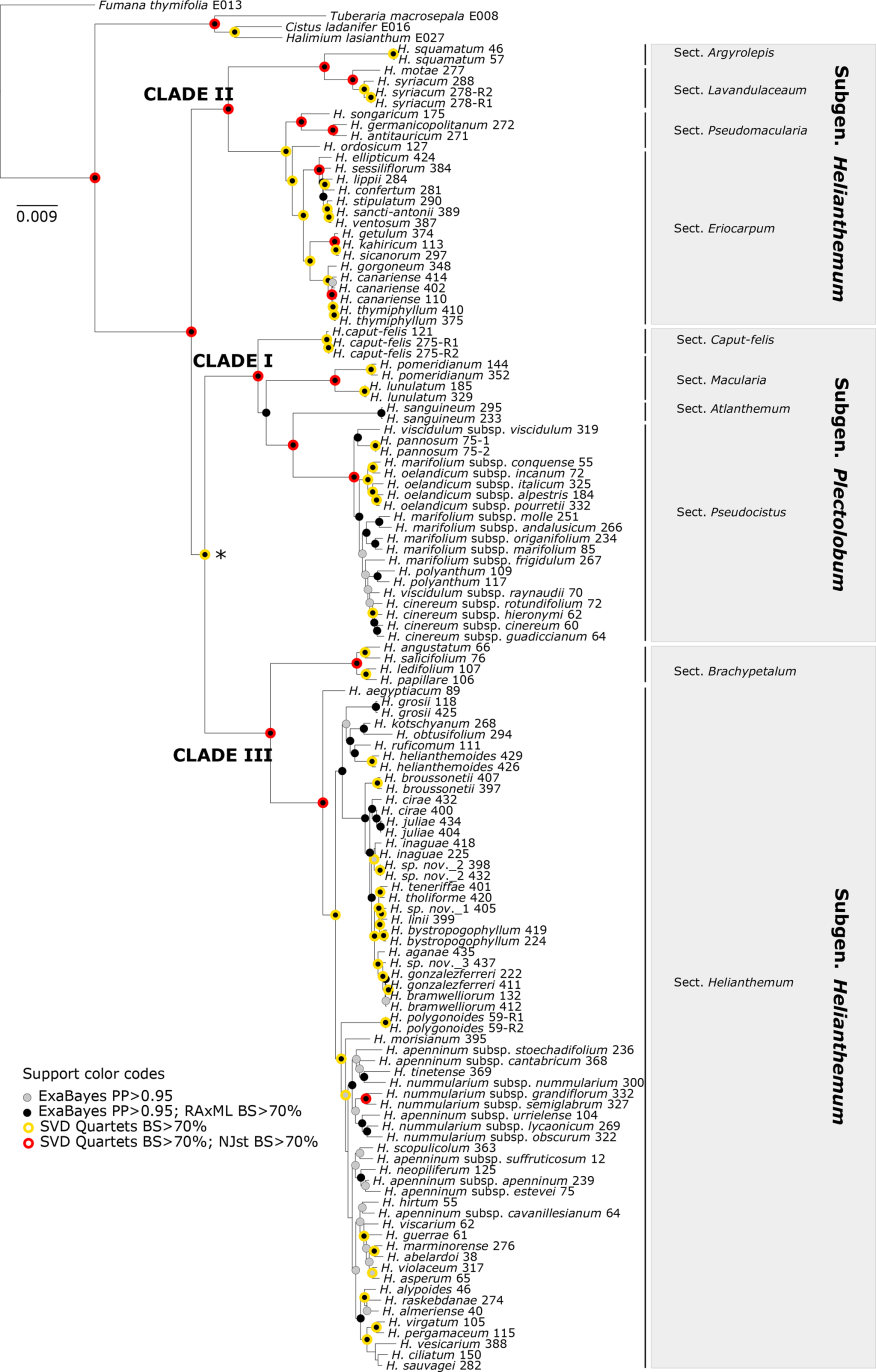
The 50% majority-rule consensus tree obtained from Bayesian analysis of *Helianthemum* GBS data in ExaBayes using the most robust assembly (MinError configuration under 15% minimum taxon coverage). Circles of different colours indicate clades that are supported in the two concatenated (ExaBayes, RAxML) and the two coalescent (SVDquartets, NJst) phylogenetic analyses. The intrageneric taxonomic assignments of taxa (sections and subgenera) follow López-González (1993) and Aparicio et al. (2017). The asterisk denotes the single clade for which NJst provided high bootstrap support but SVDquartets did not. There were no clades with RAxML BS > 70 but ExaBayes PP > 0.95.

Overall, we considered that the most robust species-level phylogenetic tree—taking into account degree of resolution, topological congruence with MaxResol assemblies and reliability of branch length estimation—was the phylogenetic tree resulting from the MinError configuration assembly under a minimum taxon coverage of 15% (**Table 2**, **Figures 2**–**5**). This tree was selected as a suitable phylogenetic framework for downstream evolutionary analyses.

#### Phylogenetic Relationships

Despite the different degrees of phylogenetic resolution and minor incongruences obtained under the broad set of configurations and assemblies tested (**Table 2**, **Figure S1**), all the methods carried out in the present study consistently recovered similar tree topologies consisting of three main clades (I, II, and III). Interestingly, these three clades all had a similar internal structure, namely, one species-rich subclade coinciding with the larger sects. *Eriocarpum* (thereafter referred to in this paper as *Eriocarpum s.l.* in order to include its small sister section *Pseudomacularia*), *Pseudocistus* and *Helianthemum* in clades II, I, and III, respectively, accompanied by one or a few poorly diversified subclades consisting of the monospecific or species-poor sects. *Argyrolepis* and *Lavandulaceum* in clade II, *Caput-felis*, *Macularia*, and *Atlanthemum* in clade I, and *Brachypetalum* in clade III (**Figure 5**). In our reconstructions, clades II and III correspond taxonomically to subgenus *Helianthemum* and clade I to subgenus *Plectolobum*. Nomenclature and taxonomic adscriptions of taxa follow López-González (1993), but also take into account the supported systematic implications of the phylogenetic reconstruction obtained by Aparicio et al. (2017).

### Downstream Analyses

#### Divergence Times

The extremely low values of the smoothing parameter estimated from most assemblies using TreePL (1x10^-199^ to 1x10^-9^) indicated non-clock-like rates. All analyses recovered very narrow conﬁdence intervals due to the low branch length variability among the 900 Bayesian trees obtained from each assembly (**Figure S2**). However, the estimated ages differed substantially between configurations and assemblies. The MaxResol configuration analysis yielded much more recent ages for the deepest nodes and older ages for shallow nodes when compared to the MinError configuration analysis (**Figures S2 and S4**).

#### Diversification Rates

The overall net diversification rate of the genus *Helianthemum* (r = 0.50) was of medium magnitude, comparable to those of other Mediterranean lineages such as *Antirrhinum* (r = 0.56), *Erodium* (r = 0.20), *Genista* sect. *Spartocarpus* (r = 0.22), *Linaria* sect. *Versicolores* (r = 0.35), *Narcissus* (r = 0.17), and *Ophrys* (r = 0.55). However, net diversification rates in the three largest sections (sect. *Eriocarpum s.l*.: r = 1.11; sect. *Pseudocistus*: r = 1.26, and sect. *Helianthemum:* r = 1.61) were similar to those of some of the most rapid plant radiations in the Mediterranean Floristic Region reported to date, for example the white-flowered *Cistus* (r = 1.72), *Linaria* sect. *Supinae* (r = 1.55), the western European clade of *Erysimum* (r = 1.59), and *Reseda* sect. *Phyteuma* (r = 1.05) (see **Table 4**).

**Table 4:** Diversification rates of several species-rich plant clades from the Mediterranean Basin, including the genus *Helianthemum* and its three largest sections *Eriocarpum*, *Pseudocistus,* and *Helianthemum*.

	Number of species	Crown age	Diversification rate	Distribution range	Family
***Helianthemum***	104	7.80 (3.56-14.08)	Medium (0.50)	Mediterranean, Macaronesia, Saharo-Arabian, Irano-Turanian	Cistaceae
**Sect.** *** Pseudocistus***	17	1.70 (0.72–3.32)	**Fast (1.26)**	Mediterranean, Eurosiberian	
**Sect. ** ***Eriocarpum***	28	2.37 (1.01–4.63)	**Fast (1.11)**	Saharo-Arabian, Irano-Turanian, Macaronesia (Mediterranean)	
**Sect. ** ***Helianthemum***	47	1.91 (0.80–3.61)	**Fast (1.61)**	Mediterranean, Eurosiberian, Macaronesia	
*Antirrhinum* (Vargas et al., 2009)	20*	4.1	Medium (0.56)	W Mediterranean	Plantaginaceae
*Aquilegia* (European clade) (Fior et al., 2013)	25*	1.77 (0.97–2.57)	**Fast (1.47)**	S Europe	Ranunculaceae
*Cistus* (white-flowered) (Guzmán et al., 2009)	12	1.04 (0.06–1.41)	**Fast (1.72)**	Mediterranean	Cistaceae
*Dianthus* (Eurasian clade) (Valente et al., 2010)	200*	1.76 (1.09–2.43)	**Very fast (2.62)**	Mediterranean	Caryophyllaceae
*Erodium* (Fiz-Palacios et al., 2010)	74	18.34 (9.9–18.46)	Medium (0.20)	Mediterranean	Geraniaceae
*Erysimum* (W European clade) (Moazzeni et al., 2014)	25*	1.59 (0.74–2.43)	**Fast (1.59)**	W Europe	Brassicaceae
*Genista* sect. *Spartocarpus* (Fiz-Palacios and Valcárcel, 2013)	11	7.71 (7.18–8.23)	Medium (0.22)	C Mediterranean	Fabaceae
*Linaria* sect. *Supinae* (Blanco-Pastor et al., 2012)	44	2.0 (0.80–3.2)	**Fast (1.55)**	Mediterranean	Plantaginaceae
*Linaria* sect. *Versicolores* (Fernández-Mazuecos and Vargas, 2011)	30	7.73 (4.13–11.75)	Medium (0.35)	Mediterranean	
*Narcissus* (Santos-Gally et al., 2011)	70*	21.4 (16.1–27.4)	Medium (0.17)	Mediterranean	Amaryllidaceae
*Ophrys* (Breitkopf et al., 2015)	30*	4.9 (2.9–7.1)	Medium (0.55)	Mediterranean	Orchidaceae
*Reseda* sect. *Phyteuma* (Escudero et al., 2018)	16	1.98	**Fast (1.05)**	Mediterranean	Resedaceae

Number of species, crown age, diversification rates, distribution range and family are indicated for each clade. Diversification levels (slow, r < 0.1; medium, 0.1 < r < 1; fast, r > 1; see Vargas et al., 2018) are based on diversification rates calculated using Magallón and Sanderson’s method based on the number of species and mean estimated crown age. Asterisks indicate uncertainty regarding species numbers. Numbers in bold represent fast or very fast diversification rates.

**Figure 6:**
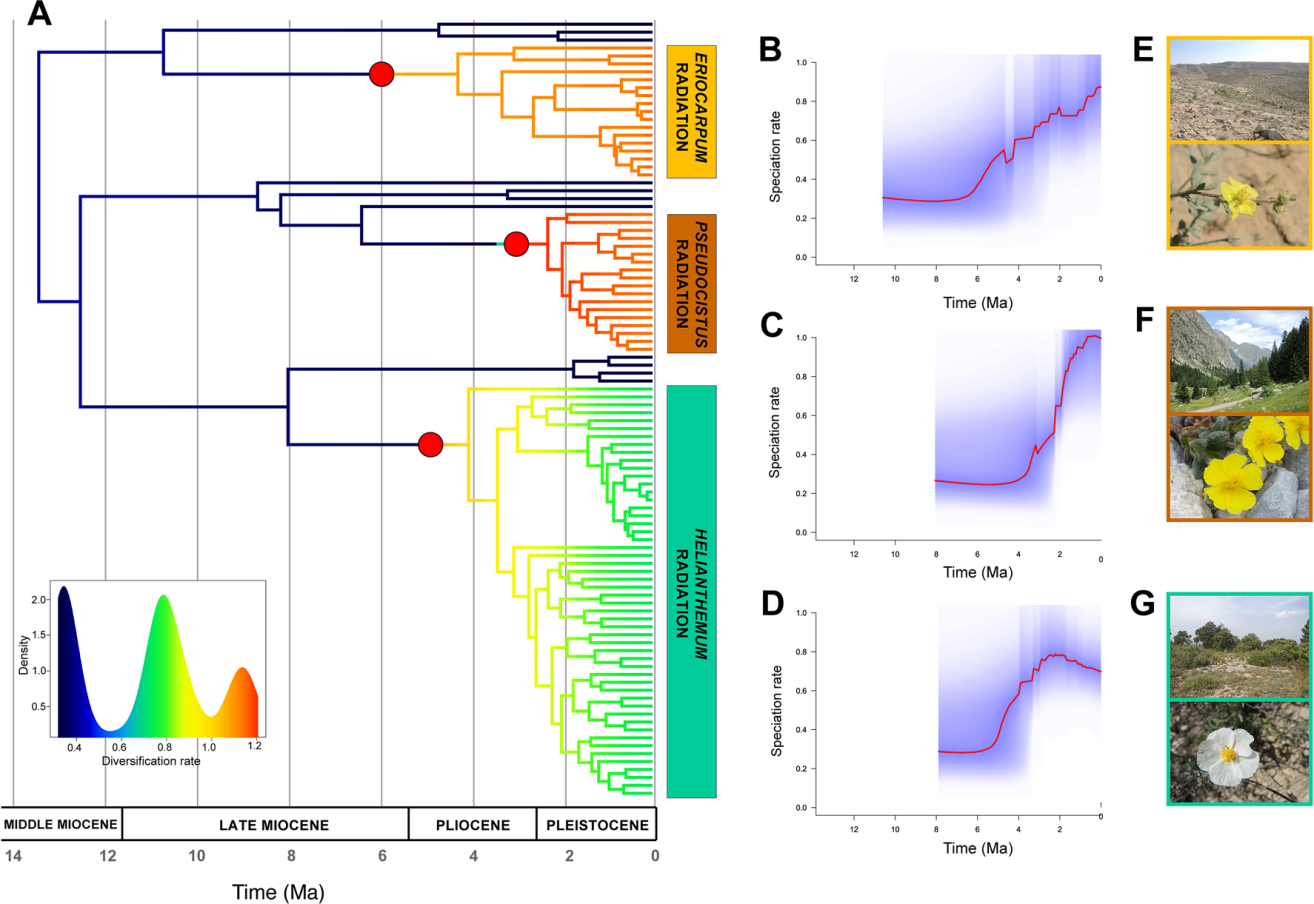
Diversification rates in *Helianthemum* based on GBS data. **(A)** Time-calibrated phylogenetic tree obtained in TreePL from the most robust assembly (MinError configuration under 15% minimum taxon coverage), with branches coloured according to diversification rates estimated using Bayesian Analysis of The performance of the research Macroevolutionary Mixtures (BAMM). Red circles at the base of the three largest sections (*Eriocarpum s.l.*, *Pseudocistus* and *Helianthemum*) mark the three diversification rate shifts initiating three evolutionary radiations. The insert shows the density of rate values across the phylogeny. **(B**–**D)** Speciation rates over time estimated by BAMM for each radiation, starting from their respective stem nodes. **(E**–**G)** Representative species and ecosystems of the three radiations: **(E)** *Helianthemum sessiliflorum* (Desf.) Pers. in Negev Desert (Israel); **(F)** *H. oelandicum* subsp. *alpestre* (Jacq.) Ces. of alpine pastures in Alpes-Maritimes (France); **(G)** *H. apenninum* (L.) Miller subsp. *apenninum* in Mediterranean maquis at Pico Ñoño Martés (Spain).

The diversification patterns estimated from BAMM analyses differed dramatically between configurations. MaxResol chronograms recovered no significant shifts in diversification rates in the tree, whilst MinError chronograms displayed very heterogeneous diversification dynamics in *Helianthemum* (**Figures 6**, **S4**). In particular, the MinError configuration produced three significant shifts to increased rates of speciation (λ) relative to background levels in the genus (λ = 0.5). The first shift was inferred at the base of sect. *Eriocarpum s.l.* (λ = 0.90; 4.20 Ma), with constant speciation over time from the stem to the present. The second and third shifts occurred at the base of sect. *Helianthemum* (λ = 0.76; 3.4 Ma) and at the base of sect. *Pseudocistus* (λ = 1.06; 2.25 Ma), characterized by exponential bursts of speciation followed by stasis or a slight drop (**Figure 6**).

## Discussion

Compared to the previous phylogenetic reconstruction of the genus *Helianthemum* using Sanger sequencing, in which species and subspecies were mostly recovered in polytomies (Aparicio et al., 2017), here we generated a much more robust species and subspecies-level phylogenetic tree incorporating high geographical and taxonomic representativeness, strong statistical support for taxon relationships, and accurate estimates of tree topology and branch lengths. This has been achieved following an exhaustive methodological workflow specially designed to analyse a large amount of GBS data from this recently diversified lineage. We dealt with numerous methodological challenges and concluded that minimizing error rates produces more robust phylogenetic trees than maximizing phylogenetic resolution, affecting the accuracy of downstream macroevolutionary analyses. Moreover, our phylogenetic hypothesis has important implications from both systematic and evolutionary standpoints, and provides strong support for the existence of three major lineages in *Helianthemum* that have independently radiated since the Upper Miocene in contrasting geographical and ecological contexts.

### Effects of Bioinformatic Parameters on Topology and Branch Lengths

The choice of an optimal bioinformatic parameterization in phylogenomics is not straightforward due to the trade-offs between the number of loci and SNPs recovered and the error rates estimated from an assembly, especially when studying recently diversified lineages (Anderson et al., 2017). To date, most studies focussing on resolving phylogenetic relationships of recently diversified clades using GBS or RADseq data have tended to maximize the number of SNPs in order to increase the amount of phylogenetic information contained in the assembly (Wagner et al., 2013; Hou et al., 2015; Wessinger et al., 2016; Tripp et al., 2017; Lee et al., 2018). In our study, the resolution of the inferred tree topologies also increased dramatically as the data matrix increased in size, despite the concomitant increase in missing data. Thus, topologies received higher support for MaxResol configuration assemblies (both in concatenation and in coalescent methods), which contain more SNPs and PIS, than for MinError datasets (**Table 2**). Furthermore, the variation in the amount of missing data did not strongly affect tree topologies when the size of the assembly was high, particularly in the MaxResol configuration, since phylogenetic trees under the three minimum taxon coverage percentages and under the two phylogenomic approaches proved to be highly congruent (**Tables 3A, B**; **Figure S1**). This result is consistent with previous observations to the effect that large amounts of missing data in reduced-representation sequencing datasets do not adversely affect the accuracy of phylogenetic inference (Rubin et al., 2012; Takahashi et al., 2014; Hou et al., 2015; Herrera and Shank, 2016; Eaton et al., 2017; Lee et al., 2018). By contrast, some incongruent relationships were retrieved among the three assemblies under the MinError configuration, with ever-decreasing biological sense as the minimum taxon coverage increased, probably due to an excessive loss of phylogenetic information from samples with a low initial number of reads (**Tables 3A, B**; **Figure S1**, **Table S4**).

Although great efforts are usually devoted to maximizing the number of SNPs in order to optimize phylogenetic resolution, the effects of error rates on phylogenetic inference are rarely explored (Clark and Whittan, 1992; Lemmon et al., 2009). NGS methods may generate twice as many sequencing errors as Sanger sequencing (Ewing and Green, 1998; Wang et al., 2012; Glenn, 2014) and reduced-representation sequencing methods are prone to a number of additional sources of error. The effects of allele and SNP errors on population genetic inferences seem to be clear, and include an inflation of nucleotide diversity and a skewing of the SNP frequency spectrum towards rare SNPs (Ho et al., 2005; Johnson and Slatkin, 2008; Pool et al., 2010). These complications can hinder a biologically meaningful interpretation of population genetic data. However, there is a lack of consensus on how error rates bias phylogenetic reconstructions, with some authors noting that confidence in a tree depends on the sequencing error rate (Clark and Whittan, 1992) and others suggesting that error rates may be less detrimental for phylogenetics than for population genetics (Anderson et al., 2017). In our study, the generally congruent topologies obtained under both parameter configurations (**Figures 2** and **3**) suggest that the differential error rates resulting from applying contrasting bioinformatic parameter values have no significant effects on phylogenetic relationships. However, datasets maximizing resolution (MaxResol) produced considerably longer terminal branch lengths compared to datasets minimizing error rates (MinError), while relative internal branch lengths remained quite constant (**Figure 4**). This could be interpreted as an artefact resulting from the fact that each tip in a MaxResol tree has extra 'substitutions' per site due to sequencing errors. In agreement with this, recent evidence indicates that sequencing errors, if not corrected, can significantly influence branch length estimates (Kuhner and McGill, 2014). Other studies have suggested that two further factors may also bias branch length estimates: the assumption of a single evolutionary model and the presence of large amounts of missing data, whose effects may be more pronounced as dataset size and complexity increase (e.g. Lemmon et al., 2009: Schwartz and Mueller, 2010; Darriba et al., 2016). Despite the fact that our study design did not permit us to discriminate whether the misestimation of branch lengths was the result of any particular factor, it is clear that maximizing phylogenetic resolution leads to higher potential bias in branch length estimation than minimizing error rates, an issue that deserves further attention.

The comparison of inferred shifts in diversification rates between MaxResol and MinError datasets (after time-calibration) revealed significantly different patterns. In particular, the MaxResol configuration recovered no diversification rate shifts along the tree, while the MinError configuration resulted in three accelerations of diversification rates coinciding with the origin of the three largest taxonomical sections (**Figure 6**, **Figures S3 and S4**). Thus, the artificial inflation of terminal branch lengths caused by high SNP error rates may lead to spurious interpretations of evolutionary patterns in our particular study group and probably in other clades similarly subjected to rapid diversification. Radiating lineages may be particularly susceptible to the disruption of the detection of shifts in diversification rates when biases in estimates of terminal branch lengths occur, since these lineages are characterized by short branch lengths and low pairwise sequence divergence due to closely spaced branching events (Guzmán et al., 2009; Glor, 2010). Therefore, although the topological accuracy of phylogenetic trees is important for purposes such as taxonomic classification (e.g. see discussion in de Queiroz and Gauthier, 1990), it is essential to stress that the accuracy of tree branch lengths is critical for further evolutionary inferences such as divergence time estimation, diversification rate calculation, ancestral state reconstruction, tree-dependent comparative methods and biogeographic analyses (Lemmon et al., 2009; Darriba et al., 2016).

### Concatenation vs. Coalescent Approaches to GBS Phylogenetics

Researchers now routinely sequence hundreds to thousands of loci in non-model organisms using reduced-representation approaches in order to reconstruct their evolutionary histories (Giarla and Esselstyn, 2015). However, the analysis of these huge datasets involves trade-offs among computational efficiency, dataset size and simplifying assumptions (Giarla and Esselstyn, 2015) which sometimes force researchers to apply suboptimal inference methods (Kubatko and Degnan, 2007). Consequently, there is an ongoing debate among phylogeneticists as to which of the two approaches—i.e. concatenation vs. coalescent—is most appropriate for inferring phylogenies from phylogenomic datasets (Huang and Knowles, 2009; Lanier et al., 2014; Gatesy and Springer, 2014).

In our reconstructed phylogenetic trees, concatenation methods provided considerably higher phylogenetic resolution than coalescent methods for all parameter assemblies. However, they recovered high statistical support for alternative topologies resulting from a few incongruences, which mainly involved nodes in sects. *Pseudocistus* and *Helianthemum* (**Figure 2**). These results agree with previous studies in which concatenated analyses produced anomalously high statistical support for incorrect topologies when the two most commonly used branch support methods—i.e. bootstrap (BS) and posterior probability (PP)—are applied (e.g. Jones et al., 2013; Fernández-Mazuecos et al., 2018). Spurious relationships under concatenation methods may be the result of the "fenestrated" nature of the alignment when reduced-representation data are used (i.e. high proportion of missing data; Wiens and Morrill, 2011; Roure et al., 2013; Hinchliff and Roalson, 2013) and of systematic biases (Gadagkar et al., 2005; Kumar et al., 2011). Bias may result from the specification of a single substitution model, which assumes substitution rate homogeneity across the whole dataset. Partitioned analysis may prevent this problem, but it may be computationally problematic with high numbers of loci (Fernández-Mazuecos et al., 2018). The fact that the quartet sampling analyses displayed negative QC scores for some shallow nodes (**Figure 3**) shows that this alternative branch support metric reflects topology uncertainty more accurately and is able to distinguish among different causes of incongruence between datasets (Pease et al., 2018).

Alternatively, coalescent methods produce more congruent topologies than concatenation methods, but with a generally low BS within sects. *Pseudocistus* and *Helianthemum*. Although coalescent-based methods may better reflect topological uncertainty resulting from ILS and reticulate evolution in large datasets (Anderson et al., 2017), for our dataset these methods recovered limited resolution when error rates were minimized (**Figure S1**). This lack of resolution was particularly noticeable in the trees resulting from the NJst method, which are comparable with those reconstructed using Sanger sequences (Aparicio et al., 2017). Previous studies have suggested that the short length of GBS loci (c. 100–200 bp) may result in poorly informative gene trees, which may be problematic for species tree inference (Salichos and Rokas, 2013). Although these methods may be adequate at shallow evolutionary scales (e.g. to resolve phylogenetic relationship among closely related species and populations; Fernández-Mazuecos et al., 2018), they do not seem to be suitable for establishing a robust phylogenetic framework of species-rich clades, particularly under assembly configurations that minimize error rates. In fact, software packages focused on downstream macroevolutionary analyses usually require strictly bifurcating trees (e.g. BioGeoBEARS; Matzke, 2013) which have only been recovered under concatenation methods in our study case.

Based on the topological changes (particularly at shallow nodes) that we found associated with changes in assembly parameters (i.e. clustering threshold, minimum sample coverage and minimum taxon coverage), it is still clear that conducting multiple analyses based on a range of parameter values (Takahashi et al., 2014; Leaché et al., 2015), different phylogenetic approaches and a range of branch support methods is necessary to evaluate if high clade support values provide a realistic measurement of confidence (Fernández-Mazuecos et al., 2018; Pease et al., 2018).

### Systematics and Evolutionary Implications

#### Non-Monophyly of Taxa at Different Taxonomic Ranks

The robust phylogenetic reconstruction presented in this paper highlights the need for a comprehensive taxonomic review of the genus *Helianthemum*, from the definition of subgenera to the delimitation of species and subspecies. In particular, our study shows that the subgenus *Helianthemum* as currently defined is paraphyletic, since it is retrieved in two different non-sister clades (i.e. clades II and III). In addition, most taxonomically complex species (e.g. *H. apenninum*, *H. cinereum*, *H. marifolium*, *H. nummularium* and *H. oelandicum*), which are characterised by an array of morphological forms usually treated as subspecies (Soubani et al., 2014a, Soubani et al., 2014b; Volkova et al., 2016), are non-monophyletic (see **Figure 5**).

The topological conflicts detected for some nodes in the concatenation analyses (**Figure 2**)—particularly those involving the above-mentioned complex species—as well as the low support for the two large sects. *Pseudocistus* and *Helianthemum* in the QS and coalescent analyses (**Figures 3** and **S1**) likely reflect the fact that trait convergence, ILS, hybridization and introgression are currently playing an essential role in the differentiation of these lineages. This idea is also supported by phylogeographical approaches (Soubani et al., 2014a; Soubani et al., 2014b; Widén, 2015; Widén, 2018; Volkova et al., 2016). Future taxonomical and microevolutionary studies are therefore required to obtain more detailed insights into the processes driving species diversification and differentiation in these complex species (Martín-Hernanz et al., 2019).

#### Three Recent Radiating Lineages in Contrasting Geographical, Ecological and Temporal Contexts

In addition to a robust phylogenetic framework, the detection of recent evolutionary radiations requires the evaluation of the following operational criteria: 1) a recent common ancestor, 2) species-poor sister lineages, and 3) significant bursts of diversification (Nee et al., 1996; Sanderson and Donoghue, 1996; Pybus and Harvey, 2000; Schluter, 2000; Glor, 2010). Based on the first two criteria, the existence of three radiating lineages in *Helianthemum* was recently suggested by Aparicio et al. (2017). Here we provide further empirical evidence based on two analytical approaches that confirm the occurrence of significant bursts of diversification. Firstly, absolute net diversification rates calculated using the standardized method of Magallón and Sanderson (2001) reveal that diversification rates of the three largest sections of the genus *Helianthemum* (i.e. *Eriocarpum s.l*., *Pseudocistus* and *Helianthemum*) are similar to those of other radiating lineages in the Mediterranean Floristic Region including the white-flowered clade of *Cistus* and the western European clades of *Erysimum* and *Reseda* sect. *Phyteuma* (Vargas et al., 2018; **Table 4**). Secondly, we identified three significant increases in speciation rates at the base of the above-mentioned sections (**Figure 6**).

The occurrence of multiple radiations in a large clade represents a powerful comparative system for addressing fundamental questions about patterns and processes underlying rapid diversification, as has previously been demonstrated in other plant groups (e.g. *Echium*, García-Maroto et al., 2009; *Lupinus*, Drummond et al., 2012; *Androsace*, Roquet et al., 2013). Some clues can be derived from our analysis that can help to determine whether radiations in *Helianthemum* are adaptive or not: 1) homogeneous ecological conditions in sect. *Eriocarpum s.l.* (i.e. arid and semi-arid environments from Macaronesia, northern Africa, Horn of Africa, Anatolia, and central Asia; Aparicio et al., 2017) vs. heterogeneous in sects. *Pseudocistus* and *Helianthemum* (i.e. Mediterranean and alpine environments in Europe and western Asia; Aparicio et al., 2017); 2) Pliocene origin of sect. *Eriocarpum s.l.* vs. late Pliocene in sects. *Pseudocistus* and *Helianthemum*; and 3) constant speciation over time in sect. *Eriocarpum s.l.* vs. density-dependent cladogenesis in sects. *Pseudocistus* and *Helianthemum* (see **Figure 6**). Ongoing studies (Martín-Hernanz et al., unpublished) are specifically addressing the adaptative nature of trait evolution, biogeographic patterns and potential associations between diversification rate shifts and ancestral areas or character states on the basis of the robust phylogenetic framework here established.

## Data Availability Statement

SRA data can be found in NCBI using accession numbers in **Supplementary Table S4** or accessible with the following link (https://www.ncbi.nlm.nih.gov/sra/PRJNA573639). 

## Author Contributions

The idea and design of the research were developed by SM-H, AA, and RGA. The performance of the research was developed by SM-H, AA, ER, and RGA. The data collection was mainly carried out by SM-H, AA, ER, AR-B, AS-G, MO-C, and RGA. The analyses and interpretation of the data were carried out by SM-H, AA, MF-M, and RGA. Finally, the manuscript was written and discussed between all authors and led by SM-H, AA, and RGA.

## Conflict of Interest

The authors declare that the research was conducted in the absence of any commercial or financial relationships that could be construed as a potential conflict of interest.
