# Supplementary material for: Maximize Resolution or Minimize Error? Using Genotyping-By-Sequencing to Investigate the Recent Diversification of Helianthemum (Cistaceae)
Source: Front Plant Sci. 2019 Nov 11;10:1416. doi: 10.3389/fpls.2019.01416 (PMC6859804; doi:10.3389/fpls.2019.01416)
Supplement: Supplementary file 1 [file Presentation_1.pdf]

Supplementary Material

MAXIMIZE RESOLUTION OR MINIMIZE ERROR? USING GBS TO INVESTIGATE THE RECENT DIVERSIFICATION OF *HELIANTHEMUM* (CISTACEAE)

Sara Martín-Hernanz[1], Abelardo Aparicio[1], Mario Fernández-Mazuecos[2], Encarnación Rubio[1], J. Alfredo Reyes-Betancort[3], Arnoldo Santos-Guerra[3], María Olangua-Corral[4] & Rafael G. Albaladejo[1]*

[1] Departamento de Biología Vegetal y Ecología, Universidad de Sevilla, c/ Profesor García González 2, 41012 Sevilla, Spain; [2] Real Jardín Botánico (RJB-CSIC), Plaza de Murillo 2, 28014 Madrid, Spain; [3] Jardín de Aclimatación de la Orotava (ICIA), C/ Retama 2, 38400 Puerto de la Cruz, S/C de Tenerife, Spain; [4] Jardín Botánico Canario 'Viera y Clavijo' – Unidad Asociada CSIC (Cabildo de Gran Canaria), c/ Camino del Palmeral nº 15, Tafira Alta, 35017 Las Palmas de Gran Canaria, Spain.

*** Correspondence:**

Sara Martín-Hernanz

sara.martin.hernanz@gmail.com

The following Supporting Information is available for this article:

Figure S1 Phylogenetic trees of the genus *Helianthemum* resulting from applying maximum likelihood (RAxML), Bayesian inference (ExaBayes), quartet inference from SNPs (SVDquartets) and species tree estimation from unrooted gene trees (NJst) to MaxResol and MinError assemblies of GBS data under three minimum taxon coverage percentages (MinCov 15%, 25% and 50%).

Figure S2 Chronograms of the genus *Helianthemum* obtained in TreePL using MaxResol and MinError assemblies under three minimum taxon coverage percentages (MinCov 15%, 25% and 50%).

Figure S3 Diversification rate shift configurations of the genus *Helianthemum* with the highest posterior probabilities, as estimated by BAMM analyses of *Helianthemum* GBS phylogenetic trees.

Figure S4 Comparison of divergence times and diversification patterns of the genus *Helianthemum* recovered from the phylogenetic trees coming from of MaxResol and MinError configurations under 15% minimum taxon coverage.

Table S1 Sources of error and bias in phylogenomic inference associated with the use of GBS (Genotyping-by-sequencing) and RADseq (Restriction site- associated DNA sequencing) data.

Table S2 Sampling information of the studied taxa of the genus *Helianthemum*.

Table S3 Number of taxa (and species) per section of the genus *Helianthemum* included in this study.

Table S4 Sequence Read Archive (SRA) accession code and number of reads and loci per sample recovered in the sequencing and bioinformatics process.

Methods S1 Additional information about DNA extraction, library preparation and Next-generation sequencing.

Methods S2 Description of the bioinformatic parameters explored in this study.

Figure S1 Phylogenetic trees of the genus *Helianthemum* resulting from applying maximum likelihood (RAxML), Bayesian inference (ExaBayes), quartet inference from SNPs (SVDquartets) and species tree estimation from unrooted gene trees (NJst) to MaxResol and MinError assemblies of GBS data under three minimum taxon coverage percentages (MinCov 15%, 25% and 50%):

(a) RaxML tree from MaxResol configuration, MinCov 15% assembly
(b) ExaBayes tree from MaxResol configuration, MinCov 15% assembly
(c) SVDquarters tree from MaxResol configuration, MinCov 15% assembly
(d) NJst tree from MaxResol configuration, MinCov 15% assembly
(e) RaxML tree from MaxResol configuration, MinCov 25% assembly
(f) ExaBayes tree from MaxResol configuration, MinCov 25% assembly
(g) SVDquarters tree from MaxResol configuration, MinCov 25% assembly
(h) NJst tree from MaxResol configuration, MinCov 25% assembly
(i) RaxML tree from MaxResol configuration, MinCov 50% assembly
(j) ExaBayes tree from MaxResol configuration, MinCov 50% assembly
(k) SVDquarters tree from MaxResol configuration, MinCov 50% assembly
(l) NJst tree from MaxResol configuration, MinCov 50% assembly
(m) RaxML tree from MinError configuration, MinCov 15% assembly
(n) ExaBayes tree from MinError configuration, MinCov 15% assembly
(o) SVDquarters tree from MinError configuration, MinCov 15% assembly
(p) NJst tree from MinError configuration, MinCov 15% assembly
(q) RaxML tree from MinError configuration, MinCov 25% assembly
(r) ExaBayes tree from MinError configuration, MinCov 25% assembly
(s) SVDquarters tree from MinError configuration, MinCov 25% assembly
(t) NJst tree from MinError configuration, MinCov 25% assembly
(u) RaxML tree from MinError configuration, MinCov 50% assembly
(v) ExaBayes tree from MinError configuration, MinCov 50% assembly
(w) SVDquarters tree from MinError configuration, MinCov 50% assembly
(x) NJst tree from MinError configuration, MinCov 50% assembly

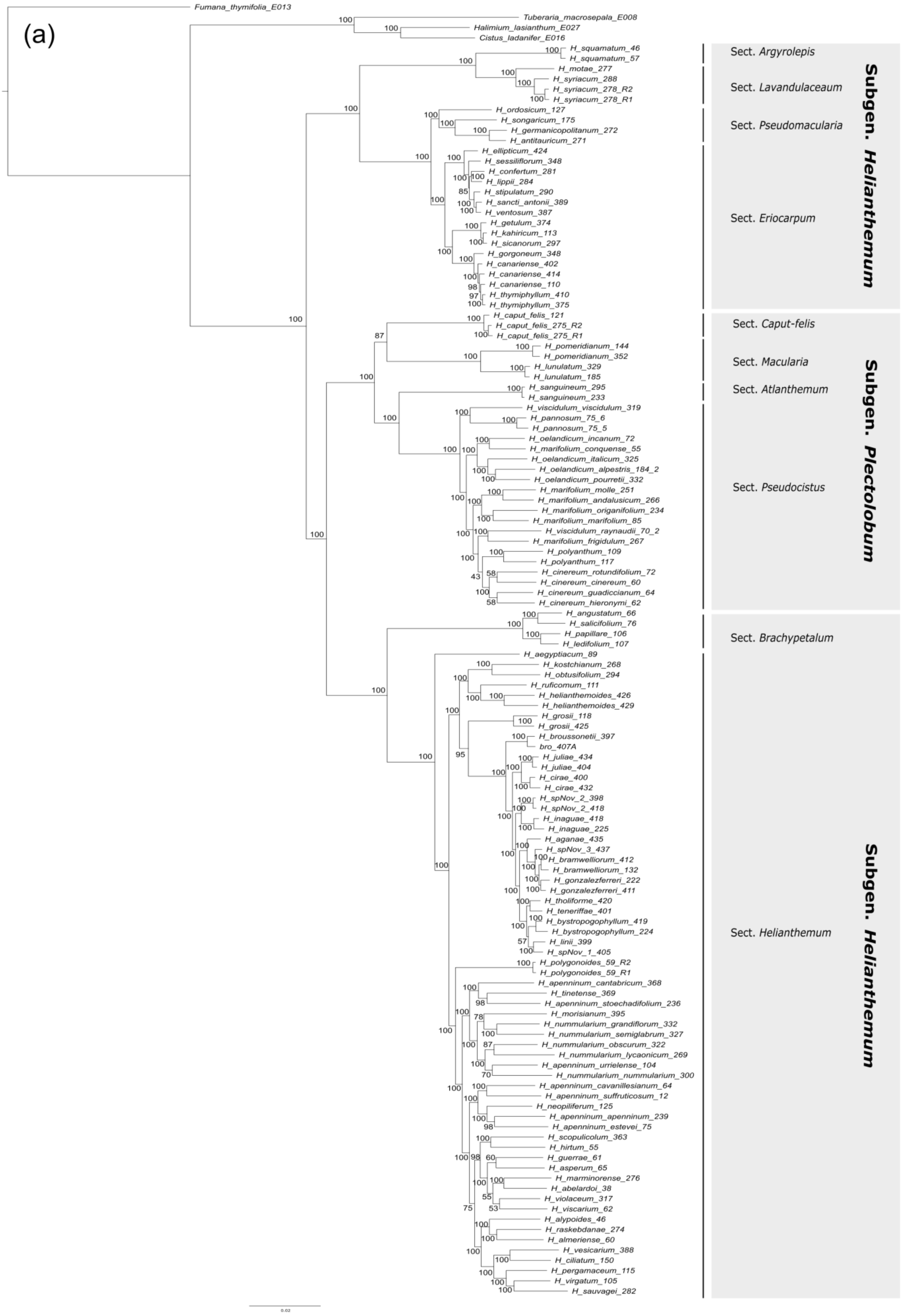
(a)
Fumana_thymifolia_E013
Tuberaria_macrosepala_E008
Halimium_lasianthum_E027
Cistus_ladanifer_E016
Sect. Argyrolepis
Sect. Lavandulaceaum
Sect. Pseudomacularia
Sect. Eriocarpum
Subgen. Helianthemum
Sect. Caput-felis
Sect. Macularia
Sect. Atlanthemum
Sect. Pseudocistus
Subgen. Plectolobum
Sect. Brachypetalum
Sect. Helianthemum
Subgen. Helianthemum
0.02

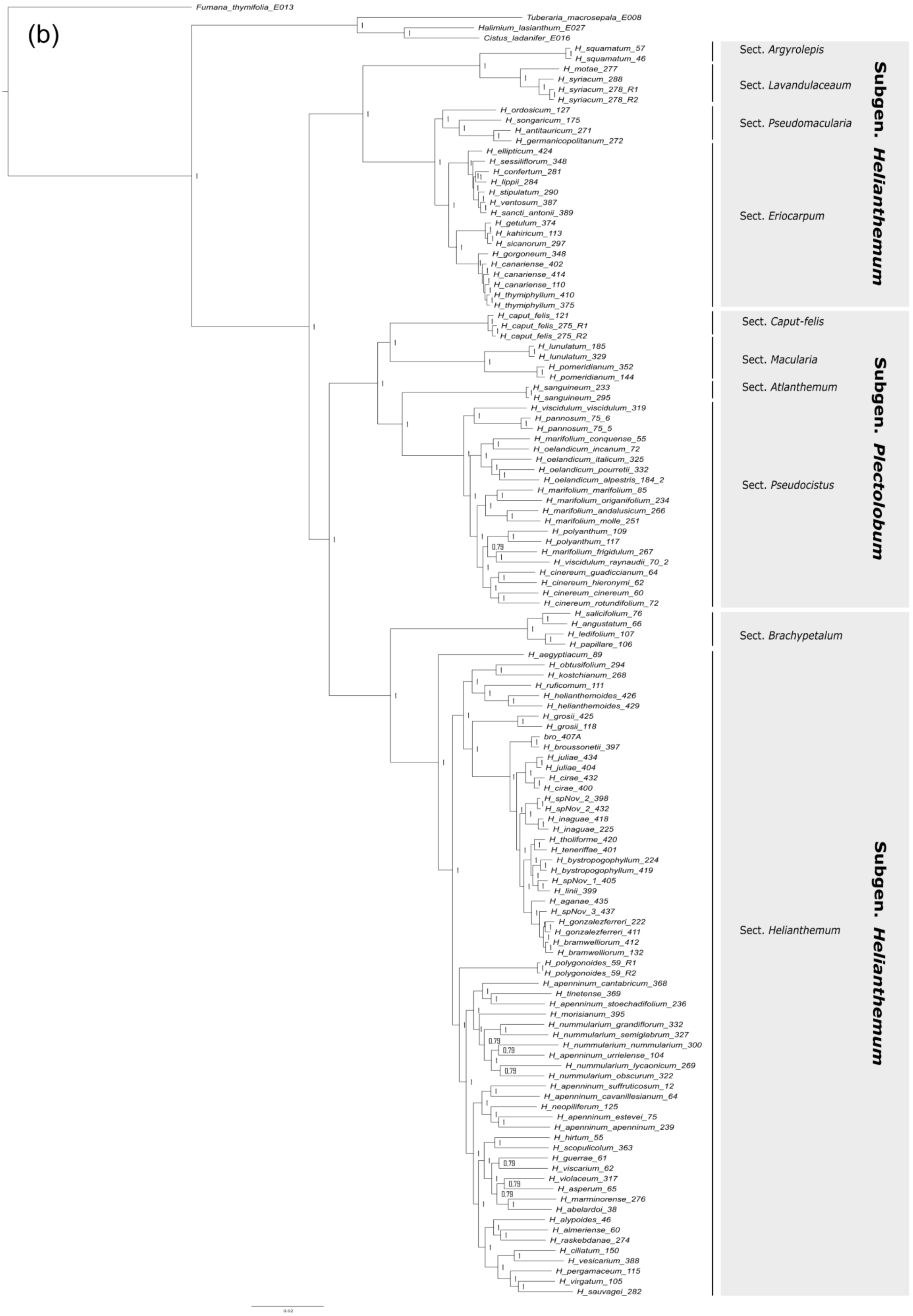
(b)
Fumana_thymifolia_E013
Tuberaria_macrosepala_E008
Halimium_lasianthum_E027
Cistus_ladanifer_E016
H_squamatum_57
H_squamatum_46
H_motae_277
H_syriacum_288
H_syriacum_278_R1
H_syriacum_278_R2
H_ordosicum_127
H_songaricum_175
H_antitauricum_271
H_germanicopolitanum_272
H_ellipticum_424
H_sessiliflorum_348
H_confertum_281
H_lippii_284
H_stipulatum_290
H_ventosum_387
H_sancti_antonii_389
H_getulum_374
H_kahiricum_113
H_sicanorum_297
H_gorgoneum_348
H_canariense_402
H_canariense_414
H_canariense_110
H_thymiphyllum_410
H_thymiphyllum_375
H_caput_felis_121
H_caput_felis_275_R1
H_caput_felis_275_R2
H_lunulatum_185
H_lunulatum_329
H_pomeridianum_352
H_pomeridianum_144
H_sanguineum_233
H_sanguineum_295
H_viscidulum_viscidulum_319
H_pannosum_75_6
H_pannosum_75_5
H_marifolium_conquense_55
H_oelandicum_incanum_72
H_oelandicum_italicum_325
H_oelandicum_pourretii_332
H_oelandicum_alpestris_184_2
H_marifolium_marifolium_85
H_marifolium_origanifolium_234
H_marifolium_andalusicum_266
H_marifolium_molle_251
H_polyanthum_109
H_polyanthum_117
H_marifolium_frigidulum_267
H_viscidulum_raynaudii_70_2
H_cinereum_guadiccianum_64
H_cinereum_hieronymi_62
H_cinereum_cinereum_60
H_cinereum_rotundifolium_72
H_salicifolium_76
H_angustatum_66
H_ledifolium_107
H_papillare_106
H_aegyptiacum_89
H_obtusifolium_294
H_kostchianum_268
H_ruficomum_111
H_helianthemoides_426
H_helianthemoides_429
H_grosii_425
H_grosii_118
bro_407A
H_broussonetii_397
H_juliae_434
H_juliae_404
H_cirae_432
H_cirae_400
H_spNov_2_398
H_spNov_2_432
H_inaguae_418
H_inaguae_225
H_tholiforme_420
H_teneriffae_401
H_bystropogophyllum_224
H_bystropogophyllum_419
H_spNov_1_405
H_linii_399
H_aganae_435
H_spNov_3_437
H_gonzalezferreri_222
H_gonzalezferreri_411
H_bramwelliorum_412
H_bramwelliorum_132
H_polygonoides_59_R1
H_polygonoides_59_R2
H_apenninum_cantabricum_368
H_tinetense_369
H_apenninum_stoechadifolium_236
H_morisianum_395
H_nummularium_grandiflorum_332
H_nummularium_semiglabrum_327
H_nummularium_nummularium_300
H_apenninum_urrielense_104
H_nummularium_lycaonicum_269
H_nummularium_obscurum_322
H_apenninum_suffruticosum_12
H_apenninum_cavanillesianum_64
H_neopiliferum_125
H_apenninum_estevei_75
H_apenninum_apenninum_239
H_hirtum_55
H_scopulicolum_363
H_guerrae_61
H_viscarium_62
H_violaceum_317
H_asperum_65
H_marminorense_276
H_abelardoi_38
H_alypoides_46
H_almeriense_60
H_raskebdanae_274
H_ciliatum_150
H_vesicarium_388
H_pergamaceum_115
H_virgatum_105
H_sauvagei_282
0.79
0.02
Sect. Argyrolepis
Sect. Lavandulaceaum
Sect. Pseudomacularia
Sect. Eriocarpum
Subgen. Helianthemum
Sect. Caput-felis
Sect. Macularia
Sect. Atlanthemum
Sect. Pseudocistus
Subgen. Plectolobum
Sect. Brachypetalum
Sect. Helianthemum
Subgen. Helianthemum

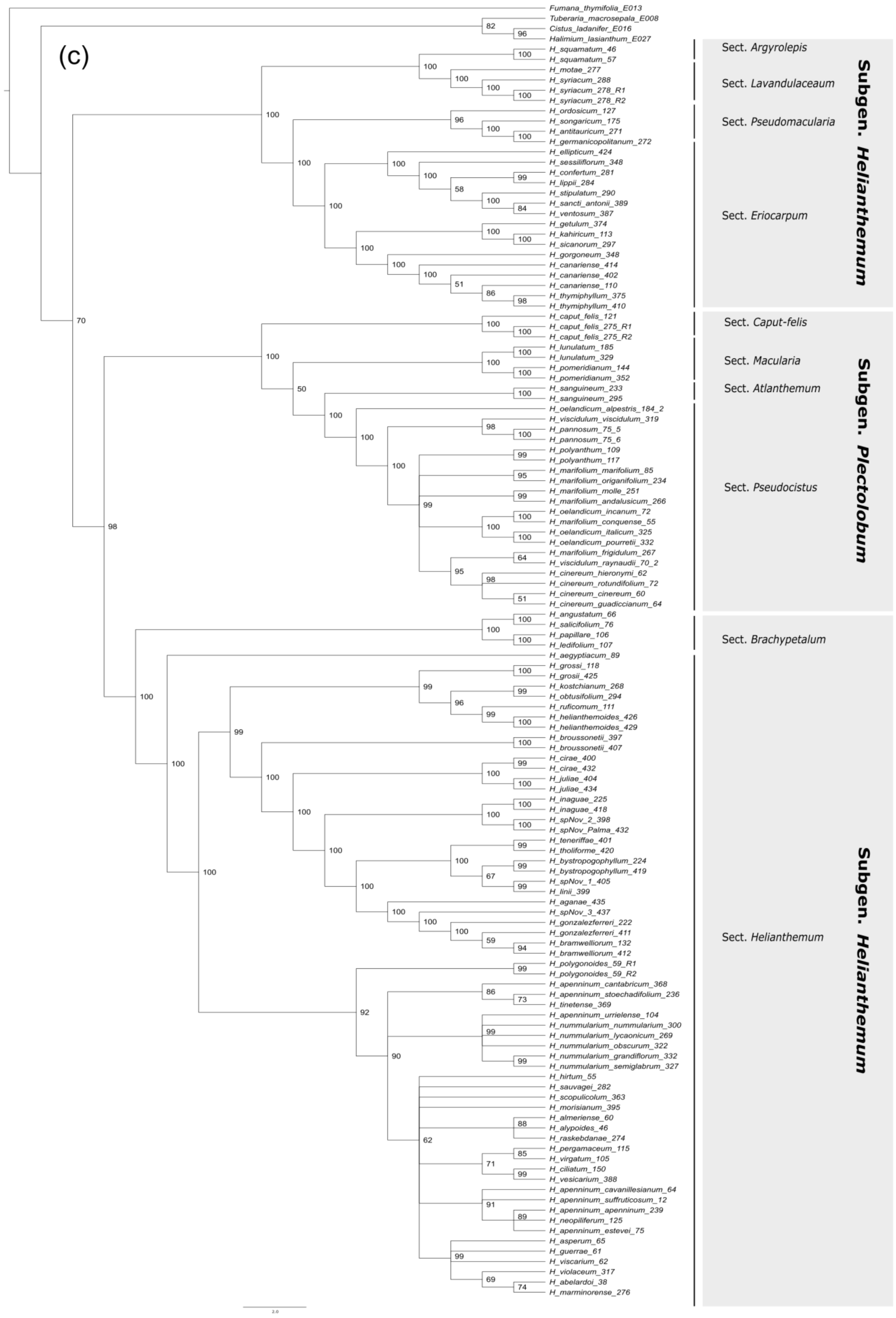
(c)
Fumana_thymifolia_E013
Tuberaria_macrosepala_E008
Cistus_ladanifer_E016
Halimium_lasianthum_E027
H_squamatum_46
H_squamatum_57
H_motae_277
H_syriacum_288
H_syriacum_278_R1
H_syriacum_278_R2
H_ordosicum_127
H_songaricum_175
H_antitauricum_271
H_germanicopolitanum_272
H_ellipticum_424
H_sessiliflorum_348
H_confertum_281
H_lippii_284
H_stipulatum_290
H_sancti_antonii_389
H_ventosum_387
H_getulum_374
H_kahiricum_113
H_sicanorum_297
H_gorgoneum_348
H_canariense_414
H_canariense_402
H_canariense_110
H_thymiphyllum_375
H_thymiphyllum_410
H_caput_felis_121
H_caput_felis_275_R1
H_caput_felis_275_R2
H_lunulatum_185
H_lunulatum_329
H_pomeridianum_144
H_pomeridianum_352
H_sanguineum_233
H_sanguineum_295
H_oelandicum_alpestris_184_2
H_viscidulum_viscidulum_319
H_pannosum_75_5
H_pannosum_75_6
H_polyanthum_109
H_polyanthum_117
H_marifolium_marifolium_85
H_marifolium_origanifolium_234
H_marifolium_molle_251
H_marifolium_andalusicum_266
H_oelandicum_incanum_72
H_marifolium_conquense_55
H_oelandicum_italicum_325
H_oelandicum_pourretii_332
H_marifolium_frigidulum_267
H_viscidulum_raynaudii_70_2
H_cinereum_hieronymi_62
H_cinereum_rotundifolium_72
H_cinereum_cinereum_60
H_cinereum_guadiccianum_64
H_angustatum_66
H_salicifolium_76
H_papillare_106
H_ledifolium_107
H_aegyptiacum_89
H_grossi_118
H_grosii_425
H_kostchianum_268
H_obtusifolium_294
H_ruficomum_111
H_helianthemoides_426
H_helianthemoides_429
H_broussonetii_397
H_broussonetii_407
H_cirae_400
H_cirae_432
H_juliae_404
H_juliae_434
H_inaguae_225
H_inaguae_418
H_spNov_2_398
H_spNov_Palma_432
H_teneriffae_401
H_tholiforme_420
H_bystropogophyllum_224
H_bystropogophyllum_419
H_spNov_1_405
H_linii_399
H_aganae_435
H_spNov_3_437
H_gonzalezferreri_222
H_gonzalezferreri_411
H_bramwelliorum_132
H_bramwelliorum_412
H_polygonoides_59_R1
H_polygonoides_59_R2
H_apenninum_cantabricum_368
H_apenninum_stoechadifolium_236
H_tinetense_369
H_apenninum_urrielense_104
H_nummularium_nummularium_300
H_nummularium_lycaonicum_269
H_nummularium_obscurum_322
H_nummularium_grandiflorum_332
H_nummularium_semiglabrum_327
H_hirtum_55
H_sauvagei_282
H_scopulicolum_363
H_morisianum_395
H_almeriense_60
H_alypoides_46
H_raskebdanae_274
H_pergamaceum_115
H_virgatum_105
H_ciliatum_150
H_vesicarium_388
H_apenninum_cavanillesianum_64
H_apenninum_suffruticosum_12
H_apenninum_apenninum_239
H_neopiliferum_125
H_apenninum_estevei_75
H_asperum_65
H_guerrae_61
H_viscarium_62
H_violaceum_317
H_abelardoi_38
H_marminorense_276
Sect. Argyrolepis
Sect. Lavandulaceaum
Sect. Pseudomacularia
Sect. Eriocarpum
Subgen. Helianthemum
Sect. Caput-felis
Sect. Macularia
Sect. Atlanthemum
Sect. Pseudocistus
Subgen. Plectolobum
Sect. Brachypetalum
Sect. Helianthemum
Subgen. Helianthemum

(d)

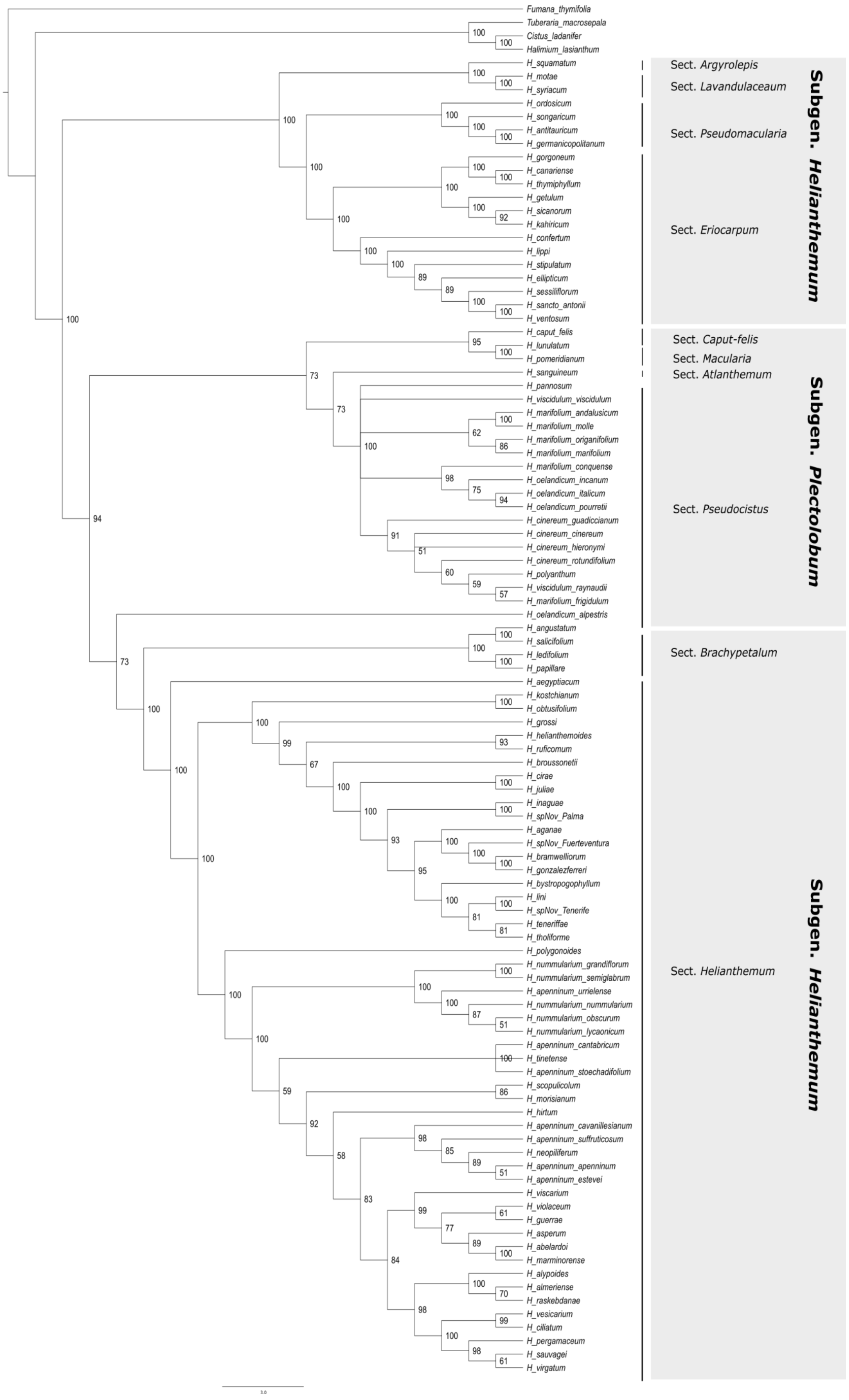

Fumana_thymifolia
Tuberaria_macrosepala
Cistus_ladanifer
Halimium_lasianthum
H_squamatum
H_motae
H_syriacum
H_ordosicum
H_songaricum
H_antitauricum
H_germanicopolitanum
H_gorgoneum
H_canariense
H_thymiphyllum
H_getulum
H_sicanorum
H_kahiricum
H_confertum
H_lippi
H_stipulatum
H_ellipticum
H_sessiliflorum
H_sancto_antonii
H_ventosum
H_caput_felis
H_lunulatum
H_pomeridianum
H_sanguineum
H_pannosum
H_viscidulum_viscidulum
H_marifolium_andalusicum
H_marifolium_molle
H_marifolium_origanifolium
H_marifolium_marifolium
H_marifolium_conquense
H_oelandicum_incanum
H_oelandicum_italicum
H_oelandicum_pourretii
H_cinereum_guadiccianum
H_cinereum_cinereum
H_cinereum_hieronymi
H_cinereum_rotundifolium
H_polyanthum
H_viscidulum_raynaudii
H_marifolium_frigidulum
H_oelandicum_alpestris
H_angustatum
H_salicifolium
H_ledifolium
H_papillare
H_aegyptiacum
H_kostchianum
H_obtusifolium
H_grossi
H_helianthemoides
H_ruficomum
H_broussonetii
H_cirae
H_juliae
H_inaguae
H_spNov_Palma
H_aganae
H_spNov_Fuerteventura
H_bramwelliorum
H_gonzalezferreri
H_bystropogophyllum
H_lini
H_spNov_Tenerife
H_teneriffae
H_tholiforme
H_polygonoides
H_nummularium_grandiflorum
H_nummularium_semiglabrum
H_apenninum_urrielense
H_nummularium_nummularium
H_nummularium_obscurum
H_nummularium_lycaonicum
H_apenninum_cantabricum
H_tinetense
H_apenninum_stoechadifolium
H_scopulicolum
H_morisianum
H_hirtum
H_apenninum_cavanillesianum
H_apenninum_suffruticosum
H_neopiliferum
H_apenninum_apenninum
H_apenninum_estevei
H_viscarium
H_violaceum
H_guerrae
H_asperum
H_abelardoi
H_marminorense
H_alypoides
H_almeriense
H_raskebdanae
H_vesicarium
H_ciliatum
H_pergamaceum
H_sauvagei
H_virgatum
3.0
Sect. Argyrolepis
Sect. Lavandulaceaum
Sect. Pseudomacularia
Sect. Eriocarpum
Subgen. Helianthemum
Sect. Caput-felis
Sect. Macularia
Sect. Atlanthemum
Sect. Pseudocistus
Subgen. Plectolobum
Sect. Brachypetalum
Sect. Helianthemum
Subgen. Helianthemum

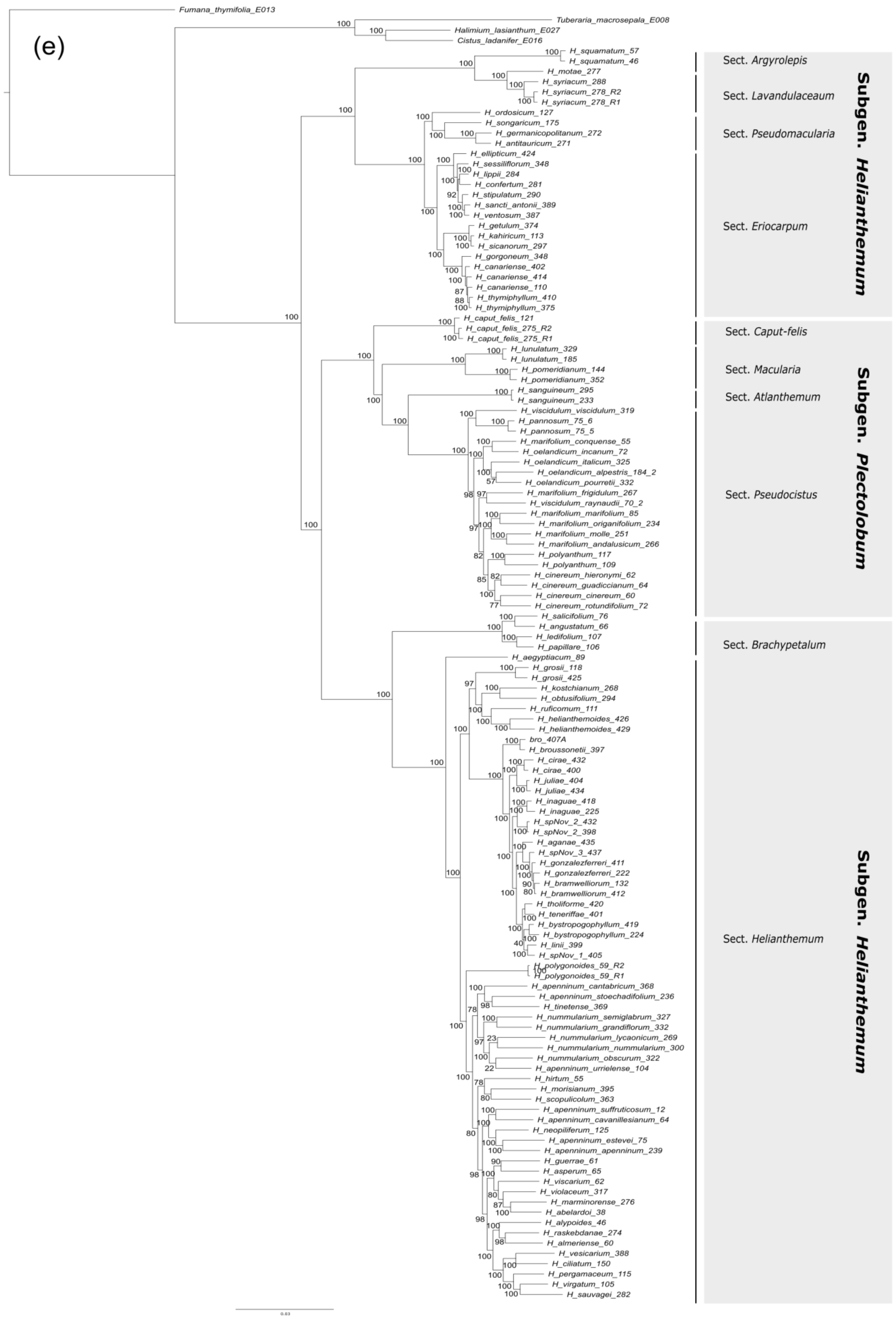

(e)
Fumana_thymifolia_E013
Tuberaria_macrosepala_E008
Halimium_lasianthum_E027
Cistus_ladanifer_E016
H_squamatum_57
H_squamatum_46
H_motae_277
H_syriacum_288
H_syriacum_278_R2
H_syriacum_278_R1
H_ordosicum_127
H_songaricum_175
H_germanicopolitanum_272
H_antitauricum_271
H_ellipticum_424
H_sessiliflorum_348
H_lippii_284
H_confertum_281
H_stipulatum_290
H_sancti_antonii_389
H_ventosum_387
H_getulum_374
H_kahiricum_113
H_sicanorum_297
H_gorgoneum_348
H_canariense_402
H_canariense_414
H_canariense_110
H_thymiphyllum_410
H_thymiphyllum_375
H_caput_felis_121
H_caput_felis_275_R2
H_caput_felis_275_R1
H_lunulatum_329
H_lunulatum_185
H_pomeridianum_144
H_pomeridianum_352
H_sanguineum_295
H_sanguineum_233
H_viscidulum_viscidulum_319
H_pannosum_75_6
H_pannosum_75_5
H_marifolium_conquense_55
H_oelandicum_incanum_72
H_oelandicum_italicum_325
H_oelandicum_alpestris_184_2
H_oelandicum_pourretii_332
H_marifolium_frigidulum_267
H_viscidulum_raynaudii_70_2
H_marifolium_marifolium_85
H_marifolium_origanifolium_234
H_marifolium_molle_251
H_marifolium_andalusicum_266
H_polyanthum_117
H_polyanthum_109
H_cinereum_hieronymi_62
H_cinereum_guadiccianum_64
H_cinereum_cinereum_60
H_cinereum_rotundifolium_72
H_salicifolium_76
H_angustatum_66
H_ledifolium_107
H_papillare_106
H_aegyptiacum_89
H_grosii_118
H_grosii_425
H_kostchianum_268
H_obtusifolium_294
H_ruficomum_111
H_helianthemoides_426
H_helianthemoides_429
bro_407A
H_broussonetii_397
H_cirae_432
H_cirae_400
H_juliae_404
H_juliae_434
H_inaguae_418
H_inaguae_225
H_spNov_2_432
H_spNov_2_398
H_aganae_435
H_spNov_3_437
H_gonzalezferreri_411
H_gonzalezferreri_222
H_bramwelliorum_132
H_bramwelliorum_412
H_tholiforme_420
H_teneriffae_401
H_bystropogophyllum_419
H_bystropogophyllum_224
H_linii_399
H_spNov_1_405
H_polygonoides_59_R2
H_polygonoides_59_R1
H_apenninum_cantabricum_368
H_apenninum_stoechadifolium_236
H_tinetense_369
H_nummularium_semiglabrum_327
H_nummularium_grandiflorum_332
H_nummularium_lycaonicum_269
H_nummularium_nummularium_300
H_nummularium_obscurum_322
H_apenninum_urrielense_104
H_hirtum_55
H_morisianum_395
H_scopulicolum_363
H_apenninum_suffruticosum_12
H_apenninum_cavanillesianum_64
H_neopiliferum_125
H_apenninum_estevei_75
H_apenninum_apenninum_239
H_guerrae_61
H_asperum_65
H_viscarium_62
H_violaceum_317
H_marminorense_276
H_abelardoi_38
H_alypoides_46
H_raskebdanae_274
H_almeriense_60
H_vesicarium_388
H_ciliatum_150
H_pergamaceum_115
H_virgatum_105
H_sauvagei_282
0.03
Sect. Argyrolepis
Sect. Lavandulaceaum
Sect. Pseudomacularia
Sect. Eriocarpum
Subgen. Helianthemum
Sect. Caput-felis
Sect. Macularia
Sect. Atlanthemum
Sect. Pseudocistus
Subgen. Plectolobum
Sect. Brachypetalum
Sect. Helianthemum
Subgen. Helianthemum

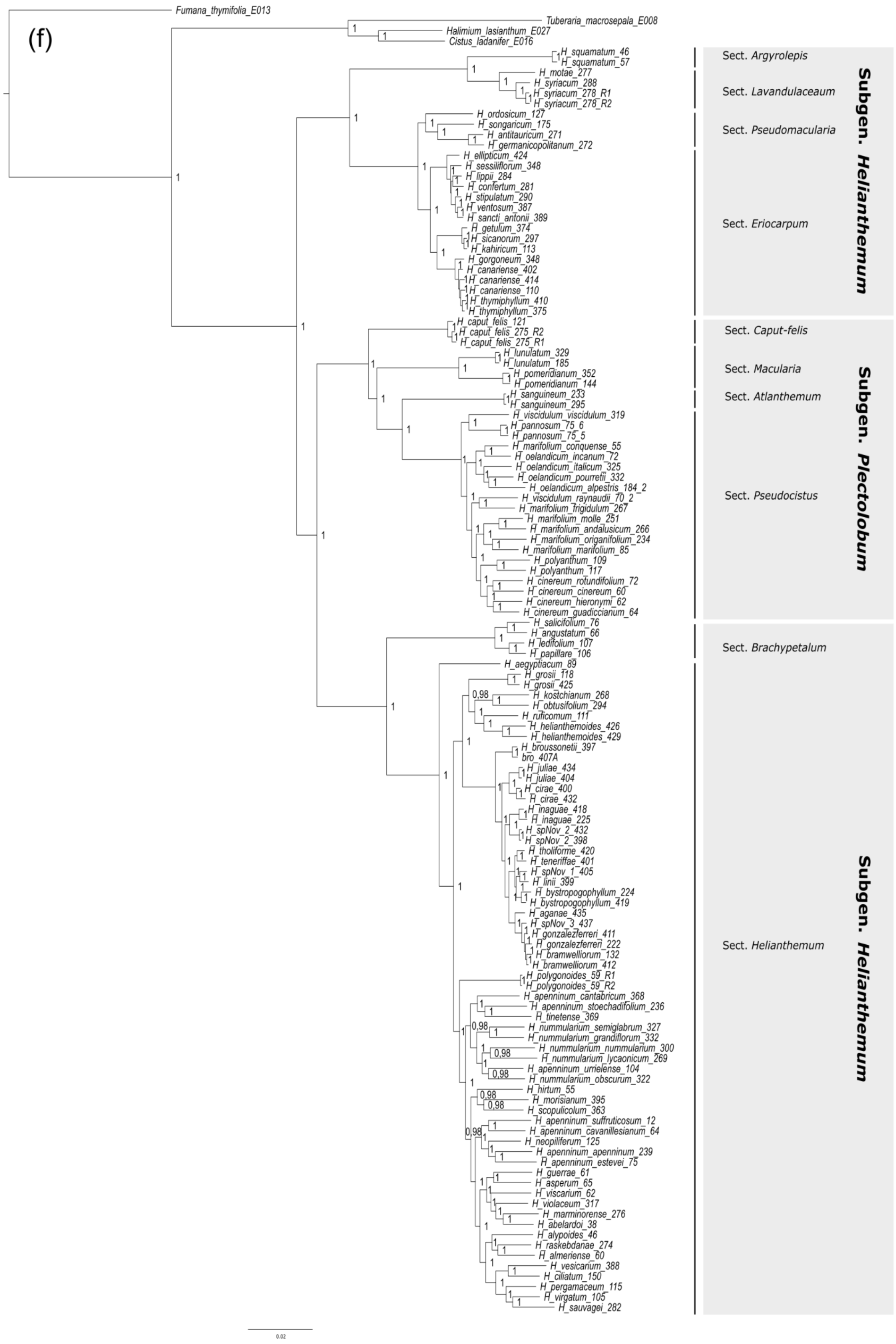

(f)
Fumana_thymifolia_E013
Tuberaria_macrosepala_E008
Halimium_lasianthum_E027
Cistus_ladanifer_E016
H_squamatum_46
H_squamatum_57
H_motae_277
H_syriacum_288
H_syriacum_278_R1
H_syriacum_278_R2
H_ordosicum_127
H_songaricum_175
H_antitauricum_271
H_germanicopolitanum_272
H_ellipticum_424
H_sessiliflorum_348
H_lippii_284
H_confertum_281
H_stipulatum_290
H_ventosum_387
H_sancti_antonii_389
H_getulum_374
H_sicanorum_297
H_kahiricum_113
H_gorgoneum_348
H_canariense_402
H_canariense_414
H_canariense_110
H_thymiphyllum_410
H_thymiphyllum_375
H_caput_felis_121
H_caput_felis_275_R2
H_caput_felis_275_R1
H_lunulatum_329
H_lunulatum_185
H_pomeridianum_352
H_pomeridianum_144
H_sanguineum_233
H_sanguineum_295
H_viscidulum_viscidulum_319
H_pannosum_75_6
H_pannosum_75_5
H_marifolium_conquense_55
H_oelandicum_incanum_72
H_oelandicum_italicum_325
H_oelandicum_pourretii_332
H_oelandicum_alpestris_184_2
H_viscidulum_raynaudii_70_2
H_marifolium_frigidulum_267
H_marifolium_molle_251
H_marifolium_andalusicum_266
H_marifolium_origanifolium_234
H_marifolium_marifolium_85
H_polyanthum_109
H_polyanthum_117
H_cinereum_rotundifolium_72
H_cinereum_cinereum_60
H_cinereum_hieronymi_62
H_cinereum_guadiccianum_64
H_salicifolium_76
H_angustatum_66
H_ledifolium_107
H_papillare_106
H_aegyptiacum_89
H_grosii_118
H_grosii_425
H_kostchianum_268
H_obtusifolium_294
H_ruficomum_111
H_helianthemoides_426
H_helianthemoides_429
H_broussonetii_397
bro_407A
H_juliae_434
H_juliae_404
H_cirae_400
H_cirae_432
H_inaguae_418
H_inaguae_225
H_spNov_2_432
H_spNov_2_398
H_tholiforme_420
H_teneriffae_401
H_spNov_1_405
H_linii_399
H_bystropogophyllum_224
H_bystropogophyllum_419
H_aganae_435
H_spNov_3_437
H_gonzalezferreri_411
H_gonzalezferreri_222
H_bramwelliorum_132
H_bramwelliorum_412
H_polygonoides_59_R1
H_polygonoides_59_R2
H_apenninum_cantabricum_368
H_apenninum_stoechadifolium_236
H_tinetense_369
H_nummularium_semiglabrum_327
H_nummularium_grandiflorum_332
H_nummularium_nummularium_300
H_nummularium_lycaonicum_269
H_apenninum_urrielense_104
H_nummularium_obscurum_322
H_hirtum_55
H_morisianum_395
H_scopulicolum_363
H_apenninum_suffruticosum_12
H_apenninum_cavanillesianum_64
H_neopiliferum_125
H_apenninum_apenninum_239
H_apenninum_estevei_75
H_guerrae_61
H_asperum_65
H_viscarium_62
H_violaceum_317
H_marminorense_276
H_abelardoi_38
H_alypoides_46
H_raskebdanae_274
H_almeriense_60
H_vesicarium_388
H_ciliatum_150
H_pergamaceum_115
H_virgatum_105
H_sauvagei_282
Sect. Argyrolepis
Sect. Lavandulaceaum
Sect. Pseudomacularia
Sect. Eriocarpum
Subgen. Helianthemum
Sect. Caput-felis
Sect. Macularia
Sect. Atlanthemum
Sect. Pseudocistus
Subgen. Plectolobum
Sect. Brachypetalum
Sect. Helianthemum
Subgen. Helianthemum
0.02

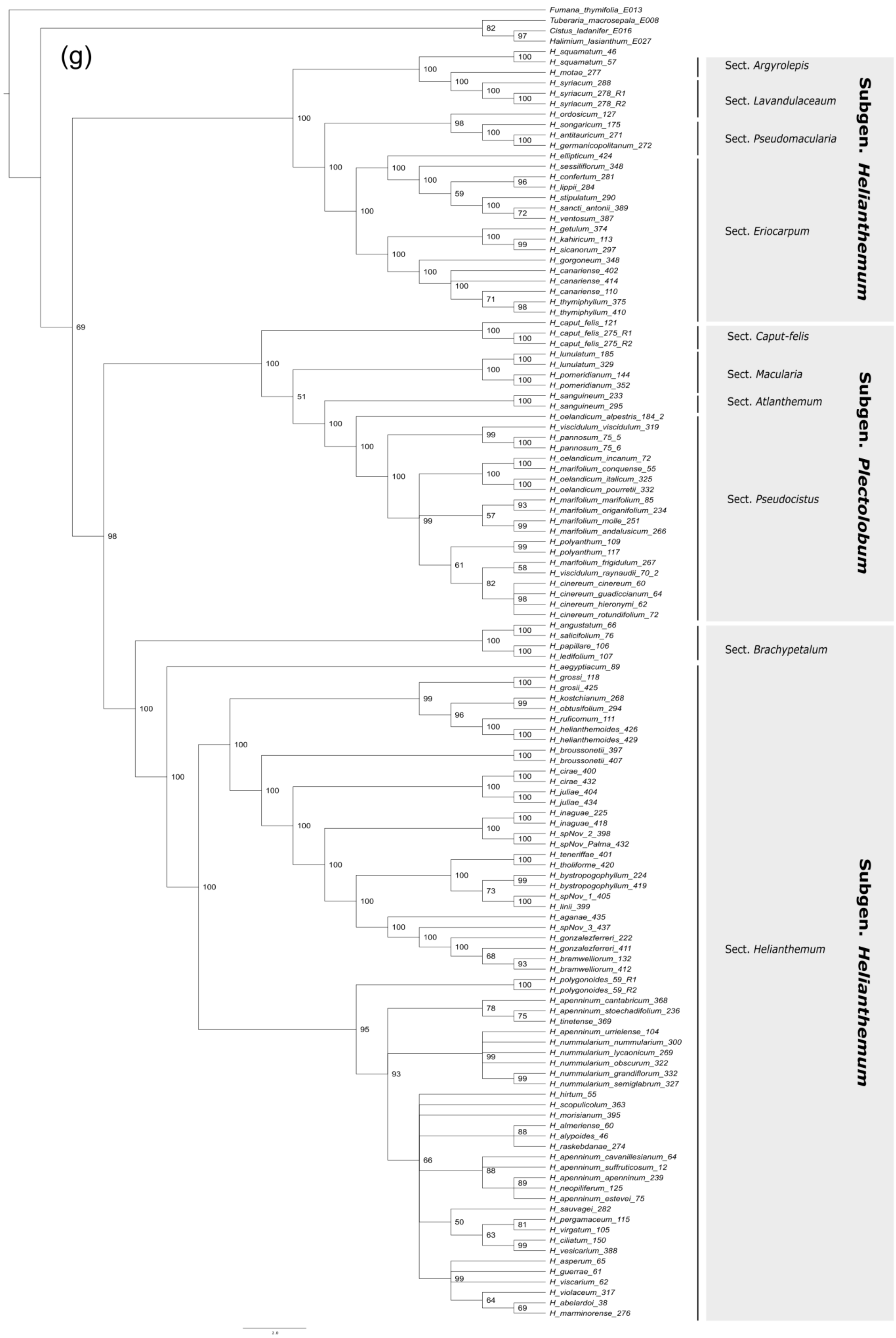
(g)
Fumana_thymifolia_E013
Tuberaria_macrosepala_E008
Cistus_ladanifer_E016
Halimium_lasianthum_E027
H_squamatum_46
H_squamatum_57
H_motae_277
H_syriacum_288
H_syriacum_278_R1
H_syriacum_278_R2
H_ordosicum_127
H_songaricum_175
H_antitauricum_271
H_germanicopolitanum_272
H_ellipticum_424
H_sessiliflorum_348
H_confertum_281
H_lippii_284
H_stipulatum_290
H_sancti_antonii_389
H_ventosum_387
H_getulum_374
H_kahiricum_113
H_sicanorum_297
H_gorgoneum_348
H_canariense_402
H_canariense_414
H_canariense_110
H_thymiphyllum_375
H_thymiphyllum_410
H_caput_felis_121
H_caput_felis_275_R1
H_caput_felis_275_R2
H_lunulatum_185
H_lunulatum_329
H_pomeridianum_144
H_pomeridianum_352
H_sanguineum_233
H_sanguineum_295
H_oelandicum_alpestris_184_2
H_viscidulum_viscidulum_319
H_pannosum_75_5
H_pannosum_75_6
H_oelandicum_incanum_72
H_marifolium_conquense_55
H_oelandicum_italicum_325
H_oelandicum_pourretii_332
H_marifolium_marifolium_85
H_marifolium_origanifolium_234
H_marifolium_molle_251
H_marifolium_andalusicum_266
H_polyanthum_109
H_polyanthum_117
H_marifolium_frigidulum_267
H_viscidulum_raynaudii_70_2
H_cinereum_cinereum_60
H_cinereum_guadiccianum_64
H_cinereum_hieronymi_62
H_cinereum_rotundifolium_72
H_angustatum_66
H_salicifolium_76
H_papillare_106
H_ledifolium_107
H_aegyptiacum_89
H_grossi_118
H_grosii_425
H_kostchianum_268
H_obtusifolium_294
H_ruficomum_111
H_helianthemoides_426
H_helianthemoides_429
H_broussonetii_397
H_broussonetii_407
H_cirae_400
H_cirae_432
H_juliae_404
H_juliae_434
H_inaguae_225
H_inaguae_418
H_spNov_2_398
H_spNov_Palma_432
H_teneriffae_401
H_tholiforme_420
H_bystropogophyllum_224
H_bystropogophyllum_419
H_spNov_1_405
H_linii_399
H_aganae_435
H_spNov_3_437
H_gonzalezferreri_222
H_gonzalezferreri_411
H_bramwelliorum_132
H_bramwelliorum_412
H_polygonoides_59_R1
H_polygonoides_59_R2
H_apenninum_cantabricum_368
H_apenninum_stoechadifolium_236
H_tinetense_369
H_apenninum_urrielense_104
H_nummularium_nummularium_300
H_nummularium_lycaonicum_269
H_nummularium_obscurum_322
H_nummularium_grandiflorum_332
H_nummularium_semiglabrum_327
H_hirtum_55
H_scopulicolum_363
H_morisianum_395
H_almeriense_60
H_alypoides_46
H_raskebdanae_274
H_apenninum_cavanillesianum_64
H_apenninum_suffruticosum_12
H_apenninum_apenninum_239
H_neopiliferum_125
H_apenninum_estevei_75
H_sauvagei_282
H_pergamaceum_115
H_virgatum_105
H_ciliatum_150
H_vesicarium_388
H_asperum_65
H_guerrae_61
H_viscarium_62
H_violaceum_317
H_abelardoi_38
H_marminorense_276
Sect. Argyrolepis
Sect. Lavandulaceaum
Sect. Pseudomacularia
Sect. Eriocarpum
Subgen. Helianthemum
Sect. Caput-felis
Sect. Macularia
Sect. Atlanthemum
Sect. Pseudocistus
Subgen. Plectolobum
Sect. Brachypetalum
Sect. Helianthemum
Subgen. Helianthemum
2.0

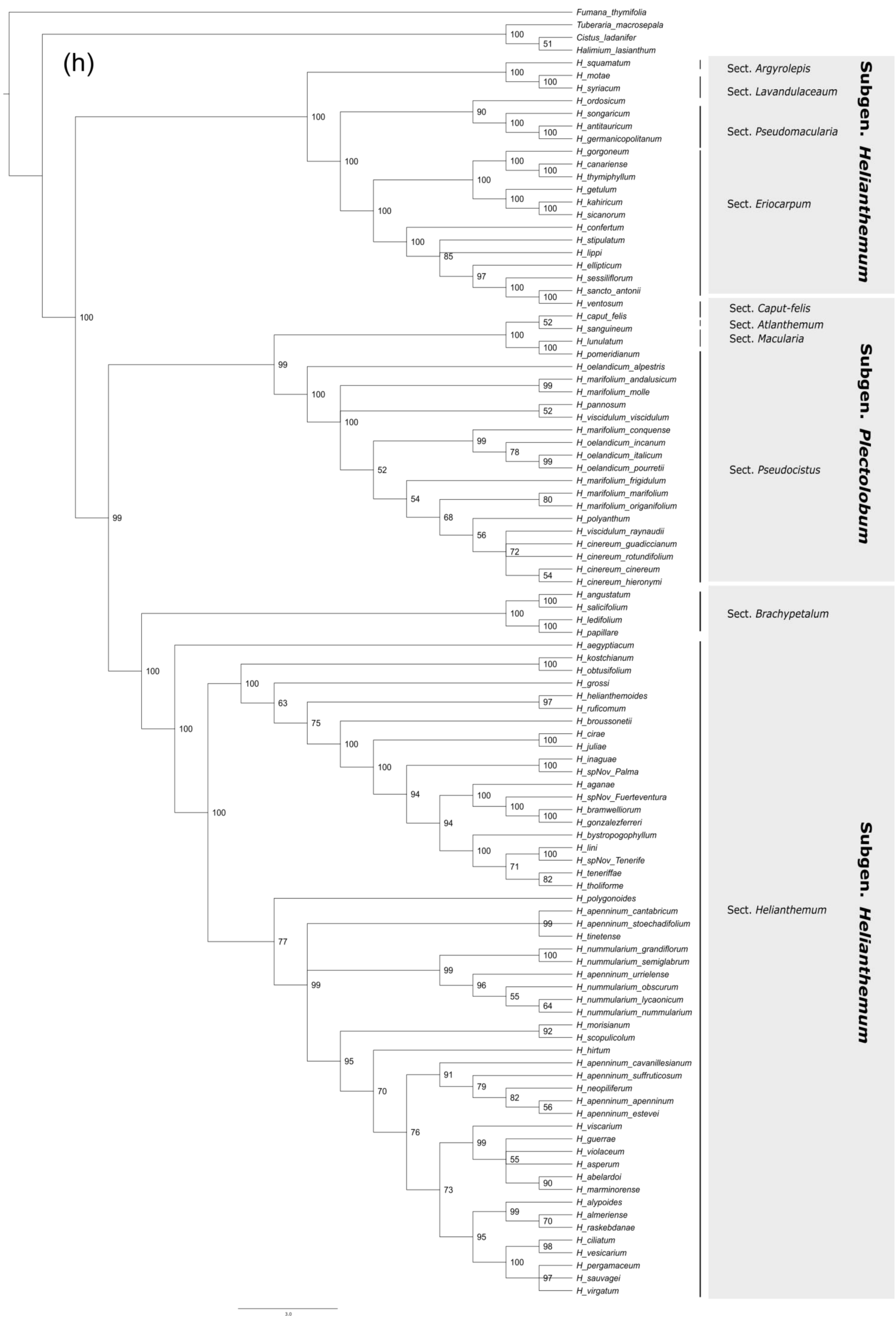
(h)
Fumana_thymifolia
Tuberaria_macrosepala
Cistus_ladanifer
Halimium_lasianthum
H_squamatum
H_motae
H_syriacum
H_ordosicum
H_songaricum
H_antitauricum
H_germanicopolitanum
H_gorgoneum
H_canariense
H_thymiphyllum
H_getulum
H_kahiricum
H_sicanorum
H_confertum
H_stipulatum
H_lippi
H_ellipticum
H_sessiliflorum
H_sancto_antonii
H_ventosum
H_caput_felis
H_sanguineum
H_lunulatum
H_pomeridianum
H_oelandicum_alpestris
H_marifolium_andalusicum
H_marifolium_molle
H_pannosum
H_viscidulum_viscidulum
H_marifolium_conquense
H_oelandicum_incanum
H_oelandicum_italicum
H_oelandicum_pourretii
H_marifolium_frigidulum
H_marifolium_marifolium
H_marifolium_origanifolium
H_polyanthum
H_viscidulum_raynaudii
H_cinereum_guadiccianum
H_cinereum_rotundifolium
H_cinereum_cinereum
H_cinereum_hieronymi
H_angustatum
H_salicifolium
H_ledifolium
H_papillare
H_aegyptiacum
H_kostchianum
H_obtusifolium
H_grossi
H_helianthemoides
H_ruficomum
H_broussonetii
H_cirae
H_juliae
H_inaguae
H_spNov_Palma
H_aganae
H_spNov_Fuerteventura
H_bramwelliorum
H_gonzalezferreri
H_bystropogophyllum
H_lini
H_spNov_Tenerife
H_teneriffae
H_tholiforme
H_polygonoides
H_apenninum_cantabricum
H_apenninum_stoechadifolium
H_tinetense
H_nummularium_grandiflorum
H_nummularium_semiglabrum
H_apenninum_urrielense
H_nummularium_obscurum
H_nummularium_lycaonicum
H_nummularium_nummularium
H_morisianum
H_scopulicolum
H_hirtum
H_apenninum_cavanillesianum
H_apenninum_suffruticosum
H_neopiliferum
H_apenninum_apenninum
H_apenninum_estevei
H_viscarium
H_guerrae
H_violaceum
H_asperum
H_abelardoi
H_marminorense
H_alypoides
H_almeriense
H_raskebdanae
H_ciliatum
H_vesicarium
H_pergamaceum
H_sauvagei
H_virgatum
Sect. Argyrolepis
Sect. Lavandulaceaum
Sect. Pseudomacularia
Sect. Eriocarpum
Subgen. Helianthemum
Sect. Caput-felis
Sect. Atlanthemum
Sect. Macularia
Sect. Pseudocistus
Subgen. Plectolobum
Sect. Brachypetalum
Sect. Helianthemum
Subgen. Helianthemum

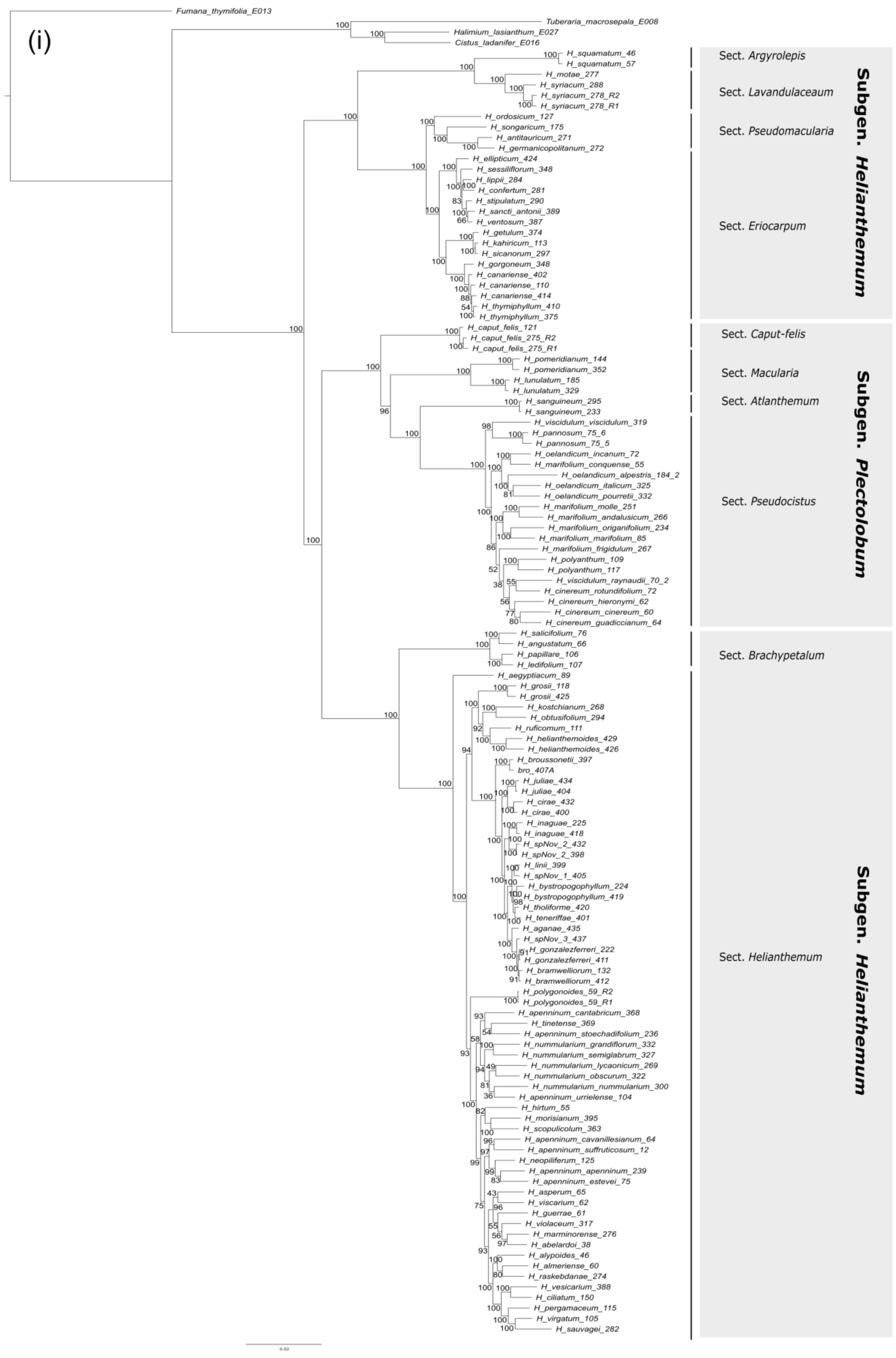

(i)
Fumana_thymifolia_E013
Tuberaria_macrosepala_E008
Halimium_lasianthum_E027
Cistus_ladanifer_E016
H_squamatum_46
H_squamatum_57
H_motae_277
H_syriacum_288
H_syriacum_278_R2
H_syriacum_278_R1
H_ordosicum_127
H_songaricum_175
H_antitauricum_271
H_germanicopolitanum_272
H_ellipticum_424
H_sessiliflorum_348
H_lippii_284
H_confertum_281
H_stipulatum_290
H_sancti_antonii_389
H_ventosum_387
H_getulum_374
H_kahiricum_113
H_sicanorum_297
H_gorgoneum_348
H_canariense_402
H_canariense_110
H_canariense_414
H_thymiphyllum_410
H_thymiphyllum_375
H_caput_felis_121
H_caput_felis_275_R2
H_caput_felis_275_R1
H_pomeridianum_144
H_pomeridianum_352
H_lunulatum_185
H_lunulatum_329
H_sanguineum_295
H_sanguineum_233
H_viscidulum_viscidulum_319
H_pannosum_75_6
H_pannosum_75_5
H_oelandicum_incanum_72
H_marifolium_conquense_55
H_oelandicum_alpestris_184_2
H_oelandicum_italicum_325
H_oelandicum_pourretii_332
H_marifolium_molle_251
H_marifolium_andalusicum_266
H_marifolium_origanifolium_234
H_marifolium_marifolium_85
H_marifolium_frigidulum_267
H_polyanthum_109
H_polyanthum_117
H_viscidulum_raynaudii_70_2
H_cinereum_rotundifolium_72
H_cinereum_hieronymi_62
H_cinereum_cinereum_60
H_cinereum_guadiccianum_64
H_salicifolium_76
H_angustatum_66
H_papillare_106
H_ledifolium_107
H_aegyptiacum_89
H_grosii_118
H_grosii_425
H_kostchianum_268
H_obtusifolium_294
H_ruficomum_111
H_helianthemoides_429
H_helianthemoides_426
H_broussonetii_397
bro_407A
H_juliae_434
H_juliae_404
H_cirae_432
H_cirae_400
H_inaguae_225
H_inaguae_418
H_spNov_2_432
H_spNov_2_398
H_linii_399
H_spNov_1_405
H_bystropogophyllum_224
H_bystropogophyllum_419
H_tholiforme_420
H_teneriffae_401
H_aganae_435
H_spNov_3_437
H_gonzalezferreri_222
H_gonzalezferreri_411
H_bramwelliorum_132
H_bramwelliorum_412
H_polygonoides_59_R2
H_polygonoides_59_R1
H_apenninum_cantabricum_368
H_tinetense_369
H_apenninum_stoechadifolium_236
H_nummularium_grandiflorum_332
H_nummularium_semiglabrum_327
H_nummularium_lycaonicum_269
H_nummularium_obscurum_322
H_nummularium_nummularium_300
H_apenninum_urrielense_104
H_hirtum_55
H_morisianum_395
H_scopulicolum_363
H_apenninum_cavanillesianum_64
H_apenninum_suffruticosum_12
H_neopiliferum_125
H_apenninum_apenninum_239
H_apenninum_estevei_75
H_asperum_65
H_viscarium_62
H_guerrae_61
H_violaceum_317
H_marminorense_276
H_abelardoi_38
H_alypoides_46
H_almeriense_60
H_raskebdanae_274
H_vesicarium_388
H_ciliatum_150
H_pergamaceum_115
H_virgatum_105
H_sauvagei_282
0.02
Sect. Argyrolepis
Sect. Lavandulaceaum
Sect. Pseudomacularia
Sect. Eriocarpum
Subgen. Helianthemum
Sect. Caput-felis
Sect. Macularia
Sect. Atlanthemum
Sect. Pseudocistus
Subgen. Plectolobum
Sect. Brachypetalum
Sect. Helianthemum
Subgen. Helianthemum

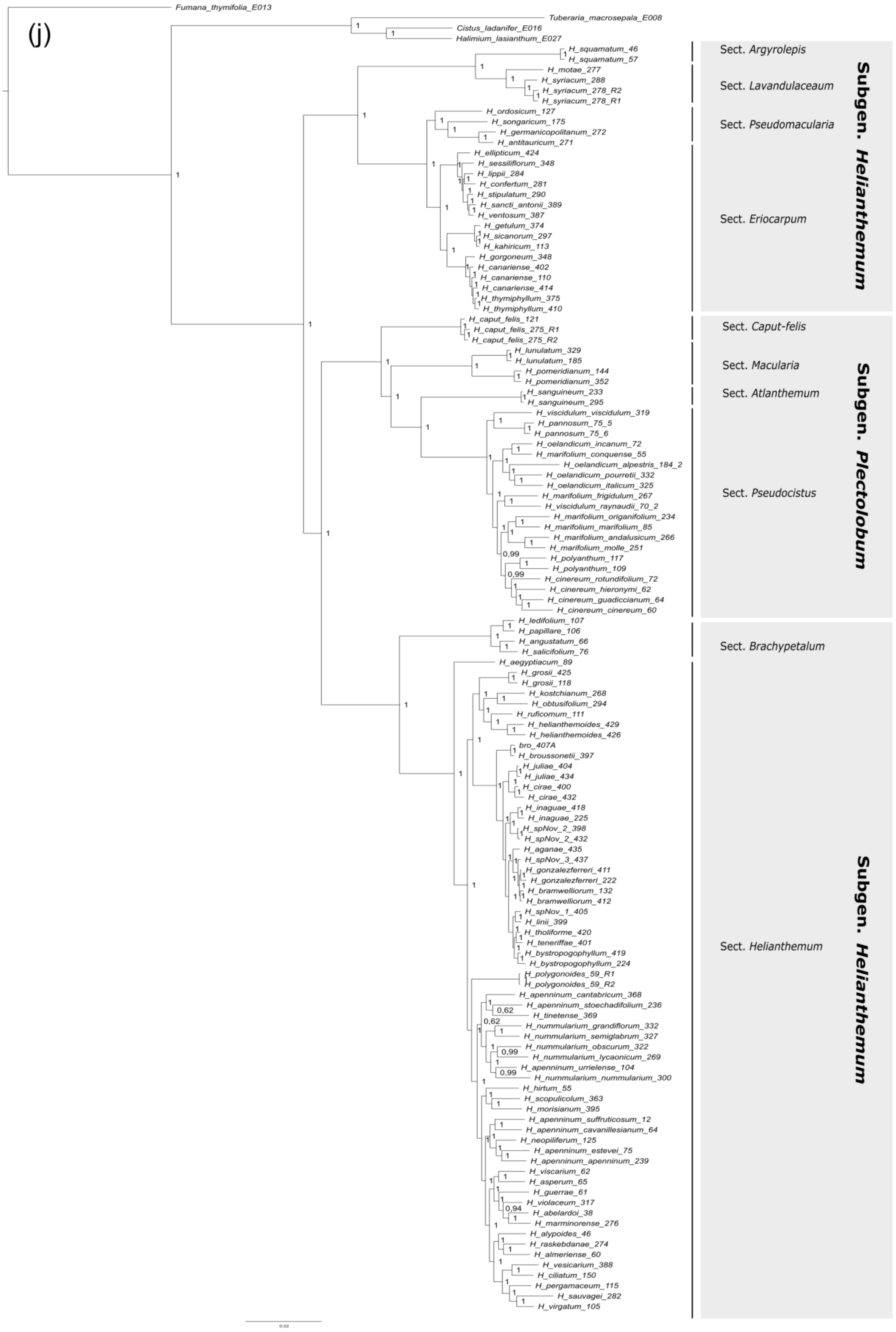
(j)
Fumana_thymifolia_E013
Tuberaria_macrosepala_E008
Cistus_ladanifer_E016
Halimium_lasianthum_E027
H_squamatum_46
H_squamatum_57
H_motae_277
H_syriacum_288
H_syriacum_278_R2
H_syriacum_278_R1
H_ordosicum_127
H_songaricum_175
H_germanicopolitanum_272
H_antitauricum_271
H_ellipticum_424
H_sessiliflorum_348
H_lippii_284
H_confertum_281
H_stipulatum_290
H_sancti_antonii_389
H_ventosum_387
H_getulum_374
H_sicanorum_297
H_kahiricum_113
H_gorgoneum_348
H_canariense_402
H_canariense_110
H_canariense_414
H_thymiphyllum_375
H_thymiphyllum_410
H_caput_felis_121
H_caput_felis_275_R1
H_caput_felis_275_R2
H_lunulatum_329
H_lunulatum_185
H_pomeridianum_144
H_pomeridianum_352
H_sanguineum_233
H_sanguineum_295
H_viscidulum_viscidulum_319
H_pannosum_75_5
H_pannosum_75_6
H_oelandicum_incanum_72
H_marifolium_conquense_55
H_oelandicum_alpestris_184_2
H_oelandicum_pourretii_332
H_oelandicum_italicum_325
H_marifolium_frigidulum_267
H_viscidulum_raynaudii_70_2
H_marifolium_origanifolium_234
H_marifolium_marifolium_85
H_marifolium_andalusicum_266
H_marifolium_molle_251
H_polyanthum_117
H_polyanthum_109
H_cinereum_rotundifolium_72
H_cinereum_hieronymi_62
H_cinereum_guadiccianum_64
H_cinereum_cinereum_60
H_ledifolium_107
H_papillare_106
H_angustatum_66
H_salicifolium_76
H_aegyptiacum_89
H_grosii_425
H_grosii_118
H_kostchianum_268
H_obtusifolium_294
H_ruficomum_111
H_helianthemoides_429
H_helianthemoides_426
bro_407A
H_broussonetii_397
H_juliae_404
H_juliae_434
H_cirae_400
H_cirae_432
H_inaguae_418
H_inaguae_225
H_spNov_2_398
H_spNov_2_432
H_aganae_435
H_spNov_3_437
H_gonzalezferreri_411
H_gonzalezferreri_222
H_bramwelliorum_132
H_bramwelliorum_412
H_spNov_1_405
H_linii_399
H_tholiforme_420
H_teneriffae_401
H_bystropogophyllum_419
H_bystropogophyllum_224
H_polygonoides_59_R1
H_polygonoides_59_R2
H_apenninum_cantabricum_368
H_apenninum_stoechadifolium_236
H_tinetense_369
H_nummularium_grandiflorum_332
H_nummularium_semiglabrum_327
H_nummularium_obscurum_322
H_nummularium_lycaonicum_269
H_apenninum_urrielense_104
H_nummularium_nummularium_300
H_hirtum_55
H_scopulicolum_363
H_morisianum_395
H_apenninum_suffruticosum_12
H_apenninum_cavanillesianum_64
H_neopiliferum_125
H_apenninum_estevei_75
H_apenninum_apenninum_239
H_viscarium_62
H_asperum_65
H_guerrae_61
H_violaceum_317
H_abelardoi_38
H_marminorense_276
H_alypoides_46
H_raskebdanae_274
H_almeriense_60
H_vesicarium_388
H_ciliatum_150
H_pergamaceum_115
H_sauvagei_282
H_virgatum_105
0.02
Sect. Argyrolepis
Sect. Lavandulaceaum
Sect. Pseudomacularia
Sect. Eriocarpum
Subgen. Helianthemum
Sect. Caput-felis
Sect. Macularia
Sect. Atlanthemum
Sect. Pseudocistus
Subgen. Plectolobum
Sect. Brachypetalum
Sect. Helianthemum
Subgen. Helianthemum

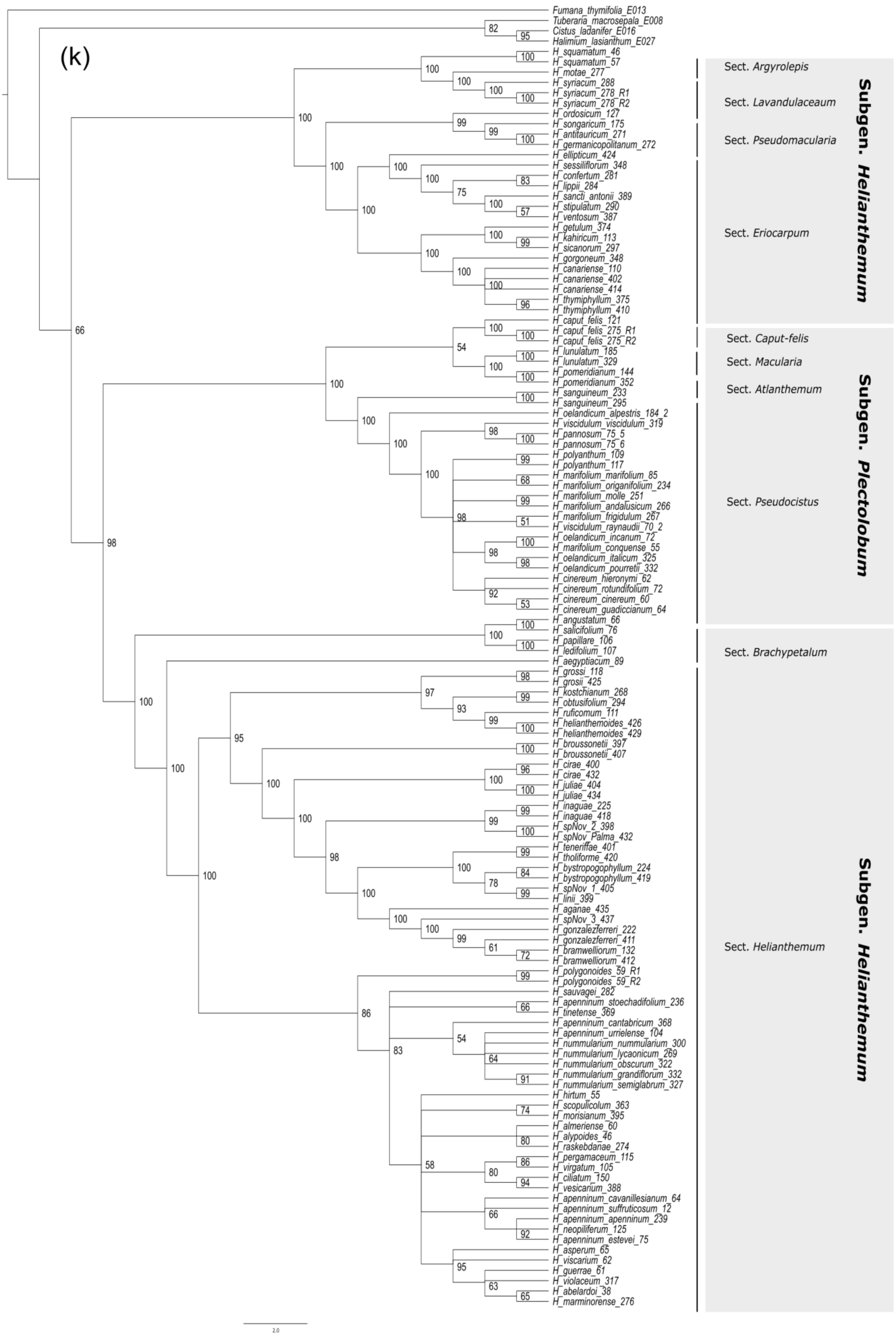
(k)
Fumana_thymifolia_E013
Tuberaria_macrosepala_E008
Cistus_ladanifer_E016
Halimium_lasianthum_E027
H_squamatum_46
H_squamatum_57
H_motae_277
H_syriacum_288
H_syriacum_278_R1
H_syriacum_278_R2
H_ordosicum_127
H_songaricum_175
H_antitauricum_271
H_germanicopolitanum_272
H_ellipticum_424
H_sessiliflorum_348
H_confertum_281
H_lippii_284
H_sancti_antonii_389
H_stipulatum_290
H_ventosum_387
H_getulum_374
H_kahiricum_113
H_sicanorum_297
H_gorgoneum_348
H_canariense_110
H_canariense_402
H_canariense_414
H_thymiphyllum_375
H_thymiphyllum_410
H_caput_felis_121
H_caput_felis_275_R1
H_caput_felis_275_R2
H_lunulatum_185
H_lunulatum_329
H_pomeridianum_144
H_pomeridianum_352
H_sanguineum_233
H_sanguineum_295
H_oelandicum_alpestris_184_2
H_viscidulum_viscidulum_319
H_pannosum_75_5
H_pannosum_75_6
H_polyanthum_109
H_polyanthum_117
H_marifolium_marifolium_85
H_marifolium_origanifolium_234
H_marifolium_molle_251
H_marifolium_andalusicum_266
H_marifolium_frigidulum_267
H_viscidulum_raynaudii_70_2
H_oelandicum_incanum_72
H_marifolium_conquense_55
H_oelandicum_italicum_325
H_oelandicum_pourretii_332
H_cinereum_hieronymi_62
H_cinereum_rotundifolium_72
H_cinereum_cinereum_60
H_cinereum_guadiccianum_64
H_angustatum_66
H_salicifolium_76
H_papillare_106
H_ledifolium_107
H_aegyptiacum_89
H_grossi_118
H_grosii_425
H_kostchianum_268
H_obtusifolium_294
H_ruficomum_111
H_helianthemoides_426
H_helianthemoides_429
H_broussonetii_397
H_broussonetii_407
H_cirae_400
H_cirae_432
H_juliae_404
H_juliae_434
H_inaguae_225
H_inaguae_418
H_spNov_2_398
H_spNov_Palma_432
H_teneriffae_401
H_tholiforme_420
H_bystropogophyllum_224
H_bystropogophyllum_419
H_spNov_1_405
H_linii_399
H_aganae_435
H_spNov_3_437
H_gonzalezferreri_222
H_gonzalezferreri_411
H_bramwelliorum_132
H_bramwelliorum_412
H_polygonoides_59_R1
H_polygonoides_59_R2
H_sauvagei_282
H_apenninum_stoechadifolium_236
H_tinetense_369
H_apenninum_cantabricum_368
H_apenninum_urrielense_104
H_nummularium_nummularium_300
H_nummularium_lycaonicum_269
H_nummularium_obscurum_322
H_nummularium_grandiflorum_332
H_nummularium_semiglabrum_327
H_hirtum_55
H_scopulicolum_363
H_morisianum_395
H_almeriense_60
H_alypoides_46
H_raskebdanae_274
H_pergamaceum_115
H_virgatum_105
H_ciliatum_150
H_vesicarium_388
H_apenninum_cavanillesianum_64
H_apenninum_suffruticosum_12
H_apenninum_apenninum_239
H_neopiliferum_125
H_apenninum_estevei_75
H_asperum_65
H_viscarium_62
H_guerrae_61
H_violaceum_317
H_abelardoi_38
H_marminorense_276
Sect. Argyrolepis
Sect. Lavandulaceaum
Sect. Pseudomacularia
Sect. Eriocarpum
Subgen. Helianthemum
Sect. Caput-felis
Sect. Macularia
Sect. Atlanthemum
Sect. Pseudocistus
Subgen. Plectolobum
Sect. Brachypetalum
Sect. Helianthemum
Subgen. Helianthemum
2.0

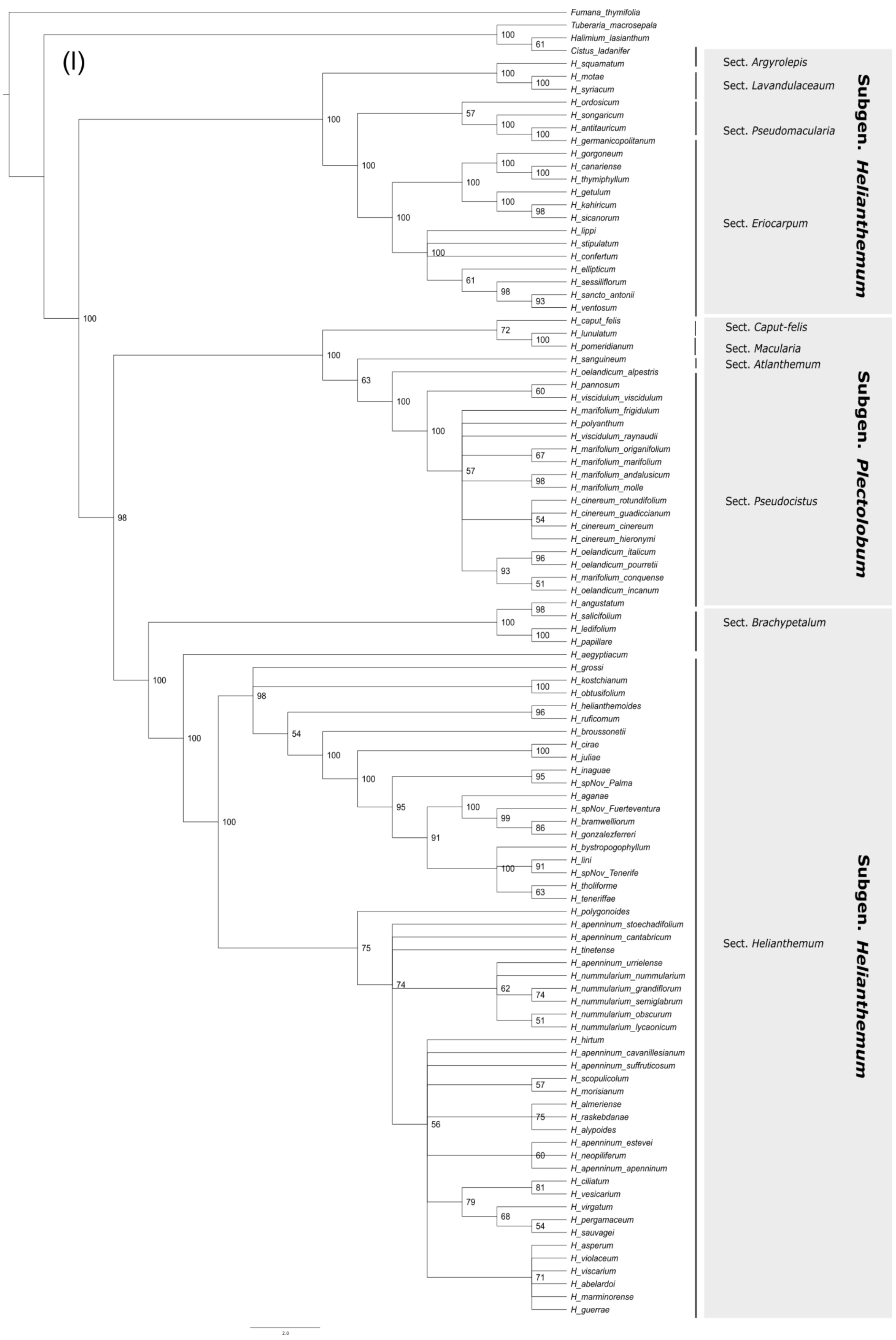
(I)
Fumana_thymifolia
Tuberaria_macrosepala
Halimium_lasianthum
Cistus_ladanifer
H_squamatum
H_motae
H_syriacum
H_ordosicum
H_songaricum
H_antitauricum
H_germanicopolitanum
H_gorgoneum
H_canariense
H_thymiphyllum
H_getulum
H_kahiricum
H_sicanorum
H_lippi
H_stipulatum
H_confertum
H_ellipticum
H_sessiliflorum
H_sancto_antonii
H_ventosum
H_caput_felis
H_lunulatum
H_pomeridianum
H_sanguineum
H_oelandicum_alpestris
H_pannosum
H_viscidulum_viscidulum
H_marifolium_frigidulum
H_polyanthum
H_viscidulum_raynaudii
H_marifolium_origanifolium
H_marifolium_marifolium
H_marifolium_andalusicum
H_marifolium_molle
H_cinereum_rotundifolium
H_cinereum_guadiccianum
H_cinereum_cinereum
H_cinereum_hieronymi
H_oelandicum_italicum
H_oelandicum_pourretii
H_marifolium_conquense
H_oelandicum_incanum
H_angustatum
H_salicifolium
H_ledifolium
H_papillare
H_aegyptiacum
H_grossi
H_kostchianum
H_obtusifolium
H_helianthemoides
H_ruficomum
H_broussonetii
H_cirae
H_juliae
H_inaguae
H_spNov_Palma
H_aganae
H_spNov_Fuerteventura
H_bramwelliorum
H_gonzalezferreri
H_bystropogophyllum
H_lini
H_spNov_Tenerife
H_tholiforme
H_teneriffae
H_polygonoides
H_apenninum_stoechadifolium
H_apenninum_cantabricum
H_tinetense
H_apenninum_urrielense
H_nummularium_nummularium
H_nummularium_grandiflorum
H_nummularium_semiglabrum
H_nummularium_obscurum
H_nummularium_lycaonicum
H_hirtum
H_apenninum_cavanillesianum
H_apenninum_suffruticosum
H_scopulicolum
H_morisianum
H_almeriense
H_raskebdanae
H_alypoides
H_apenninum_estevei
H_neopiliferum
H_apenninum_apenninum
H_ciliatum
H_vesicarium
H_virgatum
H_pergamaceum
H_sauvagei
H_asperum
H_violaceum
H_viscarium
H_abelardoi
H_marminorense
H_guerrae
Sect. Argyrolepis
Sect. Lavandulaceaum
Sect. Pseudomacularia
Sect. Eriocarpum
Subgen. Helianthemum
Sect. Caput-felis
Sect. Macularia
Sect. Atlanthemum
Sect. Pseudocistus
Subgen. Plectolobum
Sect. Brachypetalum
Sect. Helianthemum
Subgen. Helianthemum
2.0

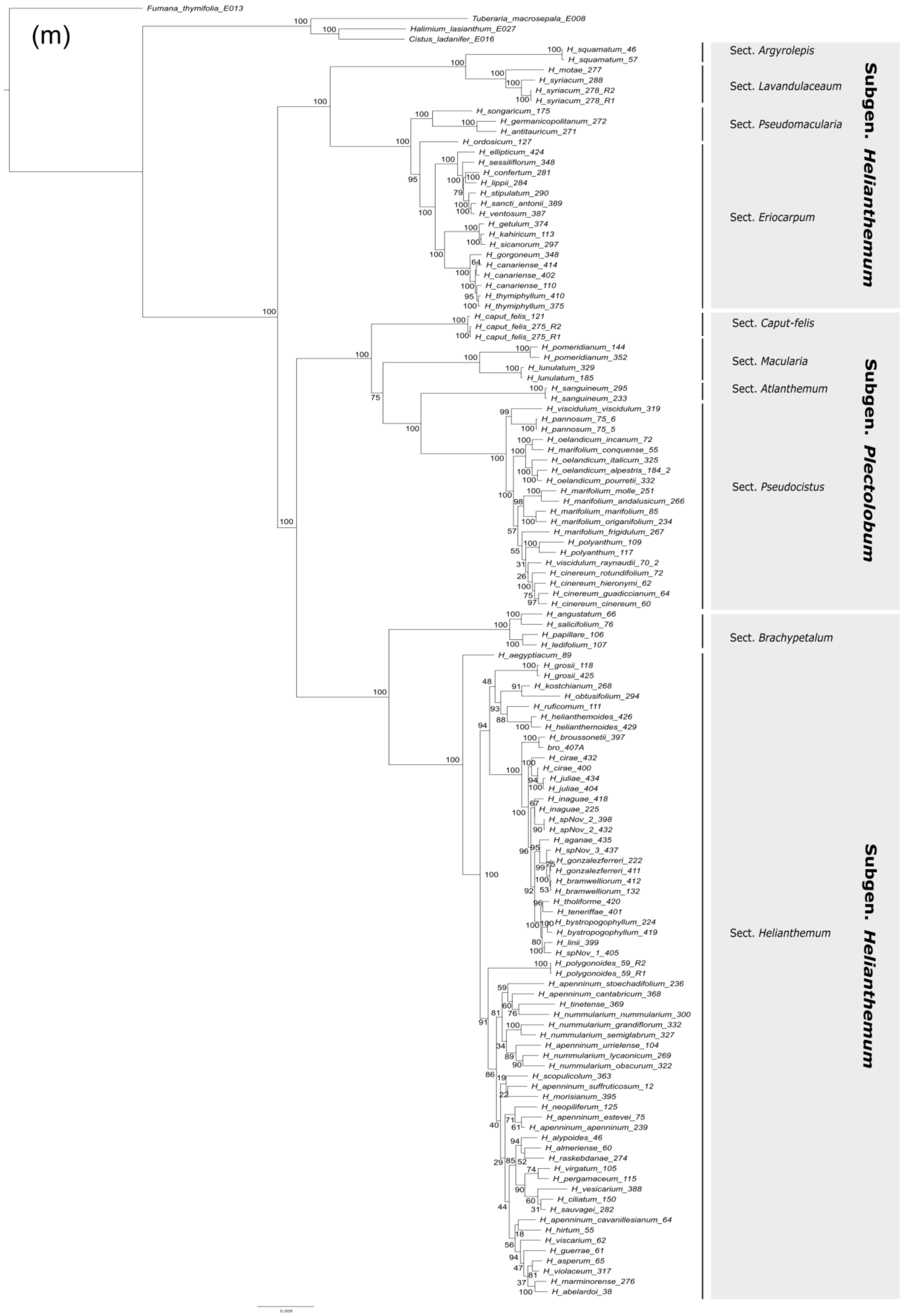
(m)
Fumana_thymifolia_E013
Tuberaria_macrosepala_E008
Halimium_lasianthum_E027
Cistus_ladanifer_E016
H_squamatum_46
H_squamatum_57
H_motae_277
H_syriacum_288
H_syriacum_278_R2
H_syriacum_278_R1
H_songaricum_175
H_germanicopolitanum_272
H_antitauricum_271
H_ordosicum_127
H_ellipticum_424
H_sessiliflorum_348
H_confertum_281
H_lippii_284
H_stipulatum_290
H_sancti_antonii_389
H_ventosum_387
H_getulum_374
H_kahiricum_113
H_sicanorum_297
H_gorgoneum_348
H_canariense_414
H_canariense_402
H_canariense_110
H_thymiphyllum_410
H_thymiphyllum_375
H_caput_felis_121
H_caput_felis_275_R2
H_caput_felis_275_R1
H_pomeridianum_144
H_pomeridianum_352
H_lunulatum_329
H_lunulatum_185
H_sanguineum_295
H_sanguineum_233
H_viscidulum_viscidulum_319
H_pannosum_75_6
H_pannosum_75_5
H_oelandicum_incanum_72
H_marifolium_conquense_55
H_oelandicum_italicum_325
H_oelandicum_alpestris_184_2
H_oelandicum_pourretii_332
H_marifolium_molle_251
H_marifolium_andalusicum_266
H_marifolium_marifolium_85
H_marifolium_origanifolium_234
H_marifolium_frigidulum_267
H_polyanthum_109
H_polyanthum_117
H_viscidulum_raynaudii_70_2
H_cinereum_rotundifolium_72
H_cinereum_hieronymi_62
H_cinereum_guadiccianum_64
H_cinereum_cinereum_60
H_angustatum_66
H_salicifolium_76
H_papillare_106
H_ledifolium_107
H_aegyptiacum_89
H_grosii_118
H_grosii_425
H_kostchianum_268
H_obtusifolium_294
H_ruficomum_111
H_helianthemoides_426
H_helianthemoides_429
H_broussonetii_397
bro_407A
H_cirae_432
H_cirae_400
H_juliae_434
H_juliae_404
H_inaguae_418
H_inaguae_225
H_spNov_2_398
H_spNov_2_432
H_aganae_435
H_spNov_3_437
H_gonzalezferreri_222
H_gonzalezferreri_411
H_bramwelliorum_412
H_bramwelliorum_132
H_tholiforme_420
H_teneriffae_401
H_bystropogophyllum_224
H_bystropogophyllum_419
H_linii_399
H_spNov_1_405
H_polygonoides_59_R2
H_polygonoides_59_R1
H_apenninum_stoechadifolium_236
H_apenninum_cantabricum_368
H_tinetense_369
H_nummularium_nummularium_300
H_nummularium_grandiflorum_332
H_nummularium_semiglabrum_327
H_apenninum_urrielense_104
H_nummularium_lycaonicum_269
H_nummularium_obscurum_322
H_scopulicolum_363
H_apenninum_suffruticosum_12
H_morisianum_395
H_neopiliferum_125
H_apenninum_estevei_75
H_apenninum_apenninum_239
H_alypoides_46
H_almeriense_60
H_raskebdanae_274
H_virgatum_105
H_pergamaceum_115
H_vesicarium_388
H_ciliatum_150
H_sauvagei_282
H_apenninum_cavanillesianum_64
H_hirtum_55
H_viscarium_62
H_guerrae_61
H_asperum_65
H_violaceum_317
H_marminorense_276
H_abelardoi_38
0.009
Sect. Argyrolepis
Sect. Lavandulaceaum
Sect. Pseudomacularia
Sect. Eriocarpum
Subgen. Helianthemum
Sect. Caput-felis
Sect. Macularia
Sect. Atlanthemum
Sect. Pseudocistus
Subgen. Plectolobum
Sect. Brachypetalum
Sect. Helianthemum
Subgen. Helianthemum

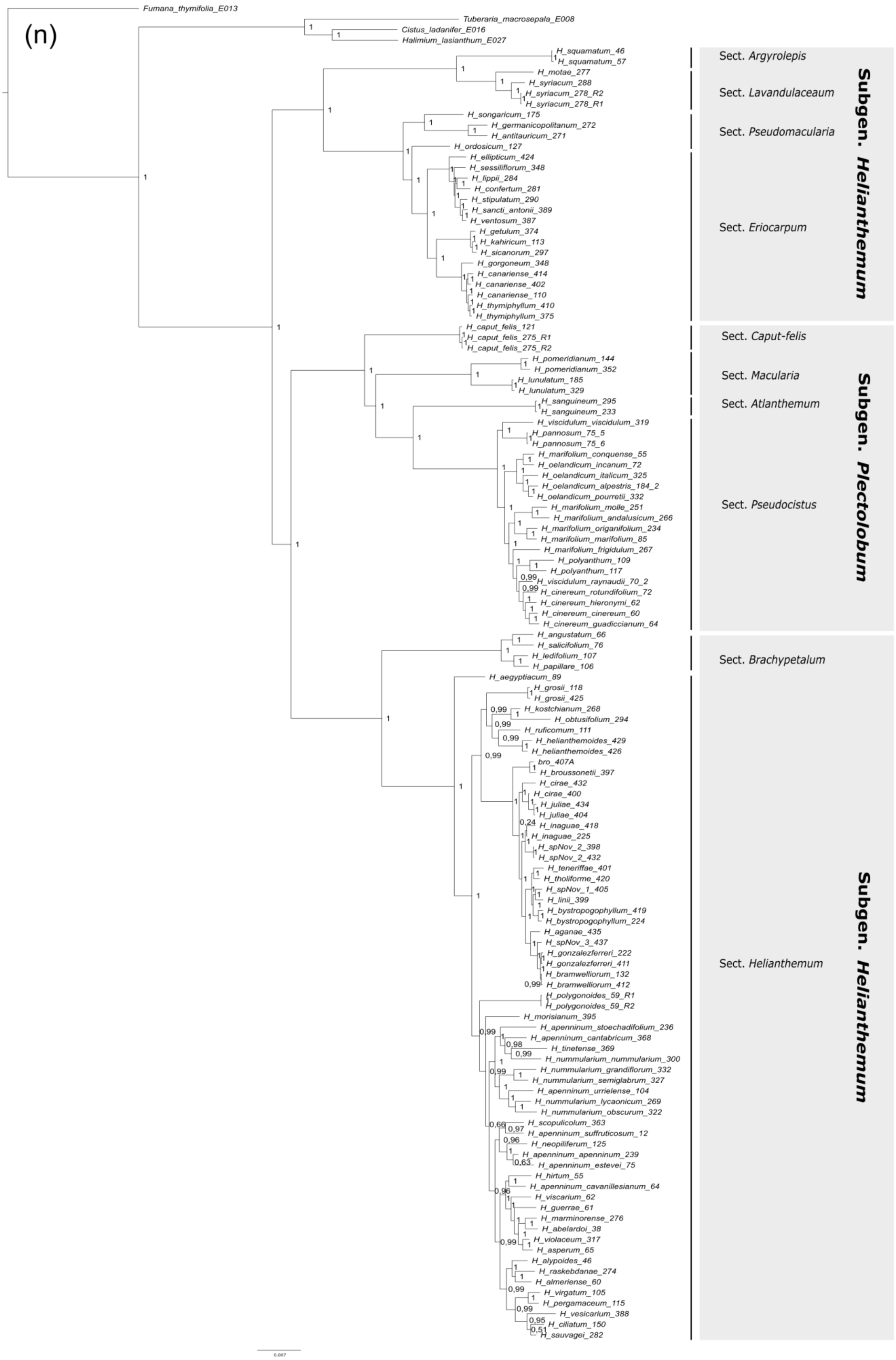
(n)
Fumana_thymifolia_E013
Tuberaria_macrosepala_E008
Cistus_ladanifer_E016
Halimium_lasianthum_E027
H_squamatum_46
H_squamatum_57
H_motae_277
H_syriacum_288
H_syriacum_278_R2
H_syriacum_278_R1
H_songaricum_175
H_germanicopolitanum_272
H_antitauricum_271
H_ordosicum_127
H_ellipticum_424
H_sessiliflorum_348
H_lippii_284
H_confertum_281
H_stipulatum_290
H_sancti_antonii_389
H_ventosum_387
H_getulum_374
H_kahiricum_113
H_sicanorum_297
H_gorgoneum_348
H_canariense_414
H_canariense_402
H_canariense_110
H_thymiphyllum_410
H_thymiphyllum_375
H_caput_felis_121
H_caput_felis_275_R1
H_caput_felis_275_R2
H_pomeridianum_144
H_pomeridianum_352
H_lunulatum_185
H_lunulatum_329
H_sanguineum_295
H_sanguineum_233
H_viscidulum_viscidulum_319
H_pannosum_75_5
H_pannosum_75_6
H_marifolium_conquense_55
H_oelandicum_incanum_72
H_oelandicum_italicum_325
H_oelandicum_alpestris_184_2
H_oelandicum_pourretii_332
H_marifolium_molle_251
H_marifolium_andalusicum_266
H_marifolium_origanifolium_234
H_marifolium_marifolium_85
H_marifolium_frigidulum_267
H_polyanthum_109
H_polyanthum_117
H_viscidulum_raynaudii_70_2
H_cinereum_rotundifolium_72
H_cinereum_hieronymi_62
H_cinereum_cinereum_60
H_cinereum_guadiccianum_64
H_angustatum_66
H_salicifolium_76
H_ledifolium_107
H_papillare_106
H_aegyptiacum_89
H_grosii_118
H_grosii_425
H_kostchianum_268
H_obtusifolium_294
H_ruficomum_111
H_helianthemoides_429
H_helianthemoides_426
bro_407A
H_broussonetii_397
H_cirae_432
H_cirae_400
H_juliae_434
H_juliae_404
H_inaguae_418
H_inaguae_225
H_spNov_2_398
H_spNov_2_432
H_teneriffae_401
H_tholiforme_420
H_spNov_1_405
H_linii_399
H_bystropogophyllum_419
H_bystropogophyllum_224
H_aganae_435
H_spNov_3_437
H_gonzalezferreri_222
H_gonzalezferreri_411
H_bramwelliorum_132
H_bramwelliorum_412
H_polygonoides_59_R1
H_polygonoides_59_R2
H_morisianum_395
H_apenninum_stoechadifolium_236
H_apenninum_cantabricum_368
H_tinetense_369
H_nummularium_nummularium_300
H_nummularium_grandiflorum_332
H_nummularium_semiglabrum_327
H_apenninum_urrielense_104
H_nummularium_lycaonicum_269
H_nummularium_obscurum_322
H_scopulicolum_363
H_apenninum_suffruticosum_12
H_neopiliferum_125
H_apenninum_apenninum_239
H_apenninum_estevei_75
H_hirtum_55
H_apenninum_cavanillesianum_64
H_viscarium_62
H_guerrae_61
H_marminorense_276
H_abelardoi_38
H_violaceum_317
H_asperum_65
H_alypoides_46
H_raskebdanae_274
H_almeriense_60
H_virgatum_105
H_pergamaceum_115
H_vesicarium_388
H_ciliatum_150
H_sauvagei_282
0.007
Sect. Argyrolepis
Sect. Lavandulaceaum
Sect. Pseudomacularia
Sect. Eriocarpum
Subgen. Helianthemum
Sect. Caput-felis
Sect. Macularia
Sect. Atlanthemum
Sect. Pseudocistus
Subgen. Plectolobum
Sect. Brachypetalum
Sect. Helianthemum
Subgen. Helianthemum

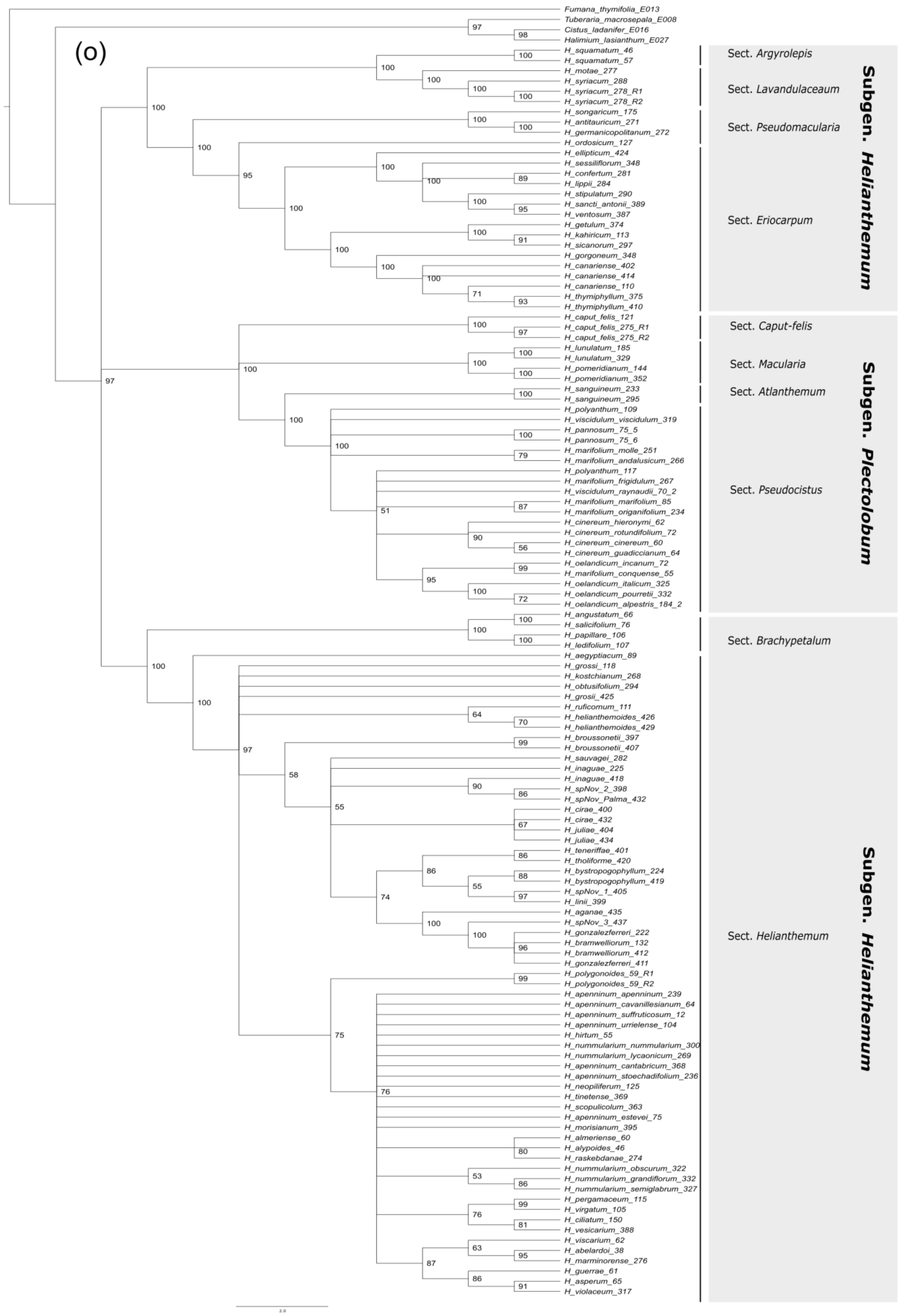
(o)
Fumana_thymifolia_E013
Tuberaria_macrosepala_E008
Cistus_ladanifer_E016
Halimium_lasianthum_E027
H_squamatum_46
H_squamatum_57
H_motae_277
H_syriacum_288
H_syriacum_278_R1
H_syriacum_278_R2
H_songaricum_175
H_antitauricum_271
H_germanicopolitanum_272
H_ordosicum_127
H_ellipticum_424
H_sessiliflorum_348
H_confertum_281
H_lippii_284
H_stipulatum_290
H_sancti_antonii_389
H_ventosum_387
H_getulum_374
H_kahiricum_113
H_sicanorum_297
H_gorgoneum_348
H_canariense_402
H_canariense_414
H_canariense_110
H_thymiphyllum_375
H_thymiphyllum_410
H_caput_felis_121
H_caput_felis_275_R1
H_caput_felis_275_R2
H_lunulatum_185
H_lunulatum_329
H_pomeridianum_144
H_pomeridianum_352
H_sanguineum_233
H_sanguineum_295
H_polyanthum_109
H_viscidulum_viscidulum_319
H_pannosum_75_5
H_pannosum_75_6
H_marifolium_molle_251
H_marifolium_andalusicum_266
H_polyanthum_117
H_marifolium_frigidulum_267
H_viscidulum_raynaudii_70_2
H_marifolium_marifolium_85
H_marifolium_origanifolium_234
H_cinereum_hieronymi_62
H_cinereum_rotundifolium_72
H_cinereum_cinereum_60
H_cinereum_guadiccianum_64
H_oelandicum_incanum_72
H_marifolium_conquense_55
H_oelandicum_italicum_325
H_oelandicum_pourretii_332
H_oelandicum_alpestris_184_2
H_angustatum_66
H_salicifolium_76
H_papillare_106
H_ledifolium_107
H_aegyptiacum_89
H_grossi_118
H_kostchianum_268
H_obtusifolium_294
H_grosii_425
H_ruficomum_111
H_helianthemoides_426
H_helianthemoides_429
H_broussonetii_397
H_broussonetii_407
H_sauvagei_282
H_inaguae_225
H_inaguae_418
H_spNov_2_398
H_spNov_Palma_432
H_cirae_400
H_cirae_432
H_juliae_404
H_juliae_434
H_teneriffae_401
H_tholiforme_420
H_bystropogophyllum_224
H_bystropogophyllum_419
H_spNov_1_405
H_linii_399
H_aganae_435
H_spNov_3_437
H_gonzalezferreri_222
H_bramwelliorum_132
H_bramwelliorum_412
H_gonzalezferreri_411
H_polygonoides_59_R1
H_polygonoides_59_R2
H_apenninum_apenninum_239
H_apenninum_cavanillesianum_64
H_apenninum_suffruticosum_12
H_apenninum_urrielense_104
H_hirtum_55
H_nummularium_nummularium_300
H_nummularium_lycaonicum_269
H_apenninum_cantabricum_368
H_apenninum_stoechadifolium_236
H_neopiliferum_125
H_tinetense_369
H_scopulicolum_363
H_apenninum_estevei_75
H_morisianum_395
H_almeriense_60
H_alypoides_46
H_raskebdanae_274
H_nummularium_obscurum_322
H_nummularium_grandiflorum_332
H_nummularium_semiglabrum_327
H_pergamaceum_115
H_virgatum_105
H_ciliatum_150
H_vesicarium_388
H_viscarium_62
H_abelardoi_38
H_marminorense_276
H_guerrae_61
H_asperum_65
H_violaceum_317
Sect. Argyrolepis
Sect. Lavandulaceaum
Sect. Pseudomacularia
Sect. Eriocarpum
Subgen. Helianthemum
Sect. Caput-felis
Sect. Macularia
Sect. Atlanthemum
Sect. Pseudocistus
Subgen. Plectolobum
Sect. Brachypetalum
Sect. Helianthemum
Subgen. Helianthemum

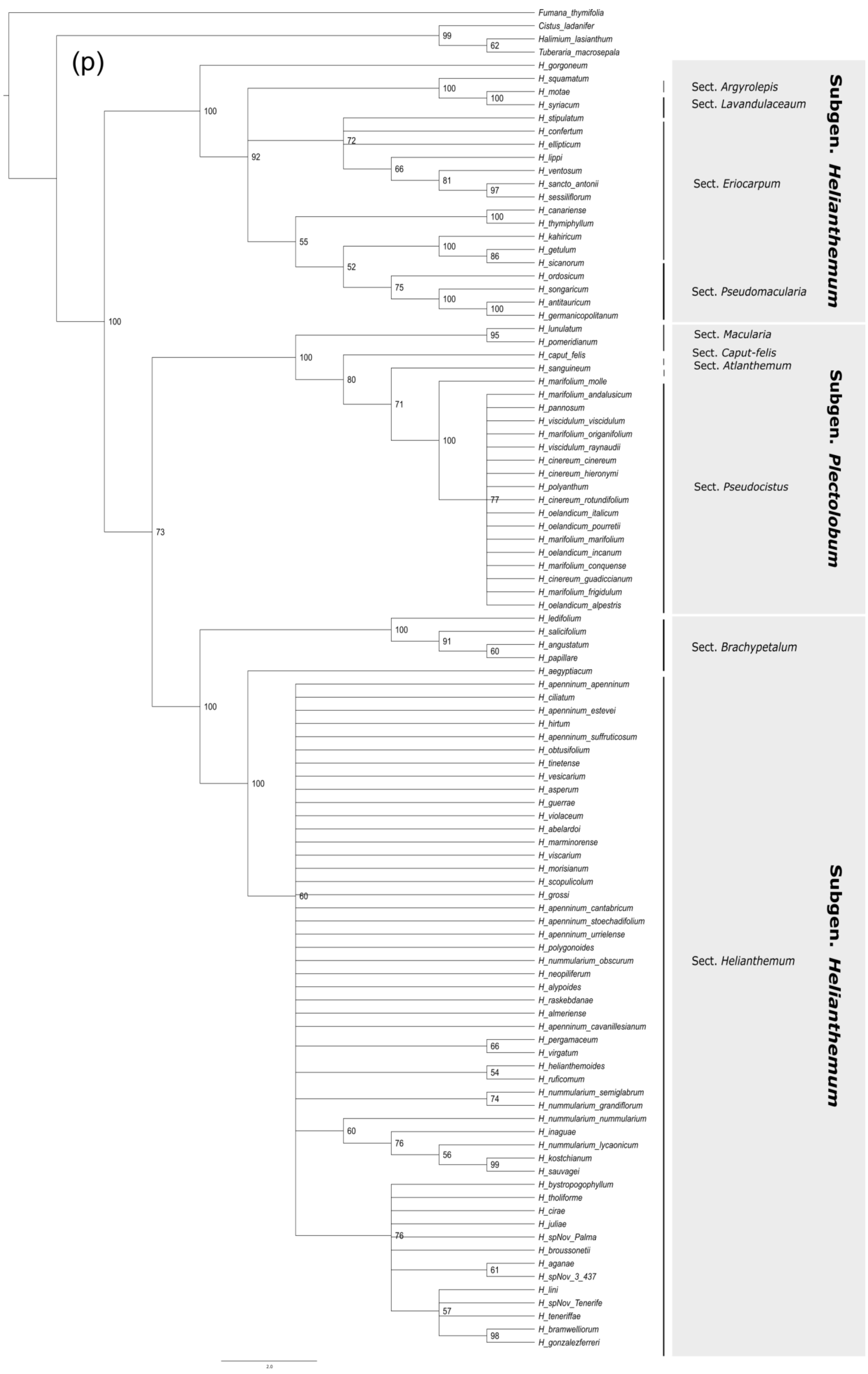

(p)
Fumana_thymifolia
Cistus_ladanifer
Halimium_lasianthum
Tuberaria_macrosepala
H_gorgoneum
H_squamatum
H_motae
H_syriacum
H_stipulatum
H_confertum
H_ellipticum
H_lippi
H_ventosum
H_sancto_antonii
H_sessiliflorum
H_canariense
H_thymiphyllum
H_kahiricum
H_getulum
H_sicanorum
H_ordosicum
H_songaricum
H_antitauricum
H_germanicopolitanum
H_lunulatum
H_pomeridianum
H_caput_felis
H_sanguineum
H_marifolium_molle
H_marifolium_andalusicum
H_pannosum
H_viscidulum_viscidulum
H_marifolium_origanifolium
H_viscidulum_raynaudii
H_cinereum_cinereum
H_cinereum_hieronymi
H_polyanthum
H_cinereum_rotundifolium
H_oelandicum_italicum
H_oelandicum_pourretii
H_marifolium_marifolium
H_oelandicum_incanum
H_marifolium_conquense
H_cinereum_guadiccianum
H_marifolium_frigidulum
H_oelandicum_alpestris
H_ledifolium
H_salicifolium
H_angustatum
H_papillare
H_aegyptiacum
H_apenninum_apenninum
H_ciliatum
H_apenninum_estevei
H_hirtum
H_apenninum_suffruticosum
H_obtusifolium
H_tinetense
H_vesicarium
H_asperum
H_guerrae
H_violaceum
H_abelardoi
H_marminorense
H_viscarium
H_morisianum
H_scopulicolum
H_grossi
H_apenninum_cantabricum
H_apenninum_stoechadifolium
H_apenninum_urrielense
H_polygonoides
H_nummularium_obscurum
H_neopiliferum
H_alypoides
H_raskebdanae
H_almeriense
H_apenninum_cavanillesianum
H_pergamaceum
H_virgatum
H_helianthemoides
H_ruficomum
H_nummularium_semiglabrum
H_nummularium_grandiflorum
H_nummularium_nummularium
H_inaguae
H_nummularium_lycaonicum
H_kostchianum
H_sauvagei
H_bystropogophyllum
H_tholiforme
H_cirae
H_juliae
H_spNov_Palma
H_broussonetii
H_aganae
H_spNov_3_437
H_lini
H_spNov_Tenerife
H_teneriffae
H_bramwelliorum
H_gonzalezferreri
Sect. Argyrolepis
Sect. Lavandulaceaum
Sect. Eriocarpum
Sect. Pseudomacularia
Subgen. Helianthemum
Sect. Macularia
Sect. Caput-felis
Sect. Atlanthemum
Sect. Pseudocistus
Subgen. Plectolobum
Sect. Brachypetalum
Sect. Helianthemum
Subgen. Helianthemum
2.0

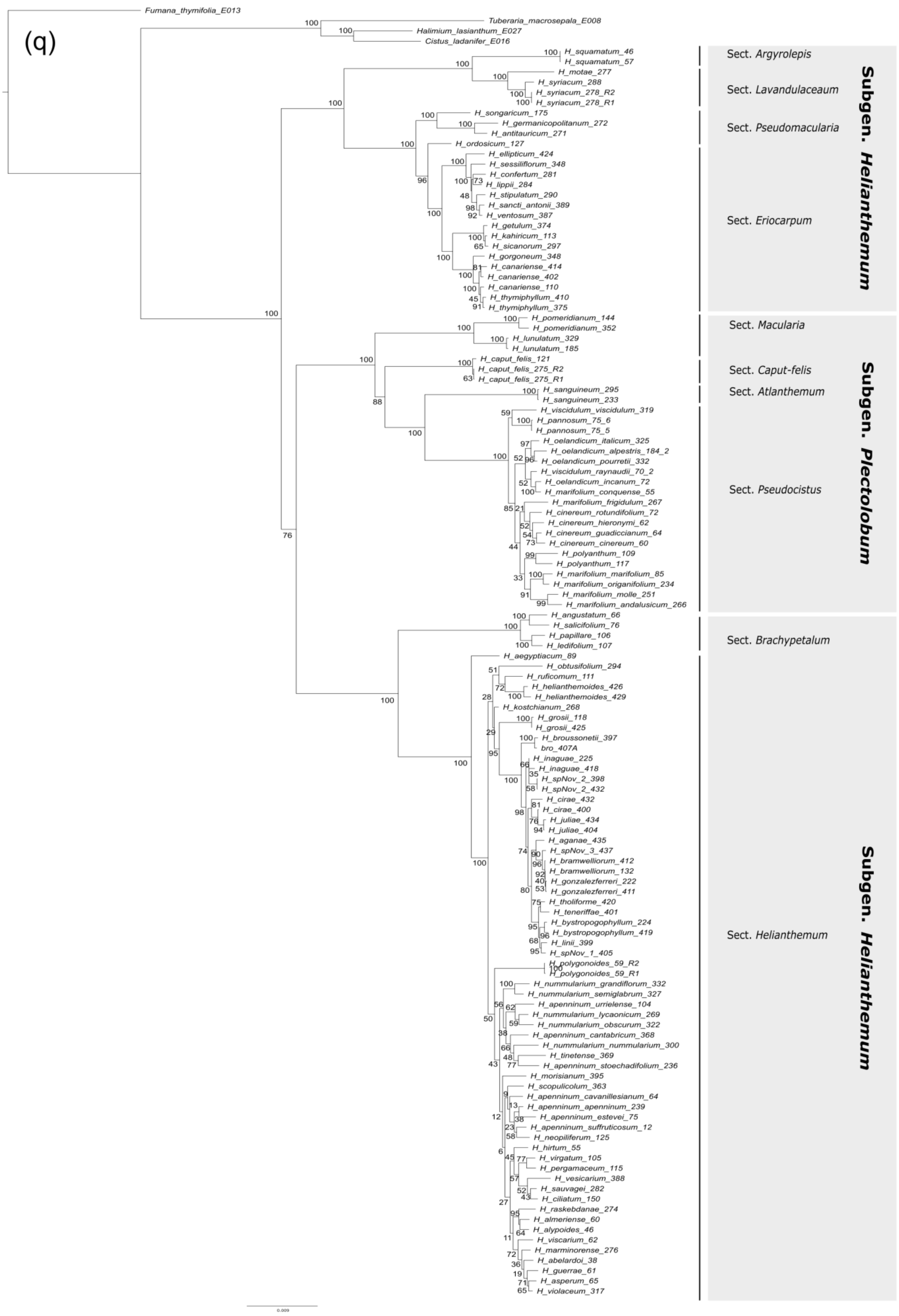

(q)
Sect. Argyrolepis
Sect. Lavandulaceaum
Sect. Pseudomacularia
Sect. Eriocarpum
Subgen. Helianthemum
Sect. Macularia
Sect. Caput-felis
Sect. Atlanthemum
Sect. Pseudocistus
Subgen. Plectolobum
Sect. Brachypetalum
Sect. Helianthemum
Subgen. Helianthemum

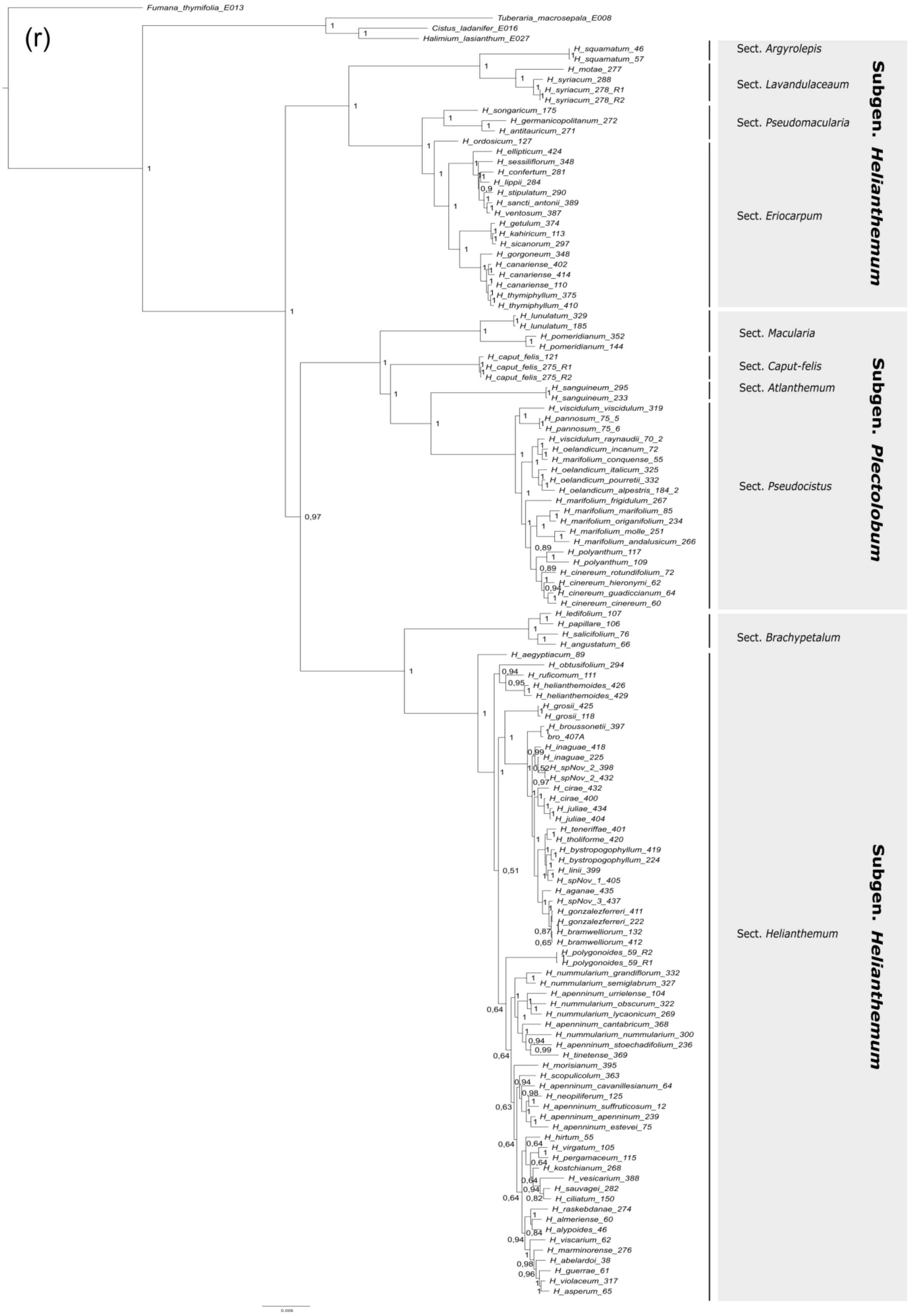

(r)
Fumana_thymifolia_E013
Tuberaria_macrosepala_E008
Cistus_ladanifer_E016
Halimium_lasianthum_E027
H_squamatum_46
H_squamatum_57
H_motae_277
H_syriacum_288
H_syriacum_278_R1
H_syriacum_278_R2
H_songaricum_175
H_germanicopolitanum_272
H_antitauricum_271
H_ordosicum_127
H_ellipticum_424
H_sessiliflorum_348
H_confertum_281
H_lippii_284
H_stipulatum_290
H_sancti_antonii_389
H_ventosum_387
H_getulum_374
H_kahiricum_113
H_sicanorum_297
H_gorgoneum_348
H_canariense_402
H_canariense_414
H_canariense_110
H_thymiphyllum_375
H_thymiphyllum_410
H_lunulatum_329
H_lunulatum_185
H_pomeridianum_352
H_pomeridianum_144
H_caput_felis_121
H_caput_felis_275_R1
H_caput_felis_275_R2
H_sanguineum_295
H_sanguineum_233
H_viscidulum_viscidulum_319
H_pannosum_75_5
H_pannosum_75_6
H_viscidulum_raynaudii_70_2
H_oelandicum_incanum_72
H_marifolium_conquense_55
H_oelandicum_italicum_325
H_oelandicum_pourretii_332
H_oelandicum_alpestris_184_2
H_marifolium_frigidulum_267
H_marifolium_marifolium_85
H_marifolium_origanifolium_234
H_marifolium_molle_251
H_marifolium_andalusicum_266
H_polyanthum_117
H_polyanthum_109
H_cinereum_rotundifolium_72
H_cinereum_hieronymi_62
H_cinereum_guadiccianum_64
H_cinereum_cinereum_60
H_ledifolium_107
H_papillare_106
H_salicifolium_76
H_angustatum_66
H_aegyptiacum_89
H_obtusifolium_294
H_ruficomum_111
H_helianthemoides_426
H_helianthemoides_429
H_grosii_425
H_grosii_118
H_broussonetii_397
bro_407A
H_inaguae_418
H_inaguae_225
H_spNov_2_398
H_spNov_2_432
H_cirae_432
H_cirae_400
H_juliae_434
H_juliae_404
H_teneriffae_401
H_tholiforme_420
H_bystropogophyllum_419
H_bystropogophyllum_224
H_linii_399
H_spNov_1_405
H_aganae_435
H_spNov_3_437
H_gonzalezferreri_411
H_gonzalezferreri_222
H_bramwelliorum_132
H_bramwelliorum_412
H_polygonoides_59_R2
H_polygonoides_59_R1
H_nummularium_grandiflorum_332
H_nummularium_semiglabrum_327
H_apenninum_urrielense_104
H_nummularium_obscurum_322
H_nummularium_lycaonicum_269
H_apenninum_cantabricum_368
H_nummularium_nummularium_300
H_apenninum_stoechadifolium_236
H_tinetense_369
H_morisianum_395
H_scopulicolum_363
H_apenninum_cavanillesianum_64
H_neopiliferum_125
H_apenninum_suffruticosum_12
H_apenninum_apenninum_239
H_apenninum_estevei_75
H_hirtum_55
H_virgatum_105
H_pergamaceum_115
H_kostchianum_268
H_vesicarium_388
H_sauvagei_282
H_ciliatum_150
H_raskebdanae_274
H_almeriense_60
H_alypoides_46
H_viscarium_62
H_marminorense_276
H_abelardoi_38
H_guerrae_61
H_violaceum_317
H_asperum_65
Sect. Argyrolepis
Sect. Lavandulaceaum
Sect. Pseudomacularia
Sect. Eriocarpum
Subgen. Helianthemum
Sect. Macularia
Sect. Caput-felis
Sect. Atlanthemum
Sect. Pseudocistus
Subgen. Plectolobum
Sect. Brachypetalum
Sect. Helianthemum
Subgen. Helianthemum
0.006

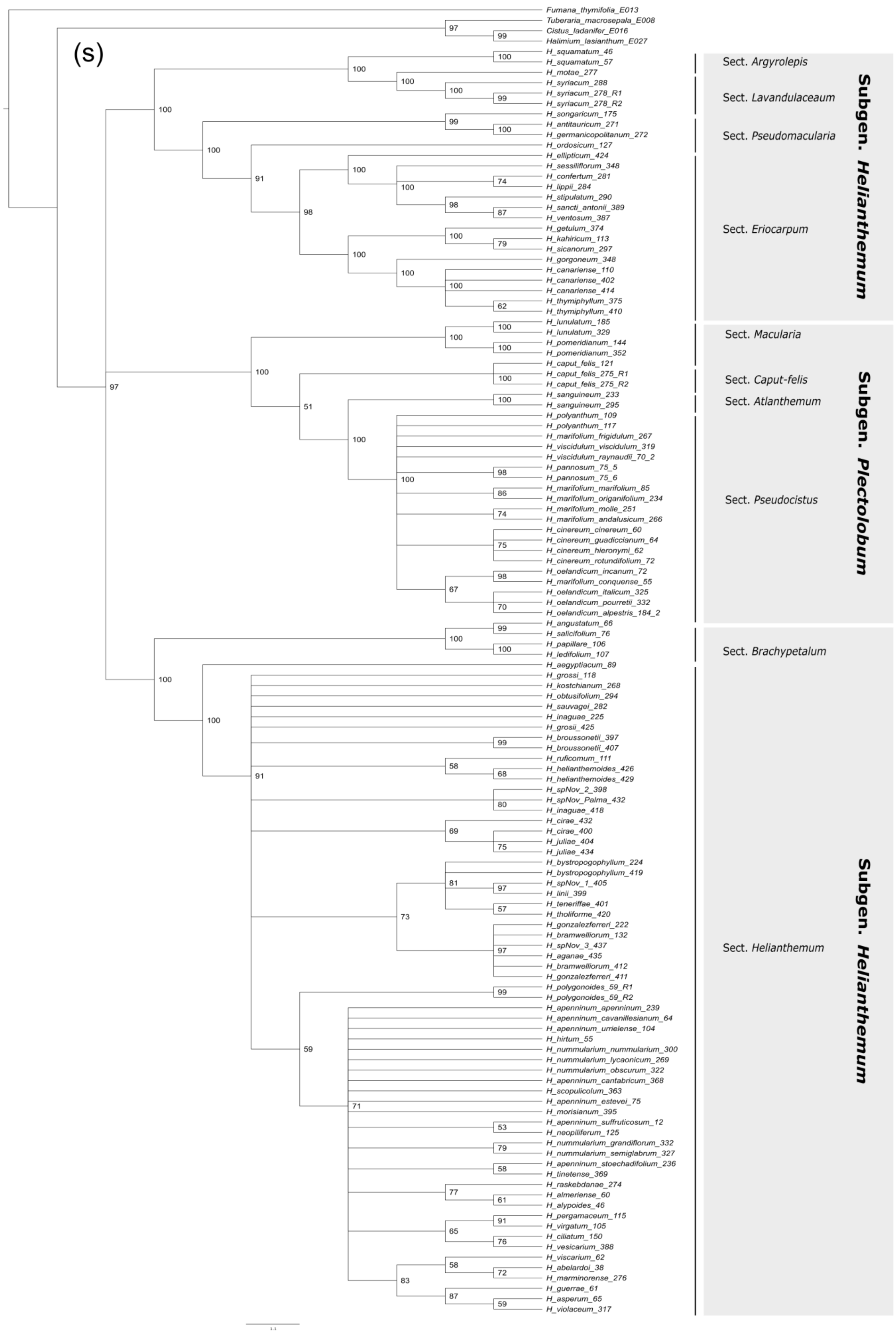
(s)
Fumana_thymifolia_E013
Tuberaria_macrosepala_E008
Cistus_ladanifer_E016
Halimium_lasianthum_E027
H_squamatum_46
H_squamatum_57
H_motae_277
H_syriacum_288
H_syriacum_278_R1
H_syriacum_278_R2
H_songaricum_175
H_antitauricum_271
H_germanicopolitanum_272
H_ordosicum_127
H_ellipticum_424
H_sessiliflorum_348
H_confertum_281
H_lippii_284
H_stipulatum_290
H_sancti_antonii_389
H_ventosum_387
H_getulum_374
H_kahiricum_113
H_sicanorum_297
H_gorgoneum_348
H_canariense_110
H_canariense_402
H_canariense_414
H_thymiphyllum_375
H_thymiphyllum_410
H_lunulatum_185
H_lunulatum_329
H_pomeridianum_144
H_pomeridianum_352
H_caput_felis_121
H_caput_felis_275_R1
H_caput_felis_275_R2
H_sanguineum_233
H_sanguineum_295
H_polyanthum_109
H_polyanthum_117
H_marifolium_frigidulum_267
H_viscidulum_viscidulum_319
H_viscidulum_raynaudii_70_2
H_pannosum_75_5
H_pannosum_75_6
H_marifolium_marifolium_85
H_marifolium_origanifolium_234
H_marifolium_molle_251
H_marifolium_andalusicum_266
H_cinereum_cinereum_60
H_cinereum_guadiccianum_64
H_cinereum_hieronymi_62
H_cinereum_rotundifolium_72
H_oelandicum_incanum_72
H_marifolium_conquense_55
H_oelandicum_italicum_325
H_oelandicum_pourretii_332
H_oelandicum_alpestris_184_2
H_angustatum_66
H_salicifolium_76
H_papillare_106
H_ledifolium_107
H_aegyptiacum_89
H_grossi_118
H_kostchianum_268
H_obtusifolium_294
H_sauvagei_282
H_inaguae_225
H_grosii_425
H_broussonetii_397
H_broussonetii_407
H_ruficomum_111
H_helianthemoides_426
H_helianthemoides_429
H_spNov_2_398
H_spNov_Palma_432
H_inaguae_418
H_cirae_432
H_cirae_400
H_juliae_404
H_juliae_434
H_bystropogophyllum_224
H_bystropogophyllum_419
H_spNov_1_405
H_linii_399
H_teneriffae_401
H_tholiforme_420
H_gonzalezferreri_222
H_bramwelliorum_132
H_spNov_3_437
H_aganae_435
H_bramwelliorum_412
H_gonzalezferreri_411
H_polygonoides_59_R1
H_polygonoides_59_R2
H_apenninum_apenninum_239
H_apenninum_cavanillesianum_64
H_apenninum_urrielense_104
H_hirtum_55
H_nummularium_nummularium_300
H_nummularium_lycaonicum_269
H_nummularium_obscurum_322
H_apenninum_cantabricum_368
H_scopulicolum_363
H_apenninum_estevei_75
H_morisianum_395
H_apenninum_suffruticosum_12
H_neopiliferum_125
H_nummularium_grandiflorum_332
H_nummularium_semiglabrum_327
H_apenninum_stoechadifolium_236
H_tinetense_369
H_raskebdanae_274
H_almeriense_60
H_alypoides_46
H_pergamaceum_115
H_virgatum_105
H_ciliatum_150
H_vesicarium_388
H_viscarium_62
H_abelardoi_38
H_marminorense_276
H_guerrae_61
H_asperum_65
H_violaceum_317
Sect. Argyrolepis
Sect. Lavandulaceaum
Sect. Pseudomacularia
Sect. Eriocarpum
Subgen. Helianthemum
Sect. Macularia
Sect. Caput-felis
Sect. Atlanthemum
Sect. Pseudocistus
Subgen. Plectolobum
Sect. Brachypetalum
Sect. Helianthemum
Subgen. Helianthemum

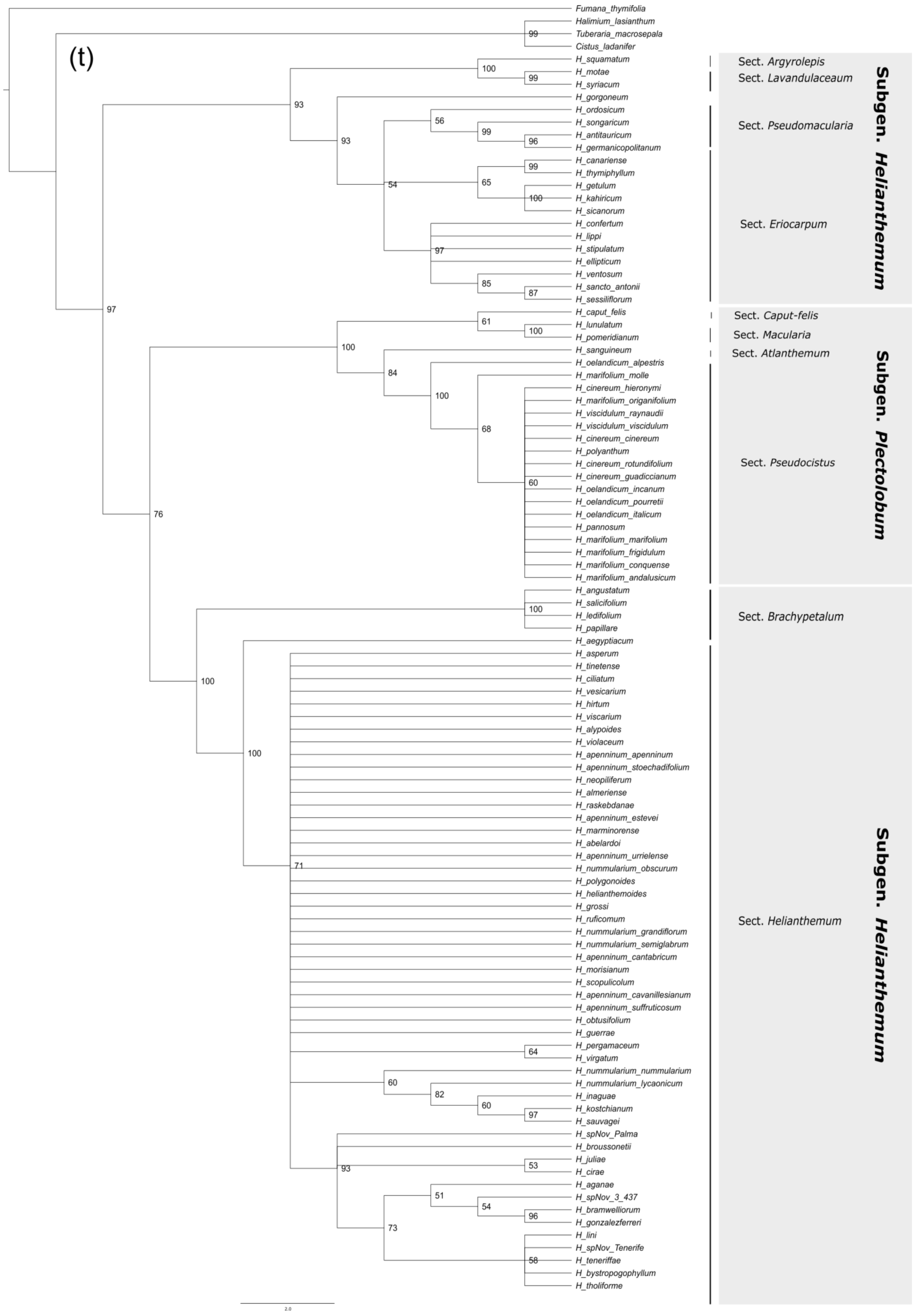
(t)
Fumana_thymifolia
Halimium_lasianthum
Tuberaria_macrosepala
Cistus_ladanifer
H_squamatum
H_motae
H_syriacum
H_gorgoneum
H_ordosicum
H_songaricum
H_antitauricum
H_germanicopolitanum
H_canariense
H_thymiphyllum
H_getulum
H_kahiricum
H_sicanorum
H_confertum
H_lippi
H_stipulatum
H_ellipticum
H_ventosum
H_sancto_antonii
H_sessiliflorum
H_caput_felis
H_lunulatum
H_pomeridianum
H_sanguineum
H_oelandicum_alpestris
H_marifolium_molle
H_cinereum_hieronymi
H_marifolium_origanifolium
H_viscidulum_raynaudii
H_viscidulum_viscidulum
H_cinereum_cinereum
H_polyanthum
H_cinereum_rotundifolium
H_cinereum_guadiccianum
H_oelandicum_incanum
H_oelandicum_pourretii
H_oelandicum_italicum
H_pannosum
H_marifolium_marifolium
H_marifolium_frigidulum
H_marifolium_conquense
H_marifolium_andalusicum
H_angustatum
H_salicifolium
H_ledifolium
H_papillare
H_aegyptiacum
H_asperum
H_tinetense
H_ciliatum
H_vesicarium
H_hirtum
H_viscarium
H_alypoides
H_violaceum
H_apenninum_apenninum
H_apenninum_stoechadifolium
H_neopiliferum
H_almeriense
H_raskebdanae
H_apenninum_estevei
H_marminorense
H_abelardoi
H_apenninum_urrielense
H_nummularium_obscurum
H_polygonoides
H_helianthemoides
H_grossi
H_ruficomum
H_nummularium_grandiflorum
H_nummularium_semiglabrum
H_apenninum_cantabricum
H_morisianum
H_scopulicolum
H_apenninum_cavanillesianum
H_apenninum_suffruticosum
H_obtusifolium
H_guerrae
H_pergamaceum
H_virgatum
H_nummularium_nummularium
H_nummularium_lycaonicum
H_inaguae
H_kostchianum
H_sauvagei
H_spNov_Palma
H_broussonetii
H_juliae
H_cirae
H_aganae
H_spNov_3_437
H_bramwelliorum
H_gonzalezferreri
H_lini
H_spNov_Tenerife
H_teneriffae
H_bystropogophyllum
H_tholiforme
2.0
Sect. Argyrolepis
Sect. Lavandulaceaum
Sect. Pseudomacularia
Sect. Eriocarpum
Subgen. Helianthemum
Sect. Caput-felis
Sect. Macularia
Sect. Atlanthemum
Sect. Pseudocistus
Subgen. Plectolobum
Sect. Brachypetalum
Sect. Helianthemum
Subgen. Helianthemum

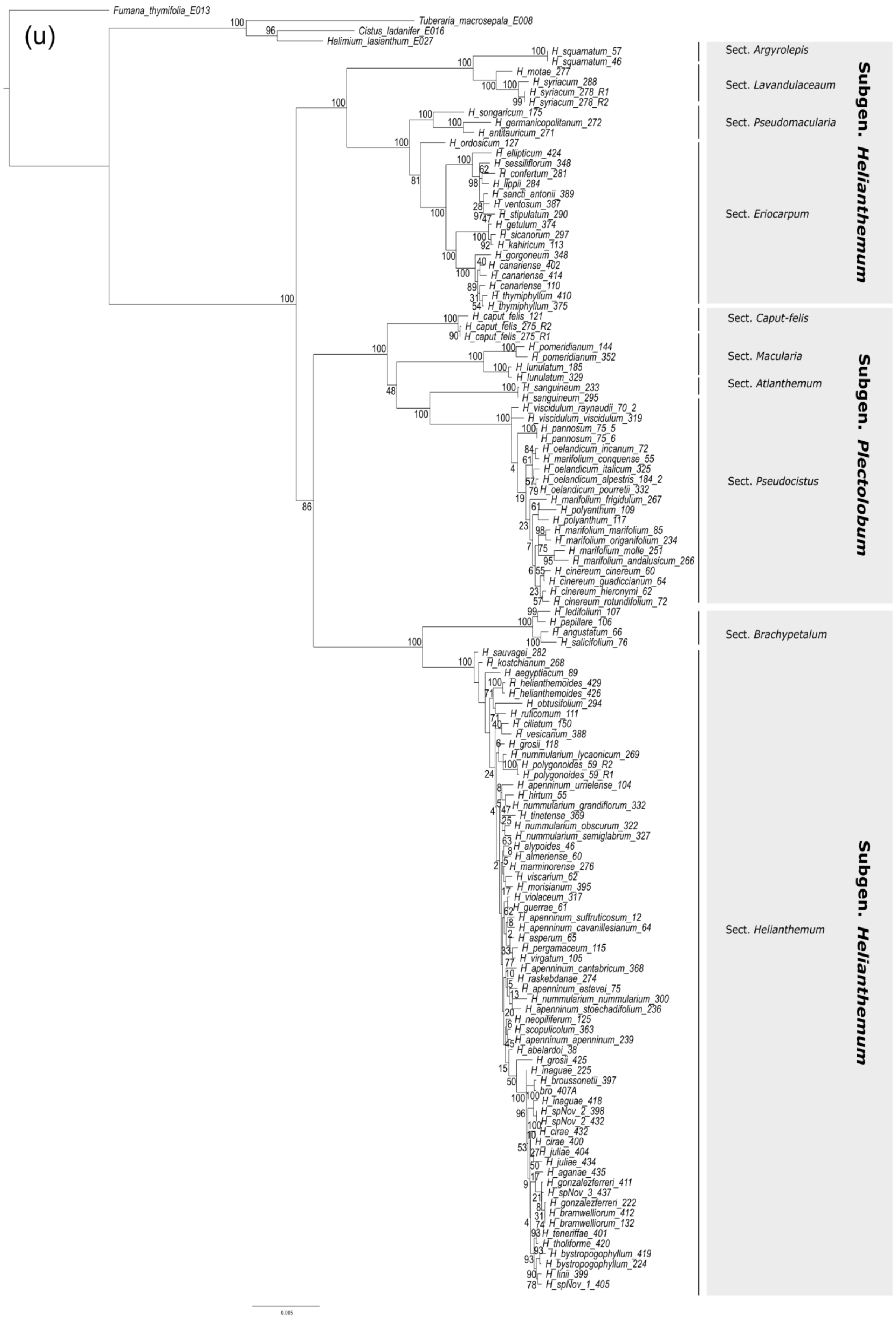

(u)
Fumana_thymifolia_E013
Tuberaria_macrosepala_E008
Cistus_ladanifer_E016
Halimium_lasianthum_E027
H_squamatum_57
H_squamatum_46
H_motae_277
H_syriacum_288
H_syriacum_278_R1
H_syriacum_278_R2
H_songaricum_175
H_germanicopolitanum_272
H_antitauricum_271
H_ordosicum_127
H_ellipticum_424
H_sessiliflorum_348
H_confertum_281
H_lippii_284
H_sancti_antonii_389
H_ventosum_387
H_stipulatum_290
H_getulum_374
H_sicanorum_297
H_kahiricum_113
H_gorgoneum_348
H_canariense_402
H_canariense_414
H_canariense_110
H_thymiphyllum_410
H_thymiphyllum_375
H_caput_felis_121
H_caput_felis_275_R2
H_caput_felis_275_R1
H_pomeridianum_144
H_pomeridianum_352
H_lunulatum_185
H_lunulatum_329
H_sanguineum_233
H_sanguineum_295
H_viscidulum_raynaudii_70_2
H_viscidulum_viscidulum_319
H_pannosum_75_5
H_pannosum_75_6
H_oelandicum_incanum_72
H_marifolium_conquense_55
H_oelandicum_italicum_325
H_oelandicum_alpestris_184_2
H_oelandicum_pourretii_332
H_marifolium_frigidulum_267
H_polyanthum_109
H_polyanthum_117
H_marifolium_marifolium_85
H_marifolium_origanifolium_234
H_marifolium_molle_251
H_marifolium_andalusicum_266
H_cinereum_cinereum_60
H_cinereum_guadiccianum_64
H_cinereum_hieronymi_62
H_cinereum_rotundifolium_72
H_ledifolium_107
H_papillare_106
H_angustatum_66
H_salicifolium_76
H_sauvagei_282
H_kostchianum_268
H_aegyptiacum_89
H_helianthemoides_429
H_helianthemoides_426
H_obtusifolium_294
H_ruficomum_111
H_ciliatum_150
H_vesicarium_388
H_grosii_118
H_nummularium_lycaonicum_269
H_polygonoides_59_R2
H_polygonoides_59_R1
H_apenninum_urrielense_104
H_hirtum_55
H_nummularium_grandiflorum_332
H_tinetense_369
H_nummularium_obscurum_322
H_nummularium_semiglabrum_327
H_alypoides_46
H_almeriense_60
H_marminorense_276
H_viscarium_62
H_morisianum_395
H_violaceum_317
H_guerrae_61
H_apenninum_suffruticosum_12
H_apenninum_cavanillesianum_64
H_asperum_65
H_pergamaceum_115
H_virgatum_105
H_apenninum_cantabricum_368
H_raskebdanae_274
H_apenninum_estevei_75
H_nummularium_nummularium_300
H_apenninum_stoechadifolium_236
H_neopiliferum_125
H_scopulicolum_363
H_apenninum_apenninum_239
H_abelardoi_38
H_grosii_425
H_inaguae_225
H_broussonetii_397
bro_407A
H_inaguae_418
H_spNov_2_398
H_spNov_2_432
H_cirae_432
H_cirae_400
H_juliae_404
H_juliae_434
H_aganae_435
H_gonzalezferreri_411
H_spNov_3_437
H_gonzalezferreri_222
H_bramwelliorum_412
H_bramwelliorum_132
H_teneriffae_401
H_tholiforme_420
H_bystropogophyllum_419
H_bystropogophyllum_224
H_linii_399
H_spNov_1_405
Sect. Argyrolepis
Sect. Lavandulaceaum
Sect. Pseudomacularia
Sect. Eriocarpum
Subgen. Helianthemum
Sect. Caput-felis
Sect. Macularia
Sect. Atlanthemum
Sect. Pseudocistus
Subgen. Plectolobum
Sect. Brachypetalum
Sect. Helianthemum
Subgen. Helianthemum
0.005

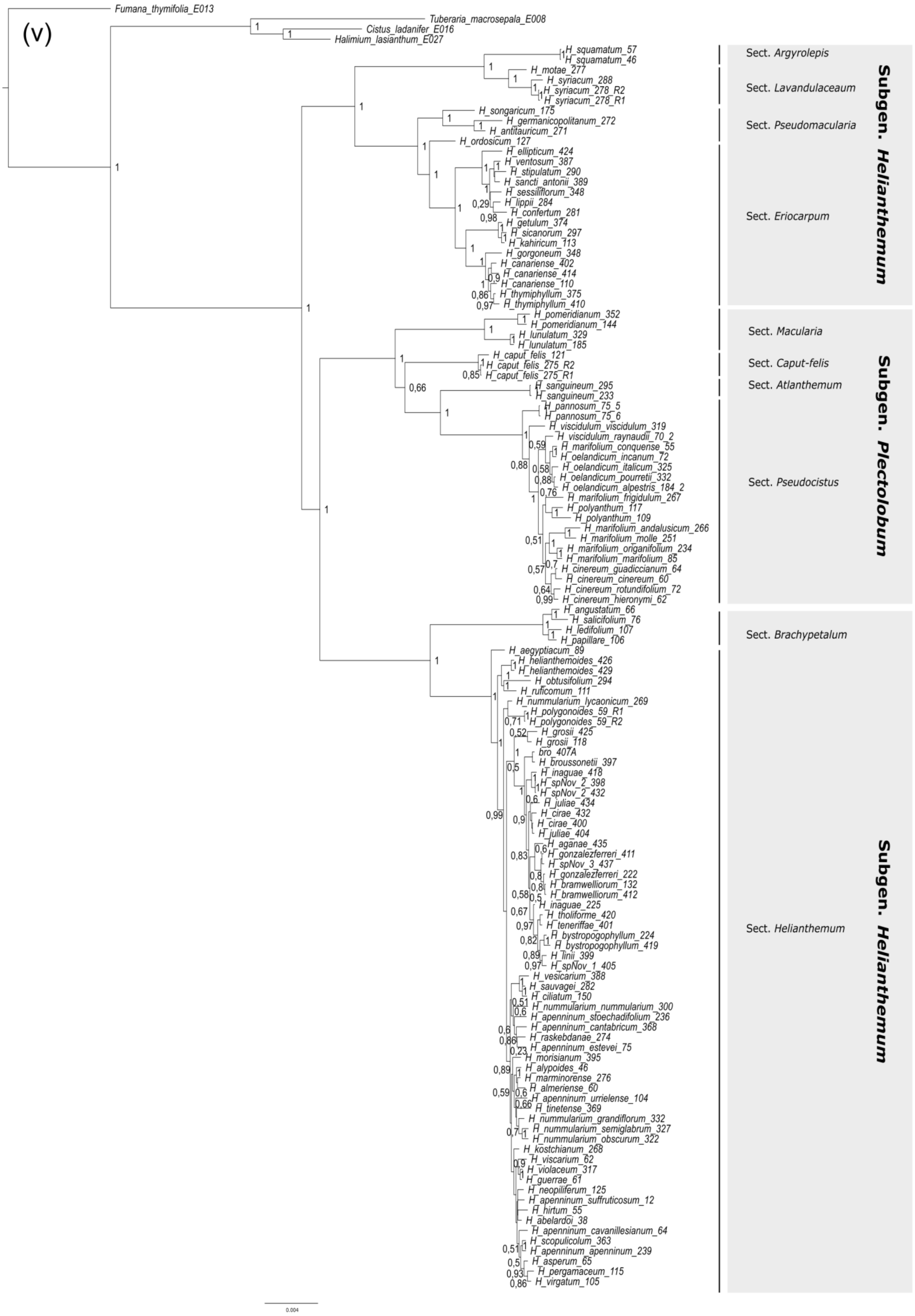
(v)
Fumana_thymifolia_E013
Tuberaria_macrosepala_E008
Cistus_ladanifer_E016
Halimium_lasianthum_E027
H_squamatum_57
H_squamatum_46
H_motae_277
H_syriacum_288
H_syriacum_278_R2
H_syriacum_278_R1
H_songaricum_175
H_germanicopolitanum_272
H_antitauricum_271
H_ordosicum_127
H_ellipticum_424
H_ventosum_387
H_stipulatum_290
H_sancti_antonii_389
H_sessiliflorum_348
H_lippii_284
H_confertum_281
H_getulum_374
H_sicanorum_297
H_kahiricum_113
H_gorgoneum_348
H_canariense_402
H_canariense_414
H_canariense_110
H_thymiphyllum_375
H_thymiphyllum_410
H_pomeridianum_352
H_pomeridianum_144
H_lunulatum_329
H_lunulatum_185
H_caput_felis_121
H_caput_felis_275_R2
H_caput_felis_275_R1
H_sanguineum_295
H_sanguineum_233
H_pannosum_75_5
H_pannosum_75_6
H_viscidulum_viscidulum_319
H_viscidulum_raynaudii_70_2
H_marifolium_conquense_55
H_oelandicum_incanum_72
H_oelandicum_italicum_325
H_oelandicum_pourretii_332
H_oelandicum_alpestris_184_2
H_marifolium_frigidulum_267
H_polyanthum_117
H_polyanthum_109
H_marifolium_andalusicum_266
H_marifolium_molle_251
H_marifolium_origanifolium_234
H_marifolium_marifolium_85
H_cinereum_guadiccianum_64
H_cinereum_cinereum_60
H_cinereum_rotundifolium_72
H_cinereum_hieronymi_62
H_angustatum_66
H_salicifolium_76
H_ledifolium_107
H_papillare_106
H_aegyptiacum_89
H_helianthemoides_426
H_helianthemoides_429
H_obtusifolium_294
H_ruficomum_111
H_nummularium_lycaonicum_269
H_polygonoides_59_R1
H_polygonoides_59_R2
H_grosii_425
H_grosii_118
bro_407A
H_broussonetii_397
H_inaguae_418
H_spNov_2_398
H_spNov_2_432
H_juliae_434
H_cirae_432
H_cirae_400
H_juliae_404
H_aganae_435
H_gonzalezferreri_411
H_spNov_3_437
H_gonzalezferreri_222
H_bramwelliorum_132
H_bramwelliorum_412
H_inaguae_225
H_tholiforme_420
H_teneriffae_401
H_bystropogophyllum_224
H_bystropogophyllum_419
H_linii_399
H_spNov_1_405
H_vesicarium_388
H_sauvagei_282
H_ciliatum_150
H_nummularium_nummularium_300
H_apenninum_stoechadifolium_236
H_apenninum_cantabricum_368
H_raskebdanae_274
H_apenninum_estevei_75
H_morisianum_395
H_alypoides_46
H_marminorense_276
H_almeriense_60
H_apenninum_urrielense_104
H_tinetense_369
H_nummularium_grandiflorum_332
H_nummularium_semiglabrum_327
H_nummularium_obscurum_322
H_kostchianum_268
H_viscarium_62
H_violaceum_317
H_guerrae_61
H_neopiliferum_125
H_apenninum_suffruticosum_12
H_hirtum_55
H_abelardoi_38
H_apenninum_cavanillesianum_64
H_scopulicolum_363
H_apenninum_apenninum_239
H_asperum_65
H_pergamaceum_115
H_virgatum_105
0.004
Sect. Argyrolepis
Sect. Lavandulaceaum
Sect. Pseudomacularia
Sect. Eriocarpum
Subgen. Helianthemum
Sect. Macularia
Sect. Caput-felis
Sect. Atlanthemum
Sect. Pseudocistus
Subgen. Plectolobum
Sect. Brachypetalum
Sect. Helianthemum
Subgen. Helianthemum

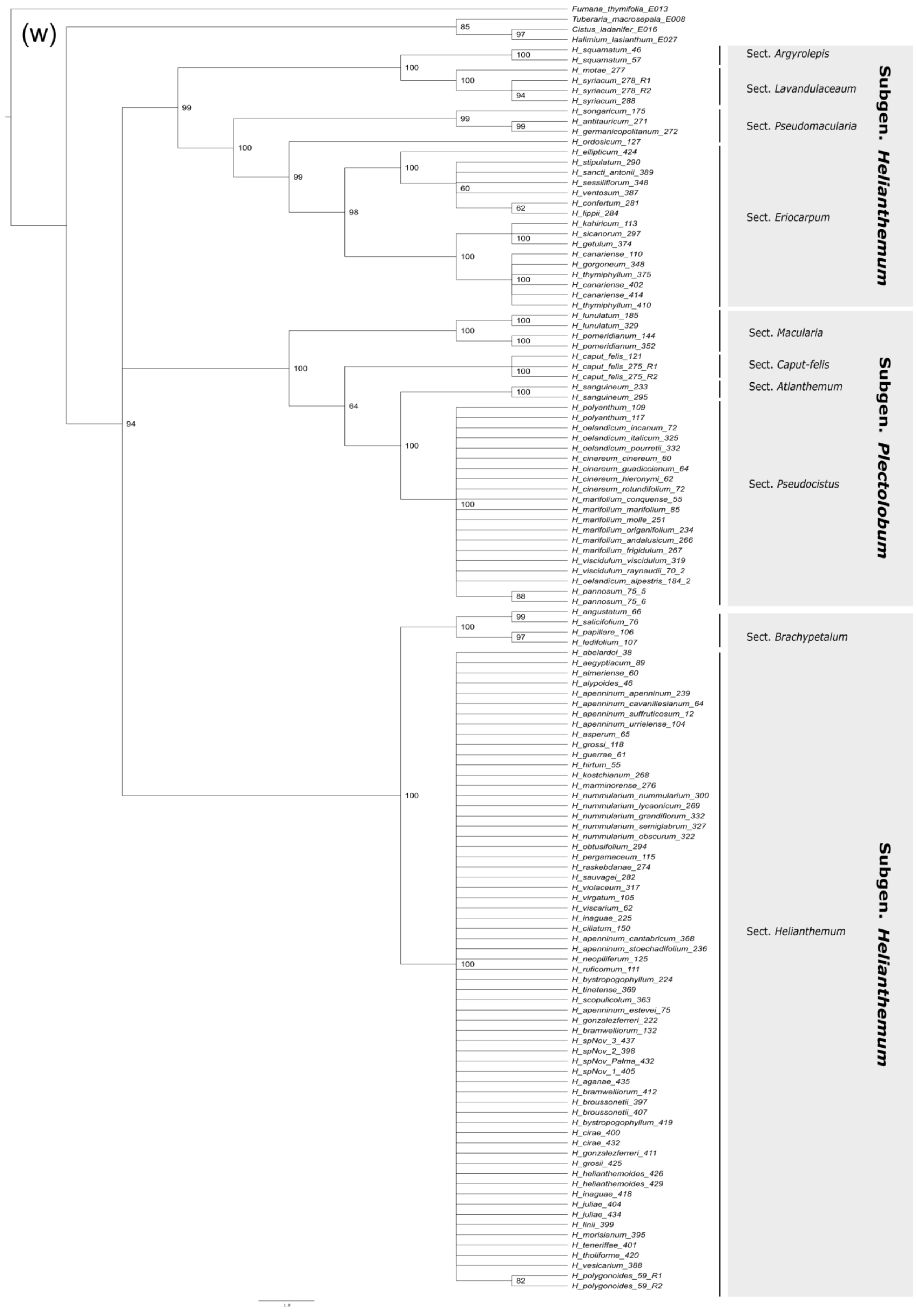

(w)
Fumana_thymifolia_E013
Tuberaria_macrosepala_E008
Cistus_ladanifer_E016
Halimium_lasianthum_E027
H_squamatum_46
H_squamatum_57
H_motae_277
H_syriacum_278_R1
H_syriacum_278_R2
H_syriacum_288
H_songaricum_175
H_antitauricum_271
H_germanicopolitanum_272
H_ordosicum_127
H_ellipticum_424
H_stipulatum_290
H_sancti_antonii_389
H_sessiliflorum_348
H_ventosum_387
H_confertum_281
H_lippii_284
H_kahiricum_113
H_sicanorum_297
H_getulum_374
H_canariense_110
H_gorgoneum_348
H_thymiphyllum_375
H_canariense_402
H_canariense_414
H_thymiphyllum_410
H_lunulatum_185
H_lunulatum_329
H_pomeridianum_144
H_pomeridianum_352
H_caput_felis_121
H_caput_felis_275_R1
H_caput_felis_275_R2
H_sanguineum_233
H_sanguineum_295
H_polyanthum_109
H_polyanthum_117
H_oelandicum_incanum_72
H_oelandicum_italicum_325
H_oelandicum_pourretii_332
H_cinereum_cinereum_60
H_cinereum_guadiccianum_64
H_cinereum_hieronymi_62
H_cinereum_rotundifolium_72
H_marifolium_conquense_55
H_marifolium_marifolium_85
H_marifolium_molle_251
H_marifolium_origanifolium_234
H_marifolium_andalusicum_266
H_marifolium_frigidulum_267
H_viscidulum_viscidulum_319
H_viscidulum_raynaudii_70_2
H_oelandicum_alpestris_184_2
H_pannosum_75_5
H_pannosum_75_6
H_angustatum_66
H_salicifolium_76
H_papillare_106
H_ledifolium_107
H_abelardoi_38
H_aegyptiacum_89
H_almeriense_60
H_alypoides_46
H_apenninum_apenninum_239
H_apenninum_cavanillesianum_64
H_apenninum_suffruticosum_12
H_apenninum_urrielense_104
H_asperum_65
H_grossi_118
H_guerrae_61
H_hirtum_55
H_kostchianum_268
H_marminorense_276
H_nummularium_nummularium_300
H_nummularium_lycaonicum_269
H_nummularium_grandiflorum_332
H_nummularium_semiglabrum_327
H_nummularium_obscurum_322
H_obtusifolium_294
H_pergamaceum_115
H_raskebdanae_274
H_sauvagei_282
H_violaceum_317
H_virgatum_105
H_viscarium_62
H_inaguae_225
H_ciliatum_150
H_apenninum_cantabricum_368
H_apenninum_stoechadifolium_236
H_neopiliferum_125
H_ruficomum_111
H_bystropogophyllum_224
H_tinetense_369
H_scopulicolum_363
H_apenninum_estevei_75
H_gonzalezferreri_222
H_bramwelliorum_132
H_spNov_3_437
H_spNov_2_398
H_spNov_Palma_432
H_spNov_1_405
H_aganae_435
H_bramwelliorum_412
H_broussonetii_397
H_broussonetii_407
H_bystropogophyllum_419
H_cirae_400
H_cirae_432
H_gonzalezferreri_411
H_grosii_425
H_helianthemoides_426
H_helianthemoides_429
H_inaguae_418
H_juliae_404
H_juliae_434
H_linii_399
H_morisianum_395
H_teneriffae_401
H_tholiforme_420
H_vesicarium_388
H_polygonoides_59_R1
H_polygonoides_59_R2
Sect. Argyrolepis
Sect. Lavandulaceaum
Sect. Pseudomacularia
Sect. Eriocarpum
Subgen. Helianthemum
Sect. Macularia
Sect. Caput-felis
Sect. Atlanthemum
Sect. Pseudocistus
Subgen. Plectolobum
Sect. Brachypetalum
Sect. Helianthemum
Subgen. Helianthemum

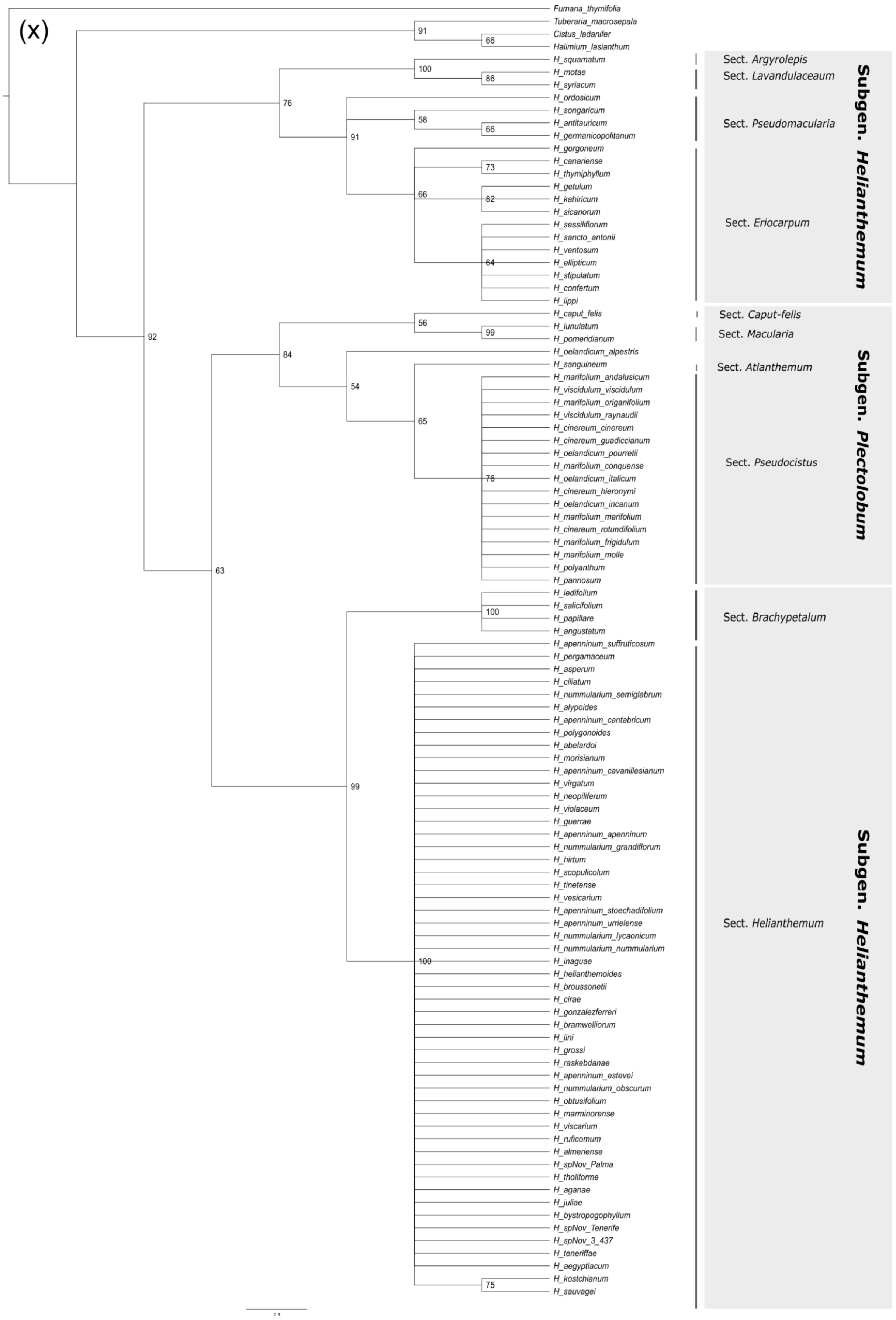
(x)
Fumana_thymifolia
Tuberaria_macrosepala
Cistus_ladanifer
Halimium_lasianthum
H_squamatum
H_motae
H_syriacum
H_ordosicum
H_songaricum
H_antitauricum
H_germanicopolitanum
H_gorgoneum
H_canariense
H_thymiphyllum
H_getulum
H_kahiricum
H_sicanorum
H_sessiliflorum
H_sancto_antonii
H_ventosum
H_ellipticum
H_stipulatum
H_confertum
H_lippi
H_caput_felis
H_lunulatum
H_pomeridianum
H_oelandicum_alpestris
H_sanguineum
H_marifolium_andalusicum
H_viscidulum_viscidulum
H_marifolium_origanifolium
H_viscidulum_raynaudii
H_cinereum_cinereum
H_cinereum_guadiccianum
H_oelandicum_pourretii
H_marifolium_conquense
H_oelandicum_italicum
H_cinereum_hieronymi
H_oelandicum_incanum
H_marifolium_marifolium
H_cinereum_rotundifolium
H_marifolium_frigidulum
H_marifolium_molle
H_polyanthum
H_pannosum
H_ledifolium
H_salicifolium
H_papillare
H_angustatum
H_apenninum_suffruticosum
H_pergamaceum
H_asperum
H_ciliatum
H_nummularium_semiglabrum
H_alypoides
H_apenninum_cantabricum
H_polygonoides
H_abelardoi
H_morisianum
H_apenninum_cavanillesianum
H_virgatum
H_neopiliferum
H_violaceum
H_guerrae
H_apenninum_apenninum
H_nummularium_grandiflorum
H_hirtum
H_scopulicolum
H_tinetense
H_vesicarium
H_apenninum_stoechadifolium
H_apenninum_urrielense
H_nummularium_lycaonicum
H_nummularium_nummularium
H_inaguae
H_helianthemoides
H_broussonetii
H_cirae
H_gonzalezferreri
H_bramwelliorum
H_lini
H_grossi
H_raskebdanae
H_apenninum_estevei
H_nummularium_obscurum
H_obtusifolium
H_marminorense
H_viscarium
H_ruficomum
H_almeriense
H_spNov_Palma
H_tholiforme
H_aganae
H_juliae
H_bystropogophyllum
H_spNov_Tenerife
H_spNov_3_437
H_teneriffae
H_aegyptiacum
H_kostchianum
H_sauvagei
Sect. Argyrolepis
Sect. Lavandulaceaum
Sect. Pseudomacularia
Sect. Eriocarpum
Subgen. Helianthemum
Sect. Caput-felis
Sect. Macularia
Sect. Atlanthemum
Sect. Pseudocistus
Subgen. Plectolobum
Sect. Brachypetalum
Sect. Helianthemum
Subgen. Helianthemum

Figure S2 Chronograms obtained in TreePL using MaxResol and MinError assemblies under three minimum taxon coverage percentages (MinCov 15%, 25% and 50%). A total of 126 samples of the genus *Helianthemum* are included. Numbers at nodes are mean ages obtained by TreePL analyses of the 50% majority-rule consensus tree from the ExaBayes analyses. Node bars represent age uncertainty (95% probability intervals) resulting from TreePL analyses of 900 trees from the Bayesian distribution obtained in ExaBayes. Diamonds indicate calibration points (see text for details).

(a) TreePL chronogram from MaxResol configuration, MinCov 15% assembly
(b) TreePL chronogram from MaxResol configuration, MinCov 25% assembly
(c) TreePL chronogram from MaxResol configuration, MinCov 50% assembly
(d) TreePL chronogram from MinError configuration, MinCov 15% assembly
(e) TreePL chronogram from MinError configuration, MinCov 25% assembly

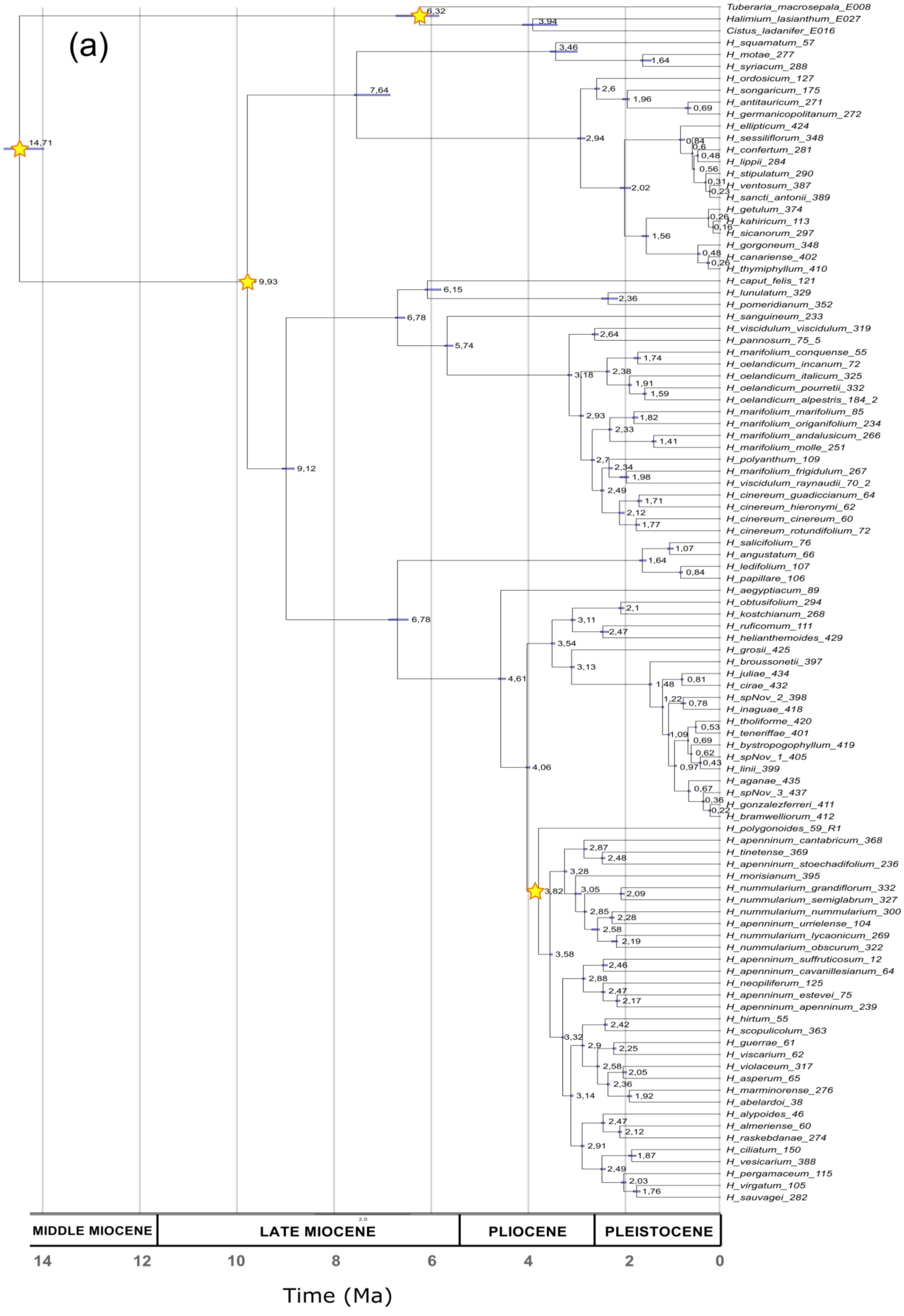
(a)
Tuberaria_macrosepala_E008
Halimium_lasianthum_E027
Cistus_ladanifer_E016
H_squamatum_57
H_motae_277
H_syriacum_288
H_ordosicum_127
H_songaricum_175
H_antitauricum_271
H_germanicopolitanum_272
H_ellipticum_424
H_sessiliflorum_348
H_confertum_281
H_lippii_284
H_stipulatum_290
H_ventosum_387
H_sancti_antonii_389
H_getulum_374
H_kahiricum_113
H_sicanorum_297
H_gorgoneum_348
H_canariense_402
H_thymiphyllum_410
H_caput_felis_121
H_lunulatum_329
H_pomeridianum_352
H_sanguineum_233
H_viscidulum_viscidulum_319
H_pannosum_75_5
H_marifolium_conquense_55
H_oelandicum_incanum_72
H_oelandicum_italicum_325
H_oelandicum_pourretii_332
H_oelandicum_alpestris_184_2
H_marifolium_marifolium_85
H_marifolium_origanifolium_234
H_marifolium_andalusicum_266
H_marifolium_molle_251
H_polyanthum_109
H_marifolium_frigidulum_267
H_viscidulum_raynaudii_70_2
H_cinereum_guadiccianum_64
H_cinereum_hieronymi_62
H_cinereum_cinereum_60
H_cinereum_rotundifolium_72
H_salicifolium_76
H_angustatum_66
H_ledifolium_107
H_papillare_106
H_aegyptiacum_89
H_obtusifolium_294
H_kostchianum_268
H_ruficomum_111
H_helianthemoides_429
H_grosii_425
H_broussonetii_397
H_juliae_434
H_cirae_432
H_spNov_2_398
H_inaguae_418
H_tholiforme_420
H_teneriffae_401
H_bystropogophyllum_419
H_spNov_1_405
H_linii_399
H_aganae_435
H_spNov_3_437
H_gonzalezferreri_411
H_bramwelliorum_412
H_polygonoides_59_R1
H_apenninum_cantabricum_368
H_tinetense_369
H_apenninum_stoechadifolium_236
H_morisianum_395
H_nummularium_grandiflorum_332
H_nummularium_semiglabrum_327
H_nummularium_nummularium_300
H_apenninum_urrielense_104
H_nummularium_lycaonicum_269
H_nummularium_obscurum_322
H_apenninum_suffruticosum_12
H_apenninum_cavanillesianum_64
H_neopiliferum_125
H_apenninum_estevei_75
H_apenninum_apenninum_239
H_hirtum_55
H_scopulicolum_363
H_guerrae_61
H_viscarium_62
H_violaceum_317
H_asperum_65
H_marminorense_276
H_abelardoi_38
H_alypoides_46
H_almeriense_60
H_raskebdanae_274
H_ciliatum_150
H_vesicarium_388
H_pergamaceum_115
H_virgatum_105
H_sauvagei_282
MIDDLE MIOCENE
LATE MIOCENE
PLIOCENE
PLEISTOCENE
14
12
10
8
6
4
2
0
Time (Ma)

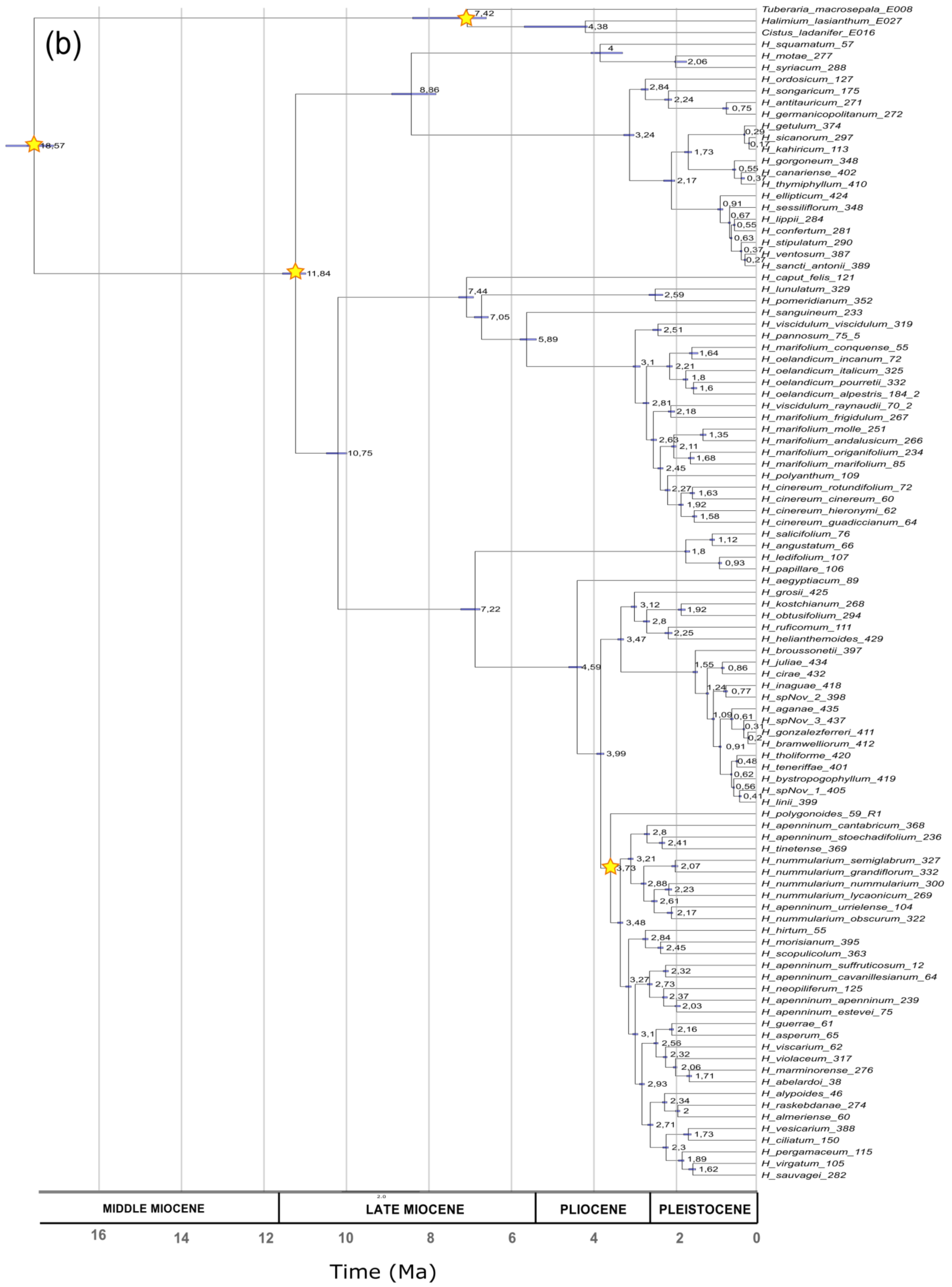
(b)
Tuberaria_macrosepala_E008
Halimium_lasianthum_E027
Cistus_ladanifer_E016
H_squamatum_57
H_motae_277
H_syriacum_288
H_ordosicum_127
H_songaricum_175
H_antitauricum_271
H_germanicopolitanum_272
H_getulum_374
H_sicanorum_297
H_kahiricum_113
H_gorgoneum_348
H_canariense_402
H_thymiphyllum_410
H_ellipticum_424
H_sessiliflorum_348
H_lippii_284
H_confertum_281
H_stipulatum_290
H_ventosum_387
H_sancti_antonii_389
H_caput_felis_121
H_lunulatum_329
H_pomeridianum_352
H_sanguineum_233
H_viscidulum_viscidulum_319
H_pannosum_75_5
H_marifolium_conquense_55
H_oelandicum_incanum_72
H_oelandicum_italicum_325
H_oelandicum_pourretii_332
H_oelandicum_alpestris_184_2
H_viscidulum_raynaudii_70_2
H_marifolium_frigidulum_267
H_marifolium_molle_251
H_marifolium_andalusicum_266
H_marifolium_origanifolium_234
H_marifolium_marifolium_85
H_polyanthum_109
H_cinereum_rotundifolium_72
H_cinereum_cinereum_60
H_cinereum_hieronymi_62
H_cinereum_guadiccianum_64
H_salicifolium_76
H_angustatum_66
H_ledifolium_107
H_papillare_106
H_aegyptiacum_89
H_grosii_425
H_kostchianum_268
H_obtusifolium_294
H_ruficomum_111
H_helianthemoides_429
H_broussonetii_397
H_juliae_434
H_cirae_432
H_inaguae_418
H_spNov_2_398
H_aganae_435
H_spNov_3_437
H_gonzalezferreri_411
H_bramwelliorum_412
H_tholiforme_420
H_teneriffae_401
H_bystropogophyllum_419
H_spNov_1_405
H_linii_399
H_polygonoides_59_R1
H_apenninum_cantabricum_368
H_apenninum_stoechadifolium_236
H_tinetense_369
H_nummularium_semiglabrum_327
H_nummularium_grandiflorum_332
H_nummularium_nummularium_300
H_nummularium_lycaonicum_269
H_apenninum_urrielense_104
H_nummularium_obscurum_322
H_hirtum_55
H_morisianum_395
H_scopulicolum_363
H_apenninum_suffruticosum_12
H_apenninum_cavanillesianum_64
H_neopiliferum_125
H_apenninum_apenninum_239
H_apenninum_estevei_75
H_guerrae_61
H_asperum_65
H_viscarium_62
H_violaceum_317
H_marminorense_276
H_abelardoi_38
H_alypoides_46
H_raskebdanae_274
H_almeriense_60
H_vesicarium_388
H_ciliatum_150
H_pergamaceum_115
H_virgatum_105
H_sauvagei_282
MIDDLE MIOCENE
LATE MIOCENE
PLIOCENE
PLEISTOCENE
16
14
12
10
8
6
4
2
0
Time (Ma)

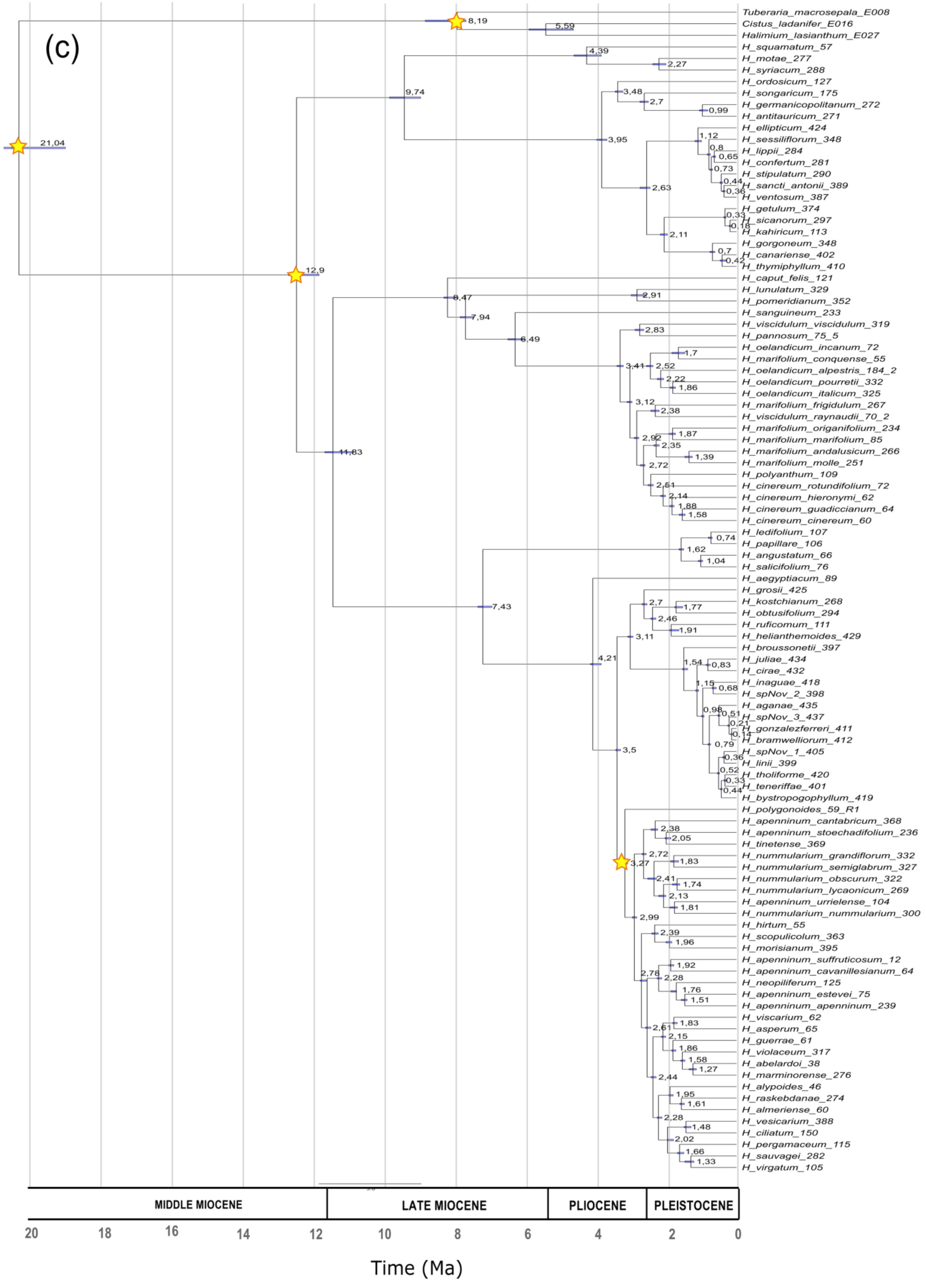
(c)
Tuberaria_macrosepala_E008
Cistus_ladanifer_E016
Halimium_lasianthum_E027
H_squamatum_57
H_motae_277
H_syriacum_288
H_ordosicum_127
H_songaricum_175
H_germanicopolitanum_272
H_antitauricum_271
H_ellipticum_424
H_sessiliflorum_348
H_lippii_284
H_confertum_281
H_stipulatum_290
H_sancti_antonii_389
H_ventosum_387
H_getulum_374
H_sicanorum_297
H_kahiricum_113
H_gorgoneum_348
H_canariense_402
H_thymiphyllum_410
H_caput_felis_121
H_lunulatum_329
H_pomeridianum_352
H_sanguineum_233
H_viscidulum_viscidulum_319
H_pannosum_75_5
H_oelandicum_incanum_72
H_marifolium_conquense_55
H_oelandicum_alpestris_184_2
H_oelandicum_pourretii_332
H_oelandicum_italicum_325
H_marifolium_frigidulum_267
H_viscidulum_raynaudii_70_2
H_marifolium_origanifolium_234
H_marifolium_marifolium_85
H_marifolium_andalusicum_266
H_marifolium_molle_251
H_polyanthum_109
H_cinereum_rotundifolium_72
H_cinereum_hieronymi_62
H_cinereum_guadiccianum_64
H_cinereum_cinereum_60
H_ledifolium_107
H_papillare_106
H_angustatum_66
H_salicifolium_76
H_aegyptiacum_89
H_grosii_425
H_kostchianum_268
H_obtusifolium_294
H_ruficomum_111
H_helianthemoides_429
H_broussonetii_397
H_juliae_434
H_cirae_432
H_inaguae_418
H_spNov_2_398
H_aganae_435
H_spNov_3_437
H_gonzalezferreri_411
H_bramwelliorum_412
H_spNov_1_405
H_linii_399
H_tholiforme_420
H_teneriffae_401
H_bystropogophyllum_419
H_polygonoides_59_R1
H_apenninum_cantabricum_368
H_apenninum_stoechadifolium_236
H_tinetense_369
H_nummularium_grandiflorum_332
H_nummularium_semiglabrum_327
H_nummularium_obscurum_322
H_nummularium_lycaonicum_269
H_apenninum_urrielense_104
H_nummularium_nummularium_300
H_hirtum_55
H_scopulicolum_363
H_morisianum_395
H_apenninum_suffruticosum_12
H_apenninum_cavanillesianum_64
H_neopiliferum_125
H_apenninum_estevei_75
H_apenninum_apenninum_239
H_viscarium_62
H_asperum_65
H_guerrae_61
H_violaceum_317
H_abelardoi_38
H_marminorense_276
H_alypoides_46
H_raskebdanae_274
H_almeriense_60
H_vesicarium_388
H_ciliatum_150
H_pergamaceum_115
H_sauvagei_282
H_virgatum_105
MIDDLE MIOCENE
LATE MIOCENE
PLIOCENE
PLEISTOCENE
20
18
16
14
12
10
8
6
4
2
0
Time (Ma)

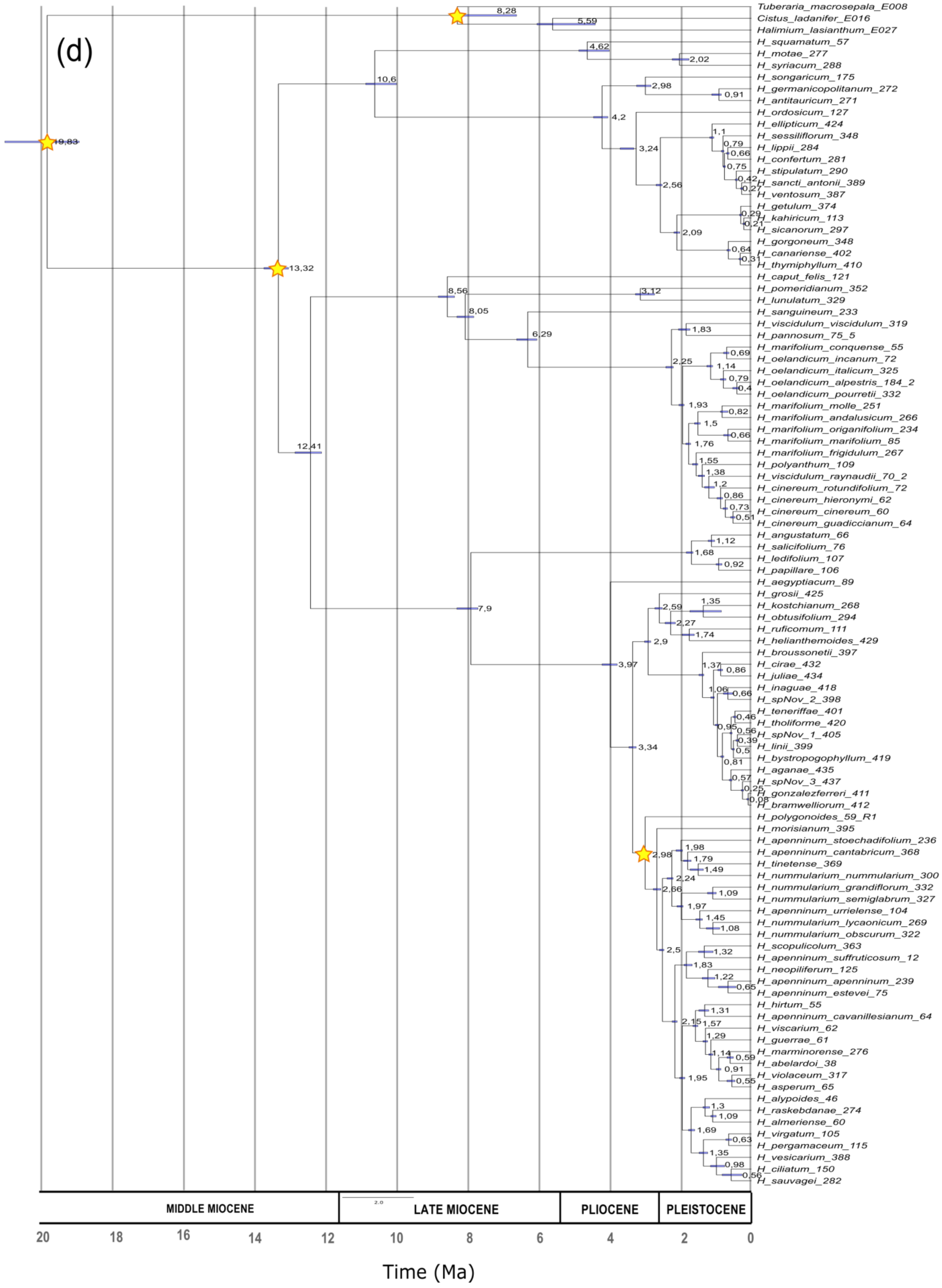

(d)
Tuberaria_macrosepala_E008
Cistus_ladanifer_E016
Halimium_lasianthum_E027
H_squamatum_57
H_motae_277
H_syriacum_288
H_songaricum_175
H_germanicopolitanum_272
H_antitauricum_271
H_ordosicum_127
H_ellipticum_424
H_sessiliflorum_348
H_lippii_284
H_confertum_281
H_stipulatum_290
H_sancti_antonii_389
H_ventosum_387
H_getulum_374
H_kahiricum_113
H_sicanorum_297
H_gorgoneum_348
H_canariense_402
H_thymiphyllum_410
H_caput_felis_121
H_pomeridianum_352
H_lunulatum_329
H_sanguineum_233
H_viscidulum_viscidulum_319
H_pannosum_75_5
H_marifolium_conquense_55
H_oelandicum_incanum_72
H_oelandicum_italicum_325
H_oelandicum_alpestris_184_2
H_oelandicum_pourretii_332
H_marifolium_molle_251
H_marifolium_andalusicum_266
H_marifolium_origanifolium_234
H_marifolium_marifolium_85
H_marifolium_frigidulum_267
H_polyanthum_109
H_viscidulum_raynaudii_70_2
H_cinereum_rotundifolium_72
H_cinereum_hieronymi_62
H_cinereum_cinereum_60
H_cinereum_guadiccianum_64
H_angustatum_66
H_salicifolium_76
H_ledifolium_107
H_papillare_106
H_aegyptiacum_89
H_grosii_425
H_kostchianum_268
H_obtusifolium_294
H_ruficomum_111
H_helianthemoides_429
H_broussonetii_397
H_cirae_432
H_juliae_434
H_inaguae_418
H_spNov_2_398
H_teneriffae_401
H_tholiforme_420
H_spNov_1_405
H_linii_399
H_bystropogophyllum_419
H_aganae_435
H_spNov_3_437
H_gonzalezferreri_411
H_bramwelliorum_412
H_polygonoides_59_R1
H_morisianum_395
H_apenninum_stoechadifolium_236
H_apenninum_cantabricum_368
H_tinetense_369
H_nummularium_nummularium_300
H_nummularium_grandiflorum_332
H_nummularium_semiglabrum_327
H_apenninum_urrielense_104
H_nummularium_lycaonicum_269
H_nummularium_obscurum_322
H_scopulicolum_363
H_apenninum_suffruticosum_12
H_neopiliferum_125
H_apenninum_apenninum_239
H_apenninum_estevei_75
H_hirtum_55
H_apenninum_cavanillesianum_64
H_viscarium_62
H_guerrae_61
H_marminorense_276
H_abelardoi_38
H_violaceum_317
H_asperum_65
H_alypoides_46
H_raskebdanae_274
H_almeriense_60
H_virgatum_105
H_pergamaceum_115
H_vesicarium_388
H_ciliatum_150
H_sauvagei_282
MIDDLE MIOCENE
LATE MIOCENE
PLIOCENE
PLEISTOCENE
20
18
16
14
12
10
8
6
4
2
0
Time (Ma)

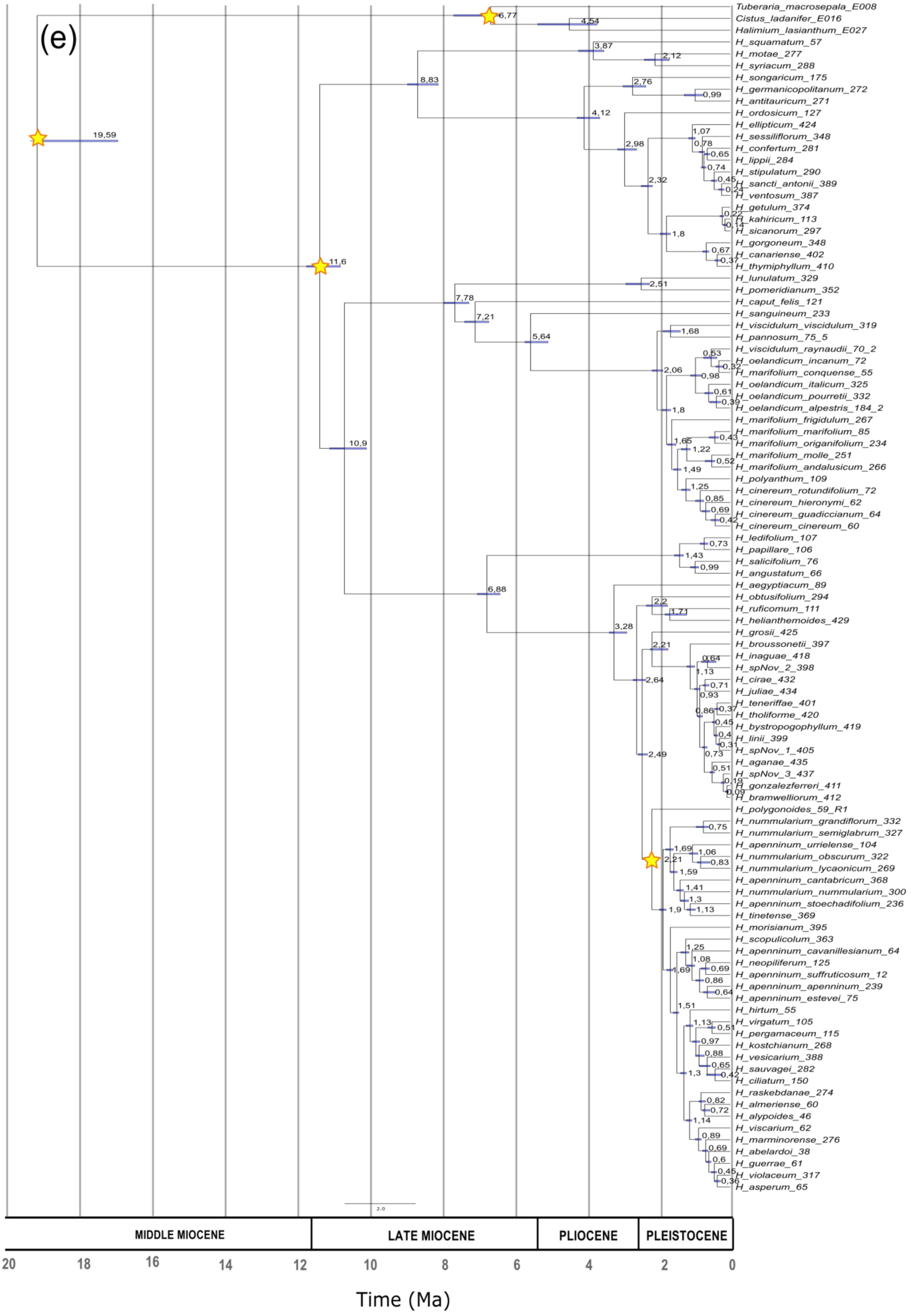

(e)
Tuberaria_macrosepala_E008
Cistus_ladanifer_E016
Halimium_lasianthum_E027
H_squamatum_57
H_motae_277
H_syriacum_288
H_songaricum_175
H_germanicopolitanum_272
H_antitauricum_271
H_ordosicum_127
H_ellipticum_424
H_sessiliflorum_348
H_confertum_281
H_lippii_284
H_stipulatum_290
H_sancti_antonii_389
H_ventosum_387
H_getulum_374
H_kahiricum_113
H_sicanorum_297
H_gorgoneum_348
H_canariense_402
H_thymiphyllum_410
H_lunulatum_329
H_pomeridianum_352
H_caput_felis_121
H_sanguineum_233
H_viscidulum_viscidulum_319
H_pannosum_75_5
H_viscidulum_raynaudii_70_2
H_oelandicum_incanum_72
H_marifolium_conquense_55
H_oelandicum_italicum_325
H_oelandicum_pourretii_332
H_oelandicum_alpestris_184_2
H_marifolium_frigidulum_267
H_marifolium_marifolium_85
H_marifolium_origanifolium_234
H_marifolium_molle_251
H_marifolium_andalusicum_266
H_polyanthum_109
H_cinereum_rotundifolium_72
H_cinereum_hieronymi_62
H_cinereum_guadiccianum_64
H_cinereum_cinereum_60
H_ledifolium_107
H_papillare_106
H_salicifolium_76
H_angustatum_66
H_aegyptiacum_89
H_obtusifolium_294
H_ruficomum_111
H_helianthemoides_429
H_grosii_425
H_broussonetii_397
H_inaguae_418
H_spNov_2_398
H_cirae_432
H_juliae_434
H_teneriffae_401
H_tholiforme_420
H_bystropogophyllum_419
H_linii_399
H_spNov_1_405
H_aganae_435
H_spNov_3_437
H_gonzalezferreri_411
H_bramwelliorum_412
H_polygonoides_59_R1
H_nummularium_grandiflorum_332
H_nummularium_semiglabrum_327
H_apenninum_urrielense_104
H_nummularium_obscurum_322
H_nummularium_lycaonicum_269
H_apenninum_cantabricum_368
H_nummularium_nummularium_300
H_apenninum_stoechadifolium_236
H_tinetense_369
H_morisianum_395
H_scopulicolum_363
H_apenninum_cavanillesianum_64
H_neopiliferum_125
H_apenninum_suffruticosum_12
H_apenninum_apenninum_239
H_apenninum_estevei_75
H_hirtum_55
H_virgatum_105
H_pergamaceum_115
H_kostchianum_268
H_vesicarium_388
H_sauvagei_282
H_ciliatum_150
H_raskebdanae_274
H_almeriense_60
H_alypoides_46
H_viscarium_62
H_marminorense_276
H_abelardoi_38
H_guerrae_61
H_violaceum_317
H_asperum_65
MIDDLE MIOCENE
LATE MIOCENE
PLIOCENE
PLEISTOCENE
20
18
16
14
12
10
8
6
4
2
0
Time (Ma)

Figure S3 Diversification rate shift configurations with the highest posterior probabilities, as estimated by BAMM analyses of *Helianthemum* GBS phylogenetic trees. Branches are coloured according to mean net diversification rates resulting from BAMM analyses of the TreePL chronograms obtained from MaxResol and MinError assemblies under three minimum taxon coverage percentages (MinCov 15%, 25% and 50%). A total of 126 samples of the genus *Helianthemum* are included. Red circles mark diversification rate shifts and the size of the circle is proportional to the marginal shift probability. The text above each phylorate plot ("f") gives the posterior probability of each shift configuration.

(a) BAMM diversification rate shift configuration from MaxResol configuration, MinCov 15% assembly
(b) BAMM diversification rate shift configuration from MaxResol configuration, MinCov 25% assembly
(c) BAMM diversification rate shift configuration from MaxResol configuration, MinCov 50% assembly
(d) BAMM diversification rate shift configuration from MinError configuration, MinCov 15% assembly
(e) BAMM diversification rate shift configuration from MinError configuration, MinCov 25% assembly

(a)

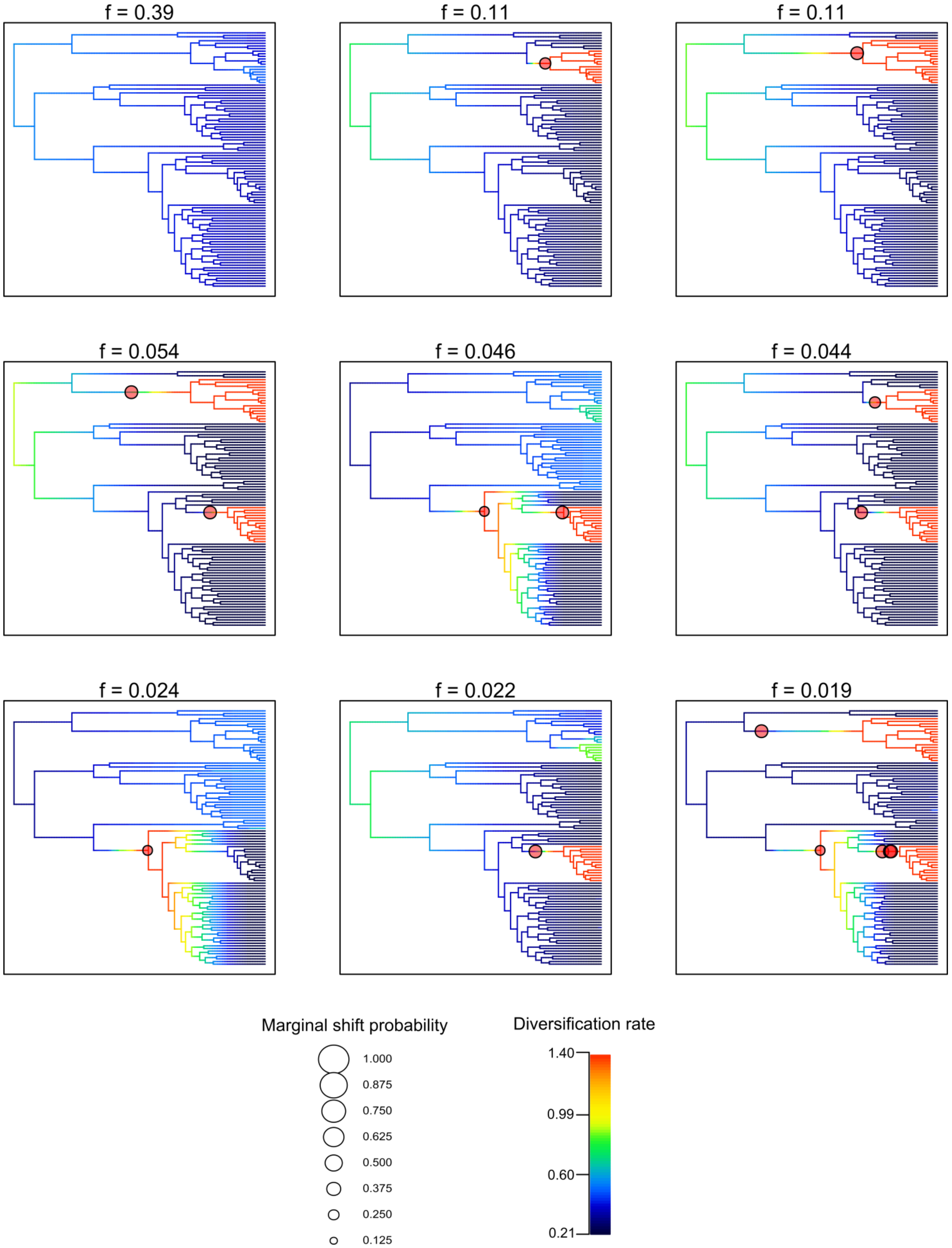
f = 0.39
f = 0.11
f = 0.11
f = 0.054
f = 0.046
f = 0.044
f = 0.024
f = 0.022
f = 0.019
Marginal shift probability
1.000
0.875
0.750
0.625
0.500
0.375
0.250
0.125
Diversification rate
1.40
0.99
0.60
0.21

(b)

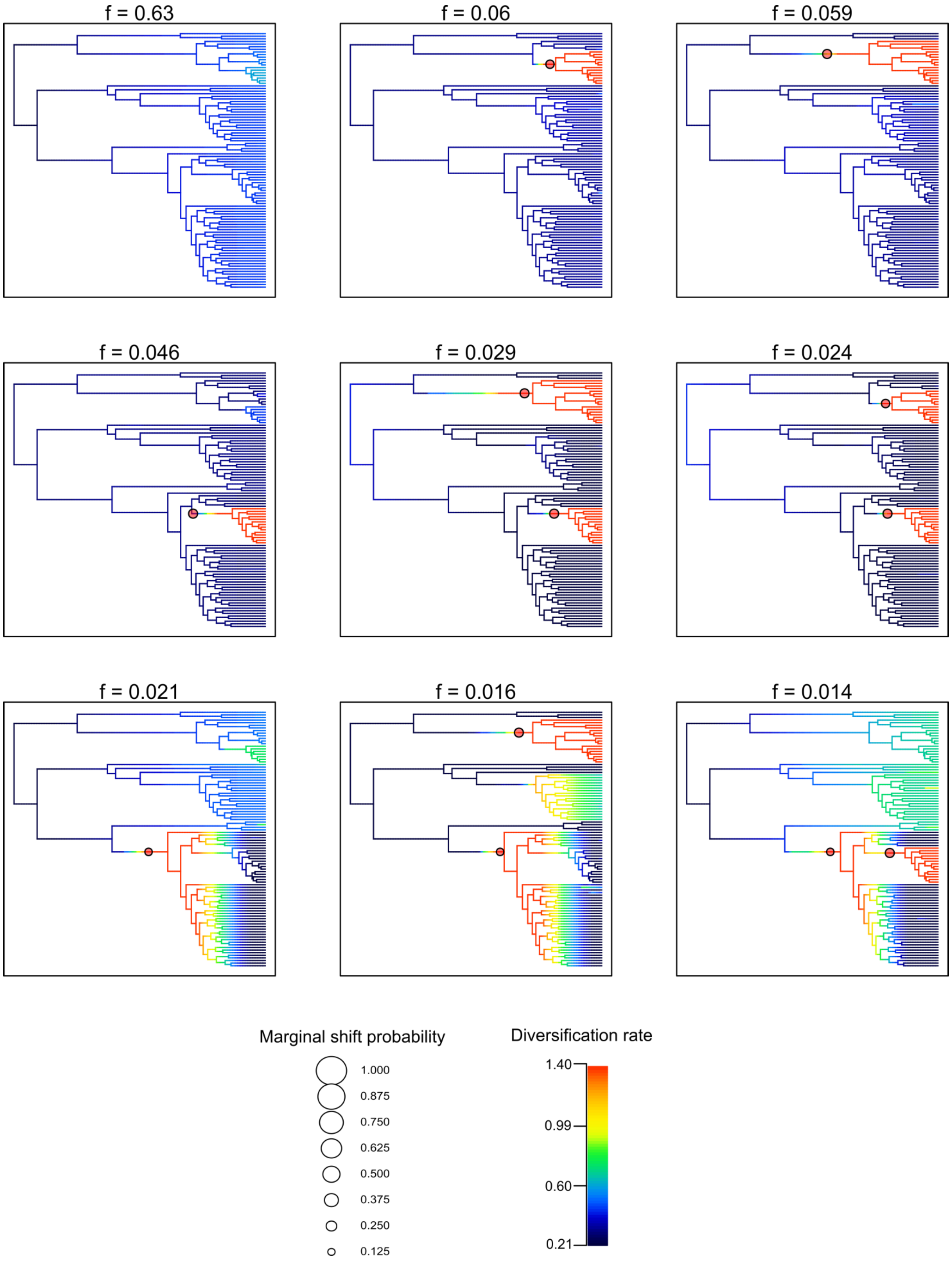
f = 0.63
f = 0.06
f = 0.059
f = 0.046
f = 0.029
f = 0.024
f = 0.021
f = 0.016
f = 0.014
Marginal shift probability
1.000
0.875
0.750
0.625
0.500
0.375
0.250
0.125
Diversification rate
1.40
0.99
0.60
0.21

(c)

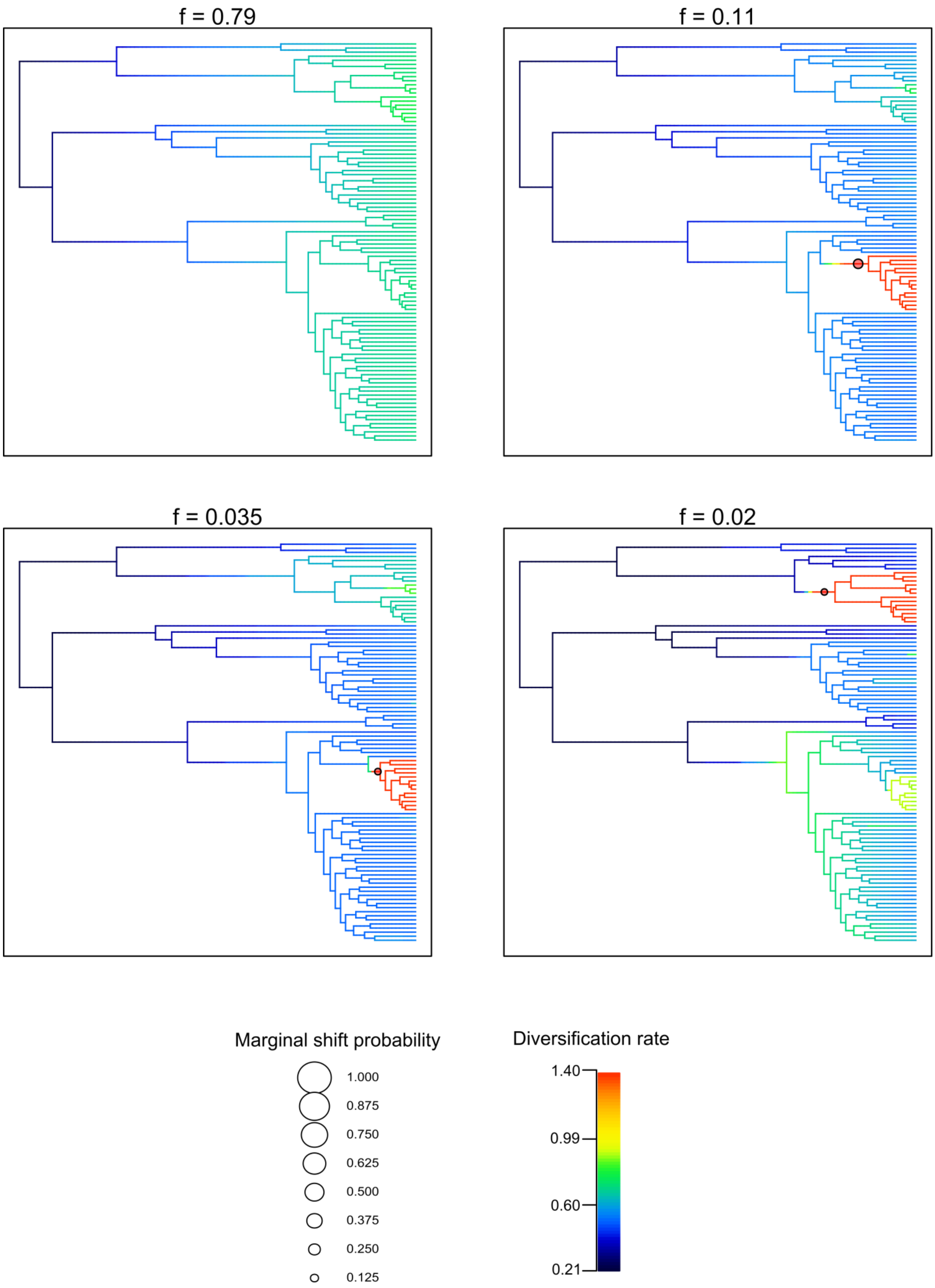
f = 0.79
f = 0.11
f = 0.035
f = 0.02
Marginal shift probability
1.000
0.875
0.750
0.625
0.500
0.375
0.250
0.125
Diversification rate
1.40
0.99
0.60
0.21

(d)

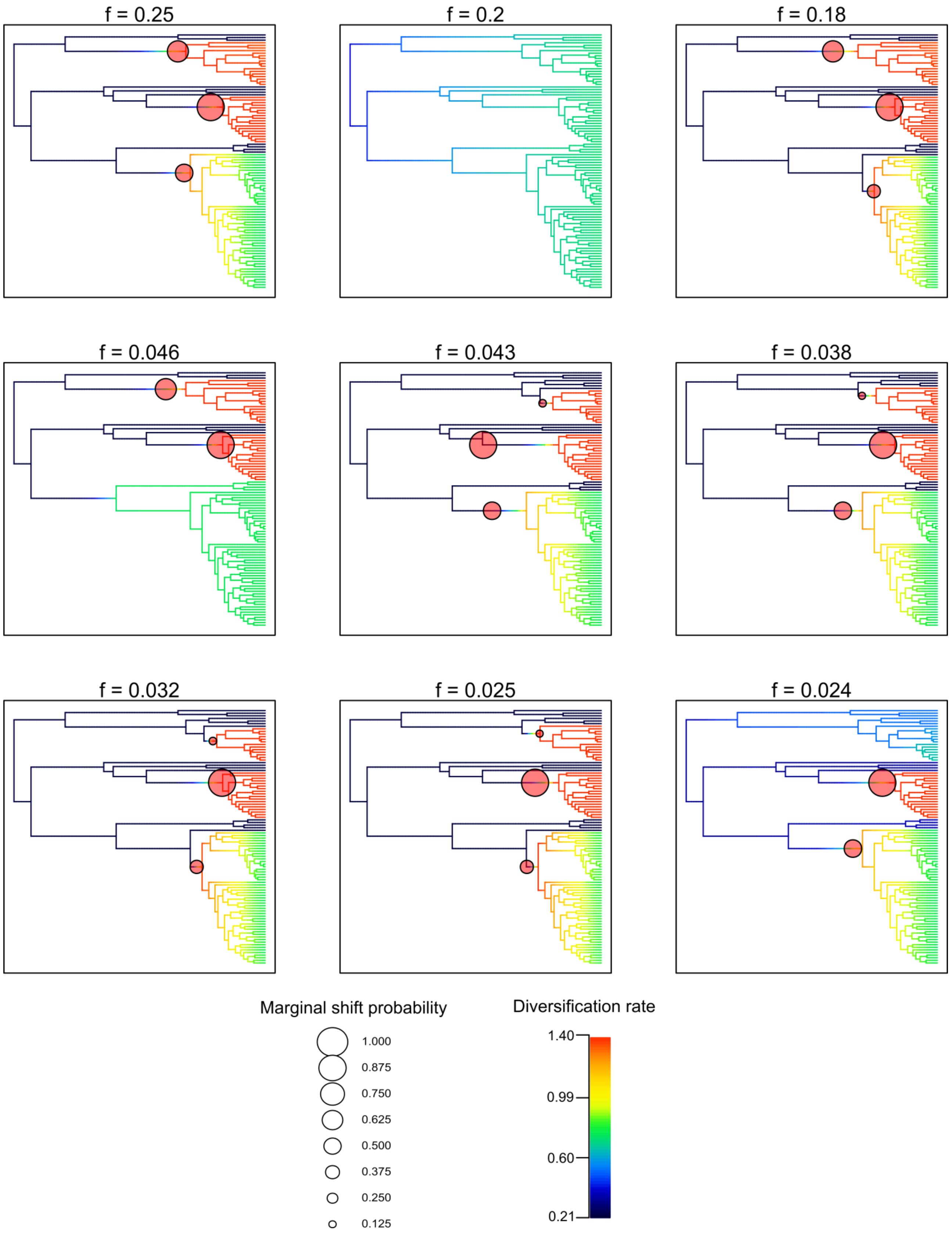
f = 0.25
f = 0.2
f = 0.18
f = 0.046
f = 0.043
f = 0.038
f = 0.032
f = 0.025
f = 0.024
Marginal shift probability
1.000
0.875
0.750
0.625
0.500
0.375
0.250
0.125
Diversification rate
1.40
0.99
0.60
0.21

(e)

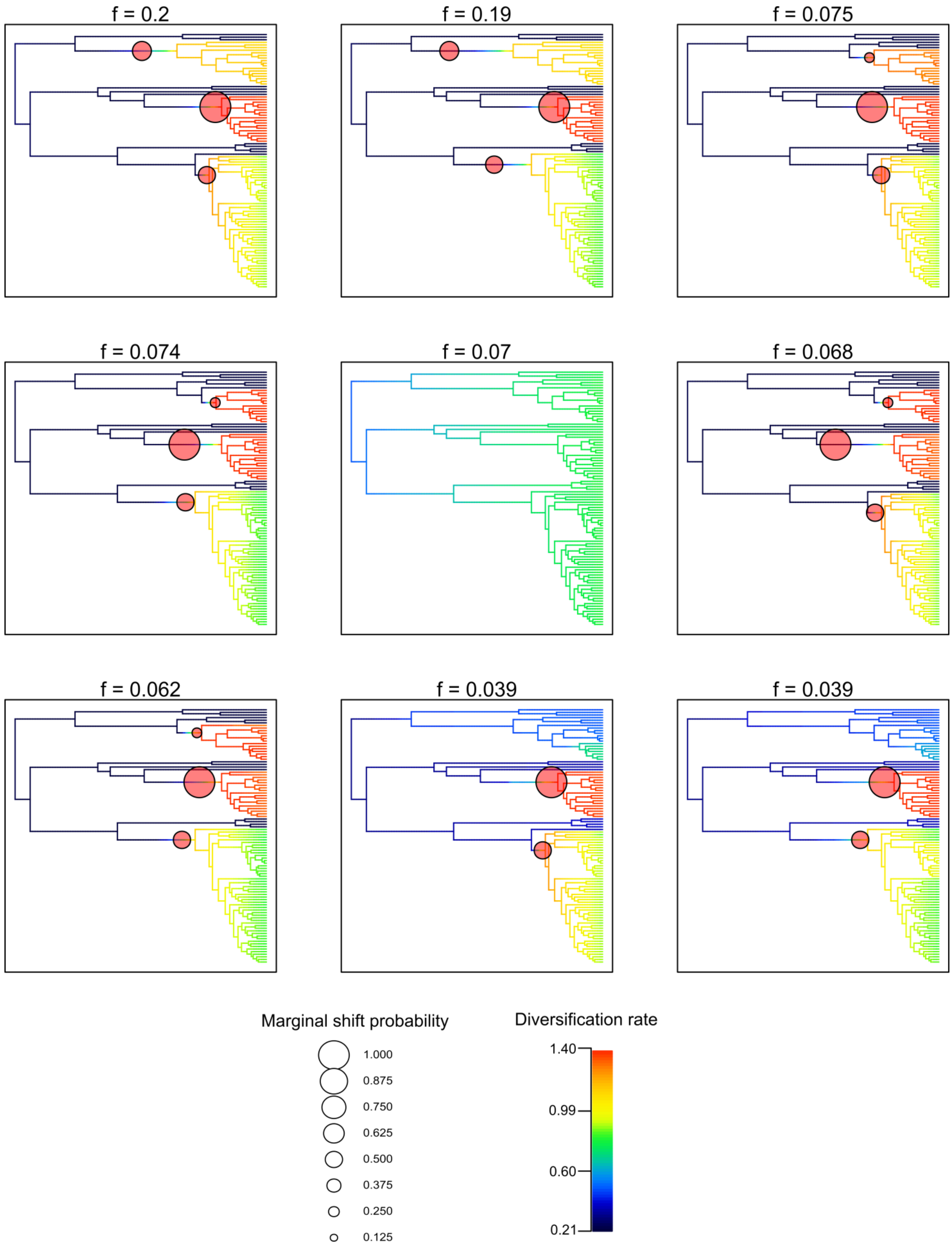
f = 0.2
f = 0.19
f = 0.075
f = 0.074
f = 0.07
f = 0.068
f = 0.062
f = 0.039
f = 0.039
Marginal shift probability
1.000
0.875
0.750
0.625
0.500
0.375
0.250
0.125
Diversification rate
1.40
0.99
0.60
0.21

Figure S4 Comparison of divergence times and diversification patterns recovered from MaxResol and MinError configurations under 15% minimum taxon coverage. Branch colours represent the rate of speciation across the tree estimated using Bayesian Analysis of Macroevolutionary Mixtures (BAMM; Rabosky, 2014). Diversification rate shifts are marked by red circles. Diversification rate scale in species per million years.

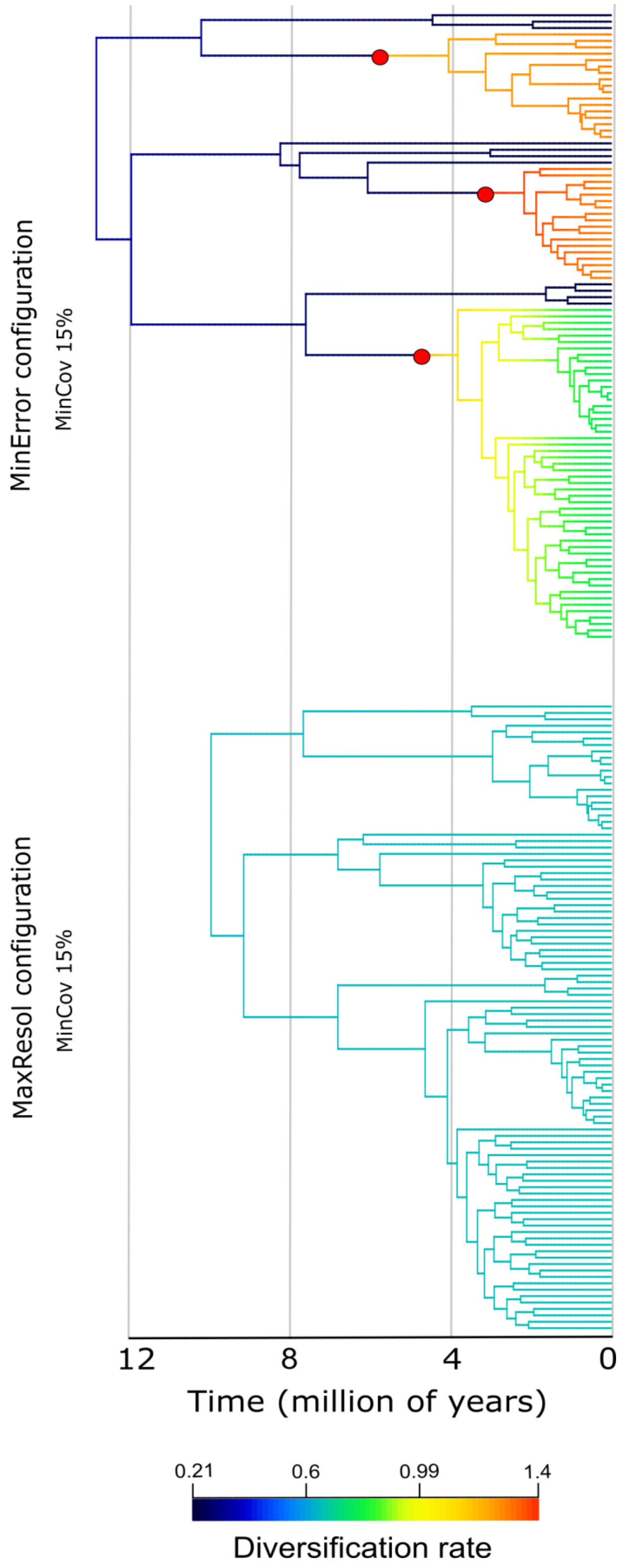

Table S1 Sources of error and bias in phylogenomic inference associated with the use of GBS (Genotyping-by-sequencing) and RADseq (Restriction site-associated DNA sequencing) data. References: Eaton, 2014; Mastretta-Yanes et al., 2015; Andrews et al., 2016; Bleidorn, 2017; Clark et al., 1992; Lemmon et al., 2009; Roure et al., 2012; Jiang et al., 2014; Kuhner & McHill, 2014; Darriba et al., 2016; Eaton et al., 2017.

PHASE OF THE STUDY	TYPE OF ERROR OR BIAS	MEANING	CONSECUENCES
LABORATORY (from DNA extraction until sequencing)	Allele dropout because of mutations at a restriction enzyme recognition site	When a polymorphism occurs at a restriction enzyme recognition site, resulting in a failure to cut the genomic DNA at that location. Alleles that lack the complete recognition site will not be sequenced and are therefore null alleles	Genotyping errors (individuals heterozygous for the null allele appearing as homozygotes)<br><br>Missing data
	PCR duplicates	Stochastic processes cause one allele to amplify more than the other allele at a given locus in an individual sample	Downstream genotyping errors because heterozygotes can appear as homozygotes
	Variance in depth of coverage among loci	Preferential sequencing of certain loci over other loci	Missing data
ASSEMBLY AND BIOINFORMATIC PROCESSING	Non-optimized values of minimum sample coverage	Setting a threshold for minimal coverage (defined by researchers) allows to distinguish between PCR/sequencing error and real variation	Too low values: Genotyping errors (error-based variation are considered real)<br><br>Too high values: Missing data (locus dropout)
	Non-optimized values of clustering threshold	Setting a clustering threshold allows reads to be assembled in the same locus or in different loci by matching together similar sequences based on a given number of mismatches (which are defined by researchers)	Too low values: Repetitive regions and paralogs erroneously assembled together<br><br>Too high values: Missing data (reads from the same DNA fragment are clustered as different loci)
	Non-optimized values of minimum taxon coverage	The minimum number of (ingroup) samples with data for a given locus to be retained in the final data set.	Too low values: High proportion of missing data<br><br>Too high values: Considerable loss of loci with phylogenetic signal
PHYLOGENOMIC ANALYSIS	Systematic errors (Supermatrix approach)	Systematic errors occur because the assumptions of the underlying model are violated, including (I) homogeneity of the nucleotide/amino acid composition among lineages, (II) homogeneity of the substitution rate among lineages and (III) homogeneity in the substitution rate within nucleotide positions over time	Spurious phylogenetic relationships because of long-branch attraction and accumulation of convergences
	High proportion of missing data	The proportion of missing data in an alignment depends on:<br>(i) variable recovery during library preparation due to technical issues,<br>(ii) mutations at enzyme-cutting sites (amount of divergence among individuals/taxa)<br>(iii) sample coverage threshold established<br>(iv) taxon coverage threshold<br>(v) bioinformatic errors in identifying homology (clustering threshold)	1) introducing parameter misestimations, 2) decreasing resolving power, and 3) reducing the detection of multiple substitutions, which will ultimately produce misleading estimates of topology and branch lengths

Table S2 Studied taxa and their corresponding population number, country, province, locality, herbarium code, collector's name and collection date.

TAXON	POPULATION NUMBER	COUNTRY	PROVINCE	LOCALITY	VOUCHER INFORMATION	LEG	DATE
Cistus ladanifer L.	E016	SPAIN	Sevilla	Aznalcazar	SEV286741	S Martin-Hernanz, E Rubio	09/10/2016
Fumana thymifolia Spach	E013	SPAIN	Cádiz	Grazalema, Benamahoma	s/n	A Aparicio	01/07/2010
Halimium lasianthum (Lam.) Spach	E027	SPAIN	Cádiz	Alcalá de los Gazules, Pileta de la Reina	SEV286750	S Martin-Hernanz	06/11/2016
Helianthemum abelardoi Alcaraz	38	SPAIN	Alicante	Orihuela, Campoamor	SEV286547	A Aparicio, RG Albaladejo, F García & MA Carrasco	24/03/2011
Helianthemum aegyptiacum Mill.	89	SPAIN	Sevilla	La Puebla del Río, Pinar de Matatontos	SEV287169	A Aparicio, RG Albaladejo & C Parejo	24/03/2015
Helianthemum aganae Marrero Rodr. & R. Mesa	435	SPAIN	La Gomera	Not available	s/n	R Mesa Coello	05/09/2011
Helianthemum almeriense Pau	40	SPAIN	Murcia	Águilas, close to Cabo Cope	SEV286550	A Aparicio, RG Albaladejo, F García & MA Carrasco	24/03/2011
Helianthemum alypoides Losa & Rivas Goday	46	SPAIN	Almería	Sorbas, Río Aguas	SEV286554	A Aparicio, RG Albaladejo, F García & MA Carrasco	25/03/2011
Helianthemum angustatum Pomel	66	SPAIN	Granada	Baza, Sierra de Baza, Cortijo del Bordón	SEV286569	A Aparicio & RG Albaladejo	20/05/2011
Helianthemum antitauricum P.H.Davis & Coode	271	TURKEY	Adana	Arslantaş-Ayvat villages	s/n	Burcu Yesilyurt	13/07/2012
Helianthemum apenninum Mill. subsp. ***apenninum***	239	SPAIN	Granada	Escúzar	SEV287171	A Aparicio, RG Albaladejo, C de la Vega & E Rubio	21/04/2015
Helianthemum apenninum Mill. subsp. ***cantabricum*** (Laínz) G. López	368	SPAIN	León	Vega de Viejos, Cacabillo	SEV287170	A Aparicio, RG Albaladejo, S Martín-Hernanz & E Rubio	12/06/2017
Helianthemum apenninum Mill. subsp.	64	SPAIN	Almería	María, Sierra de María, La Peguera	SEV287172	A Aparicio & RG Albaladejo	19/05/2011

cavanillesianum (M.Laínz) G.López							
Helianthemum apenninum Mill. subsp. ***estevei*** (Peinado & Mart.Parras) G.López	75-2	SPAIN	Granada	Dílar, Trevenque	SEV286573	A Aparicio & RG Albaladejo	26/05/2011
Helianthemum apenninum Mill. subsp. ***stoechadifolium*** (Brot.) Samp.	26	SPAIN	Guadalajara	Albendiego, Sierra de Pela	SEV287173	J Arroyo & R Pérez-Barrales	14/06/2010
Helianthemum apenninum Mill. subsp. ***stoechadifolium*** (Brot.) Samp.	236	SPAIN	Huelva	Almonte, Matalascañas	SEV286539	A Aparicio	30/04/2015
Helianthemum apenninum Mill. subsp. ***suffruticosum*** (Boiss.) G. López	12	SPAIN	Málaga	Tolox, Sierra de las Nieves	SEV287174	A Aparicio & RG Albaladejo	26/07/2010
Helianthemum apenninum Mill. subsp. ***urrielense*** (M.Laínz) G.López	104	SPAIN	Cantabria	Camaleño, Picos de Europa, Fuente De	SEV286523	RG Albaladejo & C de Vega	12/04/2013
Helianthemum asperum Lag. ex Dunal	65	SPAIN	Almería	María, Sierra de María, La Piza	SEV287176	A Aparicio & RG Albaladejo	19/05/2011
Helianthemum bramwelliorum Marrero Rodr.	132	SPAIN	Lanzarote	Haría, Riscos de Guinate, Fuente de las Ovejas	Not available	Not available	03/06/1992
Helianthemum bramwelliorum Marrero Rodr.	412	SPAIN	Lanzarote	Haría, Riscos de Guinate, Fuente de las Ovejas	s/n	S Martín-Hernanz, M Marín Rodulfo, M Olangua Corral & A Reyes	14/05/2018
Helianthemum broussonetii Dunal ex DC.	397	SPAIN	La Palma	Barlovento, road to Garafía. Barranco Gallegos	SEV287630	RG Albaladejo, S Martín-Hernanz & M Olangua Corral	07/05/2018
Helianthemum broussonetii Dunal ex DC.	407	SPAIN	Tenerife	Afur, Roque Páez	SEV287631	RG Albaladejo, S Martín-Hernanz, M Olangua Corral & A Santos	11/05/2018
Helianthemum	224	SPAIN	Gran Canaria	San Nicolás de Tolentino	VIAL7730	Not available	Not

bystropogophyllum Svent.							available
Helianthemum bystropogophyllum Svent.	419	SPAIN	Gran Canaria	Casa forestal Pajonales	s/n	N Cabrera, I Guillermes, S Martín-Hernanz, M Marín Rodulfo, Jose Naranjo & M Olangua Corral	17/05/2018
Helianthemum canariense Pers.	135	SPAIN	Fuerteventura	Pájara, La Pared	MA768079	Alvarez, Calvo & Rios	28/02/2008
Helianthemum canariense Pers.	110	MOROCCO	El-Aïoum	May Taieb to el embalse Mohamed V	SEV286528	A Aparicio, J Aparicio, S Martin-Hernanz, E Rubio	11/04/2013
Helianthemum canariense Pers.	402	SPAIN	Tenerife	Los Roques	SEV287632	RG Albaladejo, S Martín-Hernanz, M Olangua Corral & A Santos	09/05/2018
Helianthemum canariense Pers.	414	SPAIN	Gran Canaria	Punta de Arinaga	SEV287633	S Martín-Hernanz & M Marín Rodulfo	16/05/2018
Helianthemum caput-felis Boiss.	275-1 R1	SPAIN	Alicante	Orihuela, Punta de Glea	SEV287178	A Aparicio, S Martin-Hernanz, E Rubio	14/03/2016
Helianthemum caput-felis Boiss.	275-1 R2	SPAIN	Alicante	Orihuela, Punta de Glea	SEV287178	A Aparicio, S Martin-Hernanz, E Rubio	14/03/2016
Helianthemum caput-felis Boiss.	121-1	MOROCCO	Nador	Farkhana, to the coast	SEV287179	A Aparicio, J Arroyo, RG Albaladejo & C Parejo	13/04/2013
Helianthemum ciliatum Pers.	150	TUNISIA	Gabés	Metlaoui, gorjes de Seldja	MA798199	Not available	25/03/2009
Helianthemum cinereum Pers. subsp. ***cinereum***	60	SPAIN	Murcia	Jumilla to Albatera	SEV286562	A Aparicio & RG Albaladejo	18/05/2016
Helianthemum cinereum Pers. subsp. ***guadiccianum*** (Font Quer & Rothm.) G.López	64	SPAIN	Almería	María, Sierra de María, La Peguera	SEV286568	A Aparicio & RG Albaladejo	19/05/2011
Helianthemum cinereum Pers. subsp. ***hieronymi*** (Sennen) G.López	62	SPAIN	Murcia	Alhama de Murcia, Sierra Espuña	SEV286566	A Aparicio & RG Albaladejo	19/05/2011
Helianthemum cinereum Pers.subsp. ***rotundifolium***	72	SPAIN	Jaen	Huelma, Sierra de Mágina, collado de la	SEV287180	A Aparicio & RG Albaladejo	26/05/2011

(Dunal) Greuter & Burdet				Cruz			
Helianthemum cirae A. Santos	400	SPAIN	La Palma	Caldera de Taburiente National Park, Roque de la Cumbrecita	s/n	RG Albaladejo, S Martín-Hernanz, M Olangua Corral & A Palomares	08/05/2018
Helianthemum cirae A. Santos	432	SPAIN	La Palma	Caldera de Taburiente National Park, Andén de la Cañada	s/n	Not available	15/06/2018
Helianthemum confertum Dunal	281	MOROCCO	Agadir	Amerskroud to Talaint	SEV287181	A Aparicio, J Aparicio, S Martin-Hernanz, E Rubio	29/03/2016
Helianthemum ellipticum Pers.	424	MOROCCO	Gareb	Mechra Hommadi to Hassi Berkane	SEV287634	A Aparicio, S Martín-Hernanz & E Rubio	04/06/2018
Helianthemum germanicopolitanum Bornm.	272	TURKEY	Çankiri	Kalecik-Çankırı road, parting of the İnandık ways	s/n	Burcu Yesilyurt	16/06/2011
Helianthemum getulum Pomel	374	MOROCCO	Agadir	Souss-Massa, Draâ: Afella Ighir, between Ait Mansour and Afella Ighir	MA913139	I Aizpuru, S Andrés-Sánchez, D Gutiérrez-Larruscaín, C Molina, J Pedrol, A Prunell, E Rico, A Rondríguez, C Urones	25/03/2015
Helianthemum gonzalezferreri Marrero Rodr.	222-2	SPAIN	Lanzarote	Haría, Macizo de Famara, El Bosquecillo	Not available	Not available	25/05/2010
Helianthemum gonzalezferreri Marrero Rodr.	411	SPAIN	Lanzarote	Haría, Macizo de Famara, El Bosquecillo	s/n	M Díaz-Bertrana, S Martín-Hernanz, M Marín Rodulfo, M Olangua Corral & A Reyes	14/05/2018
Helianthemum gorgoneum Webb	348	CAPE VERDE	Ihla Do Fogo	Chã das Caldeiras	SEV286753	I Hernanz, R Martin, S Martin-Hernanz	22/10/2016
Helianthemum grosii Pau & Font Quer	118	MOROCCO	Al-Hoceima	Circa Izemmourèn	SEV286534	A Aparicio, J Arroyo, RG Albaladejo & C Parejo	13/04/2013
Helianthemum grosii Pau & Font Quer	425	MOROCCO	Al-Hoceima	Rouadi, Dchar Maya, itineraire Tikkit	SEV287635	A Aparicio, S Martín-Hernanz & E Rubio	05/06/2018
Helianthemum guerrae Sánchez-Gómez, J.S.Carrion & M.A.Carrión	61	SPAIN	Murcia	Yecla, Sierra del Serral, La Boquera	SEV286564	A Aparicio & RG Albaladejo	19/05/2011

Helianthemum helianthemoides (Desf.) Sennen & Mauricio	426	MOROCCO	Immouzer Du Kandar	to Ifrane	SEV287636	A Aparicio, S Martín-Hernanz & E Rubio	05/06/2018
Helianthemum helianthemoides (Desf.) Sennen & Mauricio	429	MOROCCO	Ifrane	to Ras el Ma	SEV287637	A Aparicio, S Martín-Hernanz & E Rubio	05/06/2018
Helianthemum hirtum Mill.	55	SPAIN	Cuenca	Huete	SEV287182	A Aparicio & RG Albaladejo	18/05/2011
Helianthemum inaguae Marrero Rodr., González Martín & González Artiles	225	SPAIN	Gran Canaria	Inagua	VIAL 13308	Not available	Not available
Helianthemum inaguae Marrero Rodr., González Martín & González Artiles	418	SPAIN	Gran Canaria	Inagua, Andén de Tasarte	s/n	N Cabrera, I Guillermes, S Martín-Hernanz, M Marín Rodulfo, Jose Naranjo & M Olangua Corral	17/05/2018
Helianthemum juliae Wildpret	404	SPAIN	Tenerife	Cañadas del Teide National Park, Risco Verde	s/n	RG Albaladejo, S Martín-Hernanz, M Olangua Corral & A Santos	10/05/2018
Helianthemum juliae Wildpret	434	SPAIN	Tenerife	Cañadas del Teide National Park, Mesa del Obispo	s/n	RG Albaladejo, S Martín-Hernanz, M Olangua Corral, A Reyes, M Suárez & A Santos	17/07/2018
Helianthemum kahiricum Delile	113	MOROCCO	Guercif	15 km to Taza	SEV286539	A Aparicio, J Aparicio, S Martin-Hernanz, E Rubio	11/04/2016
Helianthemum kostchianum Boiss.	268	TURKEY	Konya	Aladağ road, close to Bademli village	s/n	Burcu Yesilyurt	12/05/2012
Helianthemum ledifolium (L.) Mill.	107	MOROCCO	Gouenfuda	to Jerada	SEV287183	A Aparicio, J Arroyo, RG Albaladejo & C Parejo	10/04/2013
Helianthemum linii A.Santos	399	SPAIN	La Palma	Tijarafe, Torre del Time	SEV287638	RG Albaladejo, S Martín-Hernanz & M Olangua Corral	07/05/2018
Helianthemum lippii (L.) Dum. Cours.	284	MOROCCO	Taroudant	Ville Noughaylle to Aguerd El Hed	SEV287184	A Aparicio, J Aparicio, S Martin-Hernanz, E Rubio	30/03/2016
Helianthemum lunulatum D.C.	185-0	FRANCE	Tende	Cultivated in Alpine Station Josphe Fourier from seeds collected in	s/n	R Douzet	08/09/2013

Col du Tende (1900 m)

Helianthemum lunulatum D.C.	329	ITALY	Ormea	Colle Caprauna, to Monte Armetta	SEV286755	A Aparicio, S Martin-Hernanz, E Rubio	01/07/2016
Helianthemum marifolium Mill. ***coquense*** Borja & Rivas Goday ex G. López	55	SPAIN	Cuenca	Huete	SEV286558	A Aparicio & RG Albaladejo	18/05/2011
Helianthemum marifolium Mill. subsp. ***andalusicum*** (Font Quer & Rothm.) G. López	266	SPAIN	Málaga	Alfarnate, Sierra de Enmedio	s/n	Not available	10/06/2015
Helianthemum marifolium Mill. subsp. ***frigidulum*** (Cuatrecasas) G. López	267	SPAIN	Jaén	Bélmez de la Moraleda, Sierra de Mágina, Carboneras	s/n	Not available	22/06/2015
Helianthemum marifolium Mill. subsp. ***marifolium***	85	SPAIN	Málaga	Antequera to Valle de Abdalajís	SEV287185	A Aparicio & RG Albaladejo	09/06/2011
Helianthemum marifolium Mill. subsp. ***molle*** (Cav.) G. López	251	SPAIN	Castellón	Alcalà de Xivert, Les Coves de Vinromà	SEV287186	A Aparicio, RG Albaladejo, C de la Vega & E Rubio	29/04/2015
Helianthemum marifolium Mill. subsp. ***origanifolium*** (Lam.) G. López	234	PORTUGA L	Sagres	Jaral close to La Fortaleza	SEV287187	A Aparicio	15/03/2015
Helianthemum marminorenseAlcaraz, Peinado & Mart. Parras	276	SPAIN	Murcia	San Pedro del Pinatar, duna de San Pedro	SEV286758	A Aparicio, S Martin-Hernanz, E Rubio	14/03/2016
Helianthemum morisianum Bertol.	395	ITALY	Sardinia	Oristano, Domos de Pirastera	SEV287639	A Aparicio	21/03/2018
Helianthemum motae Sánchez-Gómez, J.F. Jiménez & J.B.Vera	277	SPAIN	Murcia	Águilas, playa de los Cocedores	SEV286759	A Aparicio, S Martin-Hernanz, E Rubio	14/03/2016
Helianthemum neopiliferum Muñoz Garm. & Navarro	125	SPAIN	Granada	Arenas del Rey, La Resinera	SEV286536	J Arroyo, R Molina & R Santos	26/05/2013

Helianthemum nummularium Mill. subsp. ***nummularium***	300	ITALY	Sicily	Cesaro towards San Fratello, Nebrodi	SEV287189	A Aparicio, S Martin-Hernanz, E Rubio	27/04/2016
Helianthemum nummularium Mill. subsp. ***grandiflorum*** (Scop.) Schinz & Thell.	332	FRANCE	Bédain	Mont Ventoux	SEV287188	A Aparicio, S Martin-Hernanz, E Rubio	02/07/2016
Helianthemum nummularium Mill. subsp. ***lycaonicum*** Coode & Cullen	269	TURKEY	Isparta	Yenice village	s/n	Burcu Yesilyurt	01/07/2012
Helianthemum nummularium Mill. subsp. ***obscurum*** Holub	322	ITALY	Panice Soprana	Col de Tende	SEV287190	A Aparicio, S Martin-Hernanz, E Rubio	29/06/2016
Helianthemum nummularium subsp. ***semiglabrum*** (Badarò) M. Proctor	327	ITALY	Colle Di Nava	to Ponte di Nava	SEV286760	A Aparicio, S Martin-Hernanz, E Rubio	30/06/2016
Helianthemum obtusifolium Dunal	294	CYPRUS	Perivolia	pr. Cape Kiti	SEV287191	A Aparicio, MA Carrasco, S Martin-Hernanz	13/04/2016
Helianthemum oelandicum (L.) DC. subsp. ***italicum*** (L.) Font Quer & Rothm.	325	ITALY	Valdieri	Rocca San Giovanni-Saben	SEV287193	A Aparicio, S Martin-Hernanz, E Rubio	30/06/2016
Helianthemum oelandicum (L.) DC. subsp. ***alpestris*** (Jacq.) Breistr.	184	FRANCE	Cervières	Col d'Izouard	SEV286538	R Douzet	03/07/2013
Helianthemum oelandicum (L.) DC. subsp. ***incanum*** (Willk.) G. López	72	SPAIN	Jaén	Huelma, Sierra de Mágina, collado de la Cruz	SEV287192	A Aparicio & RG Albaladejo	26/05/2011
Helianthemum oelandicum subsp. ***pourretii*** (Timb.-Lagr.) Greuter & Burdet	332	FRANCE	Bédain	Mont Ventoux	SEV286761	A Aparicio, S Martin-Hernanz, E Rubio	02/07/2016
Helianthemum ordosicum Zhao, Zhu & Cao	127	CHINA	Inner Mongolia	Ordos, Quianlishan	s/n	Zhihao Su	05/05/2010

Helianthemum pannosum Boiss.	75-5	SPAIN	Granada	Dílar, Trevenque	SEV286574	A Aparicio & RG Albaladejo	26/05/2011
Helianthemum pannosum Boiss.	75-6	SPAIN	Granada	Dílar, Trevenque	SEV286575	A Aparicio & RG Albaladejo	26/05/2011
Helianthemum papillare Boiss.	106	MOROCCO	Gouenfuda	to Jerada	SEV286525	A Aparicio, J Arroyo, RG Albaladejo & C Parejo	10/04/2013
Helianthemum pergamaceum Pomel	115	MOROCCO	Aknoul	to Kassita	SEV287194	A Aparicio, J Arroyo, RG Albaladejo & C Parejo	12/04/2013
Helianthemum polyanthum Pers.	117	MOROCCO	Al-Hoceima	6 km to Izemmourèn	SEV286532	A Aparicio, J Arroyo, RG Albaladejo & C Parejo	12/04/2013
Helianthemum polyanthum Pers.	109	MOROCCO	Berkane	Taforalt	SEV287195	A Aparicio, J Arroyo, RG Albaladejo & C Parejo	11/04/2013
Helianthemum polygonoides Peinado, Mart. Parras, Alcaraz & Espuelas	59 R1	SPAIN	Albacete	Tobarra, Saladar de Cordovilla	SEV286561	A Aparicio & RG Albaladejo	19/05/2011
Helianthemum polygonoides Peinado, Mart. Parras, Alcaraz & Espuelas	59 R2	SPAIN	Albacete	Tobarra, Saladar de Cordovilla	SEV286561	A Aparicio & RG Albaladejo	19/05/2011
Helianthemum pomeridianum Dunal	352	MOROCCO	Taroudant	Sidi Abdellah Oussaid-Alegjane	SEV286762	A. Aparicio, FJ Aparicio et al.	13/11/2016
Helianthemum pomeridianum Dunal	144-0	MOROCCO	Marrackech	Taroudant	MA472377	Not available	12/07/1989
Helianthemum raskebdanae M.A.Alonso, M.B.Crespo, Juan & L.Sáez	274	MOROCCO	Nador	Ras-el-Ma, 2 km to Saidia road	SEV286763	A Aparicio, J Aparicio, S Martin-Hernanz, E Rubio	01/04/2016
Helianthemum ruficomum Spreng.	111	MOROCCO	El-Aïoum	May Taieb, to reservoir Mohamed V	SEV287196	A Aparicio, J Arroyo, RG Albaladejo & C Parejo	11/04/2013
Helianthemum salicifolium (L.) Mill.	76	SPAIN	Granada	La Zubia, Cumbres Verdes	SEV287197	A Aparicio & RG Albaladejo	26/05/2011
Helianthemum sancti-antonii Schweinf. ex Asch.	389	ISRAEL	Mitzpe Ramon	Gewanim	SEV287640	A Aparicio, RG Albaladejo & S Martín-Hernanz	03/03/2018

& Schweinf.							
Helianthemum sanguineum (Lag.) Lag. ex Dunal	233	SPAIN	Málaga	Ardales, Sierra del Almorchón, between Pico del Convento and Alto del Almorchón	SEV287198	A Aparicio, RG Albaladejo, C de la Vega & E Rubio	13/03/2015
Helianthemum sanguineum (Lag.) Lag. ex Dunal	295	CYPRUS	Ayia Eirini	Ayia Eirni	SEV286764	A Aparicio, MA Carrasco, S Martin-Hernanz	14/04/2016
Helianthemum sauvagei Raynaud.	282	MOROCCO	Agadir	Amerskroud towards Talaint	SEV286765	A Aparicio, J Aparicio, S Martin-Hernanz, E Rubio	29/03/2016
Helianthemum scopulicolum L. Sáez, Alomar & Rosselló	363	SPAIN	Baleares	Serra de Tramuntana, Cap Fabioler, Mallorca	SEV287199	M Vicens	26/05/2017
Helianthemum sessiliflorum Pers.	384	ISRAEL	Nir Yitzhak	along roadsides	SEV287641	A Aparicio, RG Albaladejo, O Fragman-Sapir & S Martín-Hernanz	02/03/2018
Helianthemum sicanorum Brullo, Giusso & Sciandr.	297	ITALY	Sicily	Gela Torre Manfria	SEV286766	A Aparicio, S Martin-Hernanz, E Rubio	26/04/2016
Helianthemum songaricum Zhao, Zhu & Cao	175	KAZA-KHSTAN	Almaty	Charyn National Park. Top of main gorge	E00282039	Rae, David Alasdair H	20/05/2008
Helianthemum sp nov 1*	405	SPAIN	Tenerife	Chirche, Barranco Bermejo	s/n	RG Albaladejo, S Martín-Hernanz, M Olangua Corral & A Santos	10/05/2018
Helianthemum sp nov 2*	398	SPAIN	La Palma	Puntagorda. Between Barranco de San Marco and Barranco del Roque	s/n	RG Albaladejo, S Martín-Hernanz & M Olangua Corral	07/05/2018
Helianthemum sp nov 2*	432	SPAIN	La Palma	Caldera de Taburiente National Park, Andén de la Cañada	s/n	No availableNot available	15/06/2018
Helianthemum sp nov 3*	437	SPAIN	Fuerteventura	Cultivated plants	s/n	Marco Díaz-Bertrana	jun-18

Helianthemum squamatum Pers.	57	SPAIN	Albacete	Tobarra to Cordovilla	SEV286559	A Aparicio & RG Albaladejo	18/05/2011
Helianthemum squamatum Pers.	46	SPAIN	Almería	Sorbas, Rio Aguas	SEV286555	A Aparicio, RG Albaladejo, F García & MA Carrasco	25/03/2011
Helianthemum stipulatum C. Chr.	290	CYPRUS	Perivolia	Cape Kiti, Tower of Rigenas	SEV287200	A Aparicio, MA Carrasco, S Martin-Hernanz	12/04/2016
Helianthemum syriacum (Jacq.) Dum.-Cours.	278 R1	SPAIN	Almería	Turre to Rambla del Estrecho	SEV287201	A Aparicio, S Martin-Hernanz, E Rubio	15/03/2016
Helianthemum syriacum (Jacq.) Dum.-Cours.	278 R2	SPAIN	Almería	Turre to Rambla del Estrecho	SEV287201	A Aparicio, S Martin-Hernanz, E Rubio	15/03/2016
Helianthemum syriacum (Jacq.) Dum.-Cours.	288	CYPRUS	Larnaca	Xylotimou, Dhekelia	SEV287202	A Aparicio, MA Carrasco, S Martin-Hernanz	12/04/2016
Helianthemum teneriffae Coss.	401	SPAIN	Tenerife	Ladera de Güímar, Canal de los Mil	s/n	RG Albaladejo, S Martín-Hernanz, M Olangua Corral & A Santos	09/05/2018
Helianthemum tholiforme J. Ortega & B.Navarro	420	SPAIN	Gran Canaria	Montaña del Tauro	s/n	S Martín-Hernanz, M Marín Rodulfo & M Olangua Corral	18/05/2018
Helianthemum thymiphyllum Svent.	375	SPAIN	Fuerteventura	Riscos de Jandia	Not available	Not available	18/07/2017
Helianthemum thymiphyllum Svent.	410	SPAIN	Lanzarote	Road between Mojón and Guatiza	SEV287642	S Martín-Hernanz, M Marín Rodulfo & M Olangua Corral	13/05/2018
Helianthemum tinetense M.Mayor & Fern.Benito	369	SPAIN	Oviedo	Tineo, Rodical hacia la Castañera	SEV287203	A Aparicio, RG Albaladejo, S Martín-Hernanz & E Rubio	13/06/2017
Helianthemum ventosum Boiss.	387	ISRAEL	Tlalim-Mitzpe Ramon	Hill of limestone rock	SEV287643	A Aparicio, RG Albaladejo, O Fragman-Sapir & S Martín-Hernanz	02/03/2018
Helianthemum vesicarium Boiss.	388	ISRAEL	Mitzpe Ramon	Wadi Nizzana	SEV287644	A Aparicio, RG Albaladejo & S Martín-Hernanz	03/03/2018
Helianthemum violaceum Pers.	317	SPAIN	Alicante	Biar, Sierra de Biar	SEV287204	A Aparicio, S Martin-Hernanz, E Rubio	18/05/2016
Helianthemum virgatum (Desf.) Pers.	105	SPAIN	Berkane	to Taforalt	SEV286524	A Aparicio, J Arroyo, RG Albaladejo & C Parejo	10/04/2013

Helianthemum viscarium Boiss. & Reut.	62	SPAIN	Murcia	Alhama de Murcia, Sierra Espuña	SEV286565	A Aparicio & RG Albaladejo	19/05/2011
Helianthemum viscidulum Boiss. subsp. ***raynaudii*** (Ortega Oliv., Romero García & C.Morales) G. López	70	SPAIN	Granada	Quéntar, Puerto de la Mora, road to El Pozuelo	SEV286570	A Aparicio & RG Albaladejo	20/05/2011
Helianthemum viscidulum Boiss. subsp. ***viscidulum***	319	SPAIN	Granada	Alhama de Granada, road to Venta Rodríguez	SEV287205	A Aparicio, S Martin-Hernanz, E Rubio	19/05/2016
Tuberaria macrosepala (Coss.) Willk.	E008	SPAIN	Sevilla	Dos Hermanas, La Corchuela	SEV286769	A Aparicio, RG Albaladejo	04/04/2011

* Non-described species (Santos-Guerra, 2014; Díaz-Bertrana com. pers)

Table S3 Number of taxa (and species) per section included in this study.

SECTIONS	TOTAL TAXA (SPECIES)	TAXA (SPECIES) HERE INCLUDED	TAXA (SPECIES) PERCENTAGE
Argyrolepis	1 (1)	1 (1)	100% (100%)
Atlanthemum	1 (1)	1 (1)	100% (100%)
Brachypetalum	5 (5)	4 (4)	80% (80%)
Caput-felis	1 (1)	1 (1)	100% (100%)
Eriocarpum s.l. (incl. *Pseudomacularia*)	28 (28)	17 (17)	60.71% (60.71%)
Helianthemum	63 (47)	52 (39)	82.54% (82.98%)
Lavandulaceum	2 (2)	2 (2)	100% (100%)
Macularia	2 (2)	2 (2)	100% (100%)
Pseudocistus	37 (17)	18 (6)	48.65% (35.29%)
TOTAL	140 (104)	98 (73)	70% (70.19%)

Notes: In bold, sections of subg. *Plectolobum*; otherwise, all sections belong to subg. *Helianthemum*. Floristic publications reviewed to establish the number of taxa and species per section are listed in the Appendix S1 from Aparicio et al. (2017).

Table S4 Sequence Read Archive (SRA) accession code and number of reads and loci per sample recovered in the sequencing and bioinformatics process. The whole sequence dataset can be found in BioProject (accession number: PRJNA573639).

Taxon	Population number	SRA accession code	Including in exploratory (Subset) and/or final (Full set) PyRAD Assembly	Million read-pairs (or reads)	Assembled million reads in PEAR (merged)	Not assembled million reads in PEAR (unmerged)	Discarded reads in PEAR	Number of loci with >N depth and passed paralog filter (step 3 PyRAD) under MaxResol (minCov15%)	Number of loci recovered in final data set for each taxon (step 6 PyRAD) under MaxResol (minCov15%)	Number of loci with >N depth and passed paralog filter (step 3 PyRAD) under MinError (minCov15%)	Number of loci recovered in final data set for each taxon (step 6 PyRAD) under MinError (minCov15%)
Cistus ladanifer L.	E016	SAMN12818844	Full set	6.737	4.450 (66.058%)	2.286 (33.942%)	30 (0.000%)	57961	2256	24393	686
Fumana thymifolia Spach	E013	SAMN12818845	Subset / Full set	4.91	3.289 (66.967%)	1.622 (33.029%)	185 (0.004%)	93630	1907	30779	564
Halimium lasianthum (Lam.) Spach	E027	SAMN12818846	Full set	5.515	3.758 (68.141%)	1.757 (31.858%)	47 (0.001%)	223744	1813	18869	517
Helianthemum abelardoi Alcaraz	38	SAMN12818847	Subset / Full set	6.617	4,943 (74.700%)	1.677 (25.294%)	384 (0.006%)	456161	13352	23656	2511
Helianthemum aegyptiacum Mill.	89	SAMN12818848	Subset / Full set	3.345	2.568 (76.779%)	0.776 (23.216%)	160 (0.005%)	152366	10965	12960	1882
Helianthemum aganae Marrero Rodr. & R. Mesa	435	SAMN12818849	Full set	5.644	2.888 (51.161%)	2.757 (48.838%)	72 (0.001%)	39469	10447	20053	3462
Helianthemum almeriense Pau	40	SAMN12818850	Subset / Full set	2.407	1.590 (66.091%)	0.816 (33.909%)	19 (0.001%)	52316	11335	11263	1857
Helianthemum alypoides Losa & Rivas Goday	46	SAMN12818851	Subset / Full set	1.622	1.177 (72.538%)	0.445 (27.461%)	20 (0.001%)	50584	10791	8058	1380

Helianthemum angustatum Pomel	66	SAMN12818852	Subset / Full set	3.435	2.341 (68.170%)	1.093 (31.826%)	120 (0.003%)	141195	6747	15015	1617
Helianthemum antitauricum P.H.Davis & Coode	271	SAMN12818853	Subset / Full set	0.934	724 (77.540%)	0.209 (22.458%)	16 (0.002%)	35210	5718	7632	1985
Helianthemum apenninum Mill. subsp. ***apenninum***	239	SAMN12818854	Subset / Full set	1.614	1.299 (80.491%)	0.314 (19.504%)	82 (0.005%)	83749	9528	3896	594
Helianthemum apenninum Mill. subsp. ***cantabricum*** (Laínz) G. López	368	SAMN12818855	Full set	4.247	2.755 (64.861%)	1.492 (35.137%)	67 (0.002%)	115251	12254	15162	2317
Helianthemum apenninum Mill. subsp. ***cavanillesianum*** (M.Laínz) G.López	64	SAMN12818856	Subset / Full set	2.967	2.101 (70.824%)	0.865 (29.174%)	65 (0.002%)	102437	12256	11153	1868
Helianthemum apenninum Mill. subsp. ***estevei*** (Peinado & Mart.Parras) G.López	75-2	SAMN12818857	Full set	2.528	1.825 (72.178%)	0.702 (27.765%)	14 (0.057%)	137924	6912	4362	625
Helianthemum apenninum Mill. subsp. ***stoechadifolium*** (Brot.) Samp.	26	SAMN12818858	Subset	4.106	3.268 (79.594%)	0.837 (20.399%)	267 (0.007%)	293954	–	6676	884
Helianthemum apenninum Mill. subsp. ***stoechadifolium*** (Brot.) Samp.	236	SAMN12818859	Full set	2.886	2.043 (70.761%)	0.844 (29.237%)	48 (0.002%)	59045	11332	15239	2252

Helianthemum apenninum Mill. subsp. ***suffruticosum*** (Boiss.) G. López	12	SAMN12818860	Subset / Full set	2.222	1.628 (73.268%)	0.594 (26.729%)	72 (0.003%)	67216	9852	4763	745
Helianthemum apenninum Mill. subsp. ***urrielense*** (M.Laínz) G.López	104	SAMN12818861	Subset / Full set	6.693	4.367 (65.250%)	2.326 (34.748%)	86 (0.001%)	97326	12766	25303	3242
Helianthemum asperum Lag. ex Dunal	65	SAMN12818862	Subset / Full set	3.022	2.130 (70.459%)	0.893 (29.538%)	84 (0.003%)	153179	11383	11940	788
Helianthemum bramwelliorum Marrero Rodr.	132	SAMN12818863	Full set	1.015	0.881 (86.827%)	0.133 (13.113%)	604 (0.060%)	89977	5802	2255	481
Helianthemum bramwelliorum Marrero Rodr.	412	SAMN12818864	Full set	2.484	1.077 (43.338%)	1.408 (56.661%)	42 (0.002%)	31287	8667	8153	1783
Helianthemum broussonetii Dunal ex DC.	397	SAMN12818865	Full set	3.939	2.236 (56.770%)	1.702 (43.228%)	87 (0.002%)	36385	9466	10264	90045
Helianthemum broussonetii Dunal ex DC.	407	SAMN12818866	Full set	1.978	1.019 (51.527%)	0.959 (48.472%)	34 (0.002%)	36385	7471	4959	25398
Helianthemum bystropogophyllum Svent.	224	SAMN12818867	Full set	0.501	0.464 (92.425%)	0.038 (7.564%)	56 (0.011%)	29073	5814	2588	527
Helianthemum bystropogophyllum Svent.	419	SAMN12818868	Full set	3.585	1.957 (54.597%)	1.627 (45.401%)	60 (0.002%)	50513	9990	13444	2735
Helianthemum canariense Pers.	135	SAMN12818869	Subset	0.866	0.688 (79.444%)	0.178 (20.554%)	19 (0.002%)	38973	_	6326	2047

Helianthemum canariense Pers.	110	SAMN12818870	Subset / Full set	1.695	1.114 (65.730%)	0.581 (34.269%)	21 (0.001%)	50325	6849	11406	3363
Helianthemum canariense Pers.	402	SAMN12818871	Full set	2.318	1.138(49.115%)	1.179 (50.884%)	27 (0.001%)	36838	5984	13036	3682
Helianthemum canariense Pers.	414	SAMN12818872	Full set	2.568	1.302 (50.709%)	1.266 (49.290%)	42 (0.002%)	38397	6158	15468	3993
Helianthemum caput-felis Boiss.	275-1 R1	SAMN12818873	Subset / Full set	1.111	0.791 (71.216%)	0.319 (28.780%)	43 (0.004%)	49355	5766	6919	926
Helianthemum caput-felis Boiss.	275-1 R2	SAMN12818874	Subset / Full set	2.433	1.723 (70.806%)	0.710 (29.190%)	106 (0.004%)	94504	6699	21736	1780
Helianthemum caput-felis Boiss.	121-1	SAMN12818875	Subset / Full set	2.108	1.407 (66.771%)	0.700 (33.227%)	50 (0.002%)	66745	3532	14520	1703
Helianthemum ciliatum Pers.	150	SAMN12818876	Full set	2.18	1.827 (83.819%)	0.353 (16.177%)	80 (0.004%)	82659	11394	11067	1720
Helianthemum cinereum Pers. subsp. ***cinereum***	60	SAMN12818877	Subset / Full set	6.737	4.450 (66.058%)	2.286 (33.942%)	30 (0.000%)	553403	8158	31707	1302
Helianthemum cinereum Pers. subsp. ***guadiccianum*** (Font Quer & Rothm.) G.López	64	SAMN12818878	Subset / Full set	7.079	5.072 (71.652%)	2.007 (28.345%)	193 (0.003%)	483286	8694	46518	1850
Helianthemum cinereum Pers. subsp. ***hieronymi*** (Sennen) G.López	62	SAMN12818879	Subset / Full set	4.251	3.355 (78.915%)	0.896 (21.079%)	269 (0.006%)	293305	8372	20634	1480

Helianthemum cinereum Pers.subsp. ***rotundifolium*** (Dunal) Greuter & Burdet	72	SAMN12818880	Subset / Full set	8.033	6.219 (77.421%)	1.813 (22.573%)	459 (0.006%)	800433	7939	35640	1591
Helianthemum cirae A. Santos	400	SAMN12818881	Full set	2.251	0.972 (43.181%)	1.279 (56.818%)	28 (0.001%)	26982	8493	5905	1433
Helianthemum cirae A. Santos	432	SAMN12818882	Full set	1.486	0.617 (41.526%)	0.869 (58.473%)	12 (0.001%)	21677	6892	3911	937
Helianthemum confertum Dunal	281	SAMN12818883	Subset / Full set	5.43	3.503 (64.514%)	1.927 (35.484%)	72 (0.001%)	105991	3666	22574	4538
Helianthemum ellipticum Pers.	424	SAMN12818884	Full set	4.324	1.993 (46.075%)	2.332 (53.924%)	55 (0.001%)	46520	6466	20240	4251
Helianthemum germanicopolitanum Bornm.	272	SAMN12818885	Subset / Full set	6.876	5.241 (76.225%)	1.634 (23.767%)	524 (0.008%)	474204	6982	20302	1532
Helianthemum getulum Pomel	374	SAMN12818886	Full set	2.068	1.505 (72.770%)	0.562 (27.177%)	1.110 (0.054%)	67481	6967	13470	3529
Helianthemum gonzalezferreri Marrero Rodr.	222-2	SAMN12818887	Full set	1.49	1.291 (86.653%)	0.198 (13.284%)	928 (0.062%)	146824	6201	186	627
Helianthemum gonzalezferreri Marrero Rodr.	411	SAMN12818888	Full set	1.809	0.794 (43.903%)	1.015 (56.096%)	23 (0.001%)	82659	7464	4308	915
Helianthemum gorgoneum Webb	348	SAMN12818889	Full set	4.571	2.830 (61.920%)	1.741 (38.079%)	52 (0.001%)	52446	7628	31258	4499
Helianthemum grosii Pau & Font Quer	118	SAMN12818890	Subset / Full set	2.221	1.645 (74.019%)	0.577 (25.976%)	111 (0.005%)	130312	8414	3938	514
Helianthemum grosii Pau & Font Quer	425	SAMN12818891	Full set	3.806	2.210 (58.071%)	1.596 (41.925%)	162 (0.004%)	173327	8495	9179	1166

Helianthemum guerrae Sánchez-Gómez, J.S.Carrion & M.A.Carrión	61	SAMN12818892	Subset / Full set	2.144	1.357 (63.277%)	0.787 (36.721%)	29 (0.001%)	50387	10913	9892	1638
Helianthemum helianthemoides (Desf.) Sennen & Mauricio	426	SAMN12818893	Full set	3.568	1.627 (45.601%)	1.941 (54.397%)	63 (0.002%)	59016	8336	6814	1127
Helianthemum helianthemoides (Desf.) Sennen & Mauricio	429	SAMN12818894	Full set	6.92	3.807 (55.010%)	3.113 (44.987%)	233 (0.003%)	308173	8754	19126	949
Helianthemum hirtum Mill.	55	SAMN12818895	Subset / Full set	2.403	1.667 (69.368%)	0.736 (30.629%)	77 (0.003%)	96793	11060	5573	771
Helianthemum inaguae Marrero Rodr., González Martín & González Artiles	225	SAMN12818896	Full set	0.336	0.304 (90.453%)	0.032 (9.542%)	15 (0.004%)	20969	5028	574	186
Helianthemum inaguae Marrero Rodr., González Martín & González Artiles	418	SAMN12818897	Full set	0.935	0.364 (38.940%)	0.571 (61.060%)	6 (0.001%)	18953	5591	1616	410
Helianthemum juliae Wildpret	404	SAMN12818898	Full set	2.692	1.150 (42.691%)	1.543 (57.308%)	20 (0.001%)	30206	9346	8019	1756
Helianthemum juliae Wildpret	434	SAMN12818899	Full set	5.218	2.344 (44.912%)	2.875 (55.084%)	187 (0.004%)	35878	9984	19811	3358
Helianthemum kahiricum Delile	113	SAMN12818900	Subset / Full set	3.678	2.430 (66.077%)	1.247 (33.920%)	102 (0.003%)	94410	7507	18790	4218
Helianthemum kostchianum	268	SAMN12818901	Subset / Full set	0.809	0.598 (73.912%)	0.211 (26.087%)	11 (0.001%)	27533	7507	1111	206

Boiss.

Helianthemum ledifolium (L.) Mill.	107	SAMN12818902	Full set	6.516	4.624 (70.961%)	1.892 (29.036%)	152 (0.002%)	130147	7261	27345	2444
Helianthemum linii A.Santos	399	SAMN12818903	Full set	2.35	1.240 (52.788%)	1.109 (47.210%)	46 (0.002%)	34444	8434	7848	1796
Helianthemum lippii (L.) Dum. Cours.	284	SAMN12818904	Subset / Full set	2.528	1.823 (72.100%)	0.705 (27.895%)	109 (0.004%)	91694	7671	15389	3940
Helianthemum lunulatum D.C.	185-0	SAMN12818905	Subset / Full set	4.929	2.985 (60.560%)	1.944 (39.439%)	32 (0.001%)	57781	6562	24147	2136
Helianthemum lunulatum D.C.	329	SAMN12818906	Full set	7.079	5.072 (71.652%)	2.007 (28.345%)	193 (0.003%)	312693	6930	21643	1779
Helianthemum marifolium Mill. ***coquense*** Borja & Rivas Goday ex G. López	55	SAMN12818907	Subset / Full set	5.161	3.883 (75.230%)	1.278 (24.762%)	438 (0.008%)	350137	8638	16104	1921
Helianthemum marifolium Mill. subsp. ***andalusicum*** (Font Quer & Rothm.) G. López	266	SAMN12818908	Subset / Full set	3.154	2.169 (68.794%)	0.984 (31.193%)	420 (0.013%)	71033	7179	16001	1832
Helianthemum marifolium Mill. subsp. ***frigidulum*** (Cuatrecasas) G. López	267	SAMN12818909	Subset / Full set	3.976	2.790 (70.167%)	1.186 (29.824%)	378 (0.010%)	148219	7296	13660	1695
Helianthemum marifolium Mill. subsp.	85	SAMN12818910	Full set	3.506	2.522 (71.935%)	0.984 (28.059%)	210 (0.006%)	135253	8543	13009	1750

marifolium											
Helianthemum marifolium Mill. subsp. ***molle*** (Cav.) G. López	251	SAMN12818911	Subset / Full set	4.958	3.640 (73.420%)	1.318 (26.574%)	304 (0.006%)	265704	8742	24978	2200
Helianthemum marifolium Mill. subsp. ***origanifolium*** (Lam.) G. López	234	SAMN12818912	Subset / Full set	2.841	2.323 (81.758%)	0.518 (18.237%)	156 (0.005%)	249339	7861	9982	1168
Helianthemum marminorense Alcaraz, Peinado & Mart. Parras	276	SAMN12818913	Subset / Full set	2.037	1.449 (71.140%)	0.588 (28.858%)	45 (0.002%)	76406	10162	6496	980
Helianthemum morisianum Bertol.	395	SAMN12818914	Full set	4.441	2.263 (50.950%)	2.178 (49.048%)	84 (0.002%)	88249	8604	7525	1216
Helianthemum motae Sánchez-Gómez, J.F. Jiménez & J.B.Vera	277	SAMN12818915	Subset / Full set	1.173	0.840 (71.618%)	0.333 (28.381%)	13 (0.001%)	36577	3376	4695	606
Helianthemum neopiliferum Muñoz Garm. & Navarro	125	SAMN12818916	Full set	6.644	4.857 (73.107%)	1.787 (26.891%)	141 (0.002%)	188157	12868	24871	3237
Helianthemum nummularium Mill. subsp. ***nummularium***	300	SAMN12818917	Subset / Full set	1.728	1.057 (61.173%)	0.671 (38.825%)	26 (0.002%)	42145	7708	3423	502
Helianthemum nummularium Mill. subsp. ***grandiflorum*** (Scop.) Schinz & Thell.	332	SAMN12818918	Subset / Full set	4.353	2.722 (62.535%)	1.631 (37.463%)	84 (0.002%)	80035	12141	19118	2726

Helianthemum nummularium Mill. subsp. ***lycaonicum*** Coode & Cullen	269	SAMN12818919	Subset / Full set	0.759	0.633 (83.296%)	0.127 (16.701%)	17 (0.002%)	40726	6846	1964	323
Helianthemum nummularium Mill. subsp. ***obscurum*** Holub	322	SAMN12818920	Subset / Full set	5.289	2,972,589 / 5,289,266 (56.200%)	2,316,622 / 5,289,266 (43.799%)	55 / 5,289,266 (0.001%)	71011	12419	20460	2658
Helianthemum nummularium subsp. ***semiglabrum*** (Badarò) M. Proctor	327	SAMN12818921	Subset / Full set	5.137	3.418 (66.536%)	1.719 (33.461%)	146 (0.003%)	230515	12605	14195	1902
Helianthemum obtusifolium Dunal	294	SAMN12818922	Subset / Full set	3.313	2.353 (71.009%)	0.960 (28.988%)	88 (0.003%)	155398	10113	8550	1017
Helianthemum oelandicum (L.) DC. subsp. ***italicum*** (L.) Font Quer & Rothm.	325	SAMN12818923	Subset / Full set	7.079	5.072 (71.652%)	2.007 (28.345%)	193 (0.003%)	726313	7989	17275	1571
Helianthemum oelandicum (L.) DC. subsp. ***alpestris*** (Jacq.) Breistr.	184	SAMN12818924	Full set	8.033	6.219 (77.421%)	1.813 (22.573%)	459 (0.006%)	498138	8898	24864	1285
Helianthemum oelandicum (L.) DC. subsp. ***incanum*** (Willk.) G. López	72	SAMN12818925	Subset / Full set	3.522	2.581 (73.281%)	0.941 (26.714%)	198 (0.006%)	168079	7673	20484	1706

Helianthemum oelandicum subsp. ***pourretii*** (Timb.-Lagr.) Greuter & Burdet	332	SAMN12818926	Subset / Full set	4.125	2.801 (67.902%)	1.324 (32.094%)	132 (0.003%)	172675	7535	9216	1229
Helianthemum ordosicum Zhao, Zhu & Cao	127	SAMN12818927	Full set	2.882	1.992 (69.125%)	0.888 (30.820%)	1.587 (0.055%)	60042	6693	15147	3044
Helianthemum pannosum Boiss.	75-5	SAMN12818928	Subset / Full set	7.079	5.072 (71.652%)	2.007 (28.345%)	193 (0.003%)	1070534	6969	34971	994
Helianthemum pannosum Boiss.	75-6	SAMN12818929	Full set	6.755	5.060 (74.916%)	1.694 (25.074%)	696 (0.010%)	449796	7052	31845	1427
Helianthemum papillare Boiss.	106	SAMN12818930	Subset / Full set	4.336	3.147 (72.603%)	1.187 (27.390%)	291 (0.007%)	131316	6579	14576	1665
Helianthemum pergamaceum Pomel	115	SAMN12818931	Subset / Full set	3.998	2.672 (66.843%)	1.326 (33.156%)	48 (0.001%)	62600	12080	15402	2372
Helianthemum polyanthum Pers.	117	SAMN12818932	Subset / Full set	3.077	2.160 (70.191%)	0.917 (29.782%)	860 (0.028%)	71208	7524	14077	1662
Helianthemum polyanthum Pers.	109	SAMN12818933	Subset / Full set	1.509	1.128 (74.739%)	0.381 (25.255%)	89 (0.006%)	67359	6611	6860	1068
Helianthemum polygonoides Peinado, Mart. Parras, Alcaraz & Espuelas	59 R1	SAMN12818934	Subset / Full set	3.855	2.607 (67.639%)	1.247 (32.360%)	44 (0.001%)	160177	10650	21976	1637
Helianthemum polygonoides Peinado, Mart. Parras, Alcaraz & Espuelas	59 R2	SAMN12818935	Subset / Full set	5.378	3.753 (69.781%)	1.625 (30.218%)	72 (0.001%)	106574	11855	27934	3278
Helianthemum	352	SAMN12818936	Full set	4.287	3.244	1.043	83 (0.002%)	74619	6166	23034	2063

pomeridianum Dunal					(75.676%)	(24.322%)					
Helianthemum pomeridianum Dunal	144-0	SAMN12818937	Subset / Full set	2.029	1.485 (73.209%)	0.543 (26.787%)	93 (0.005%)	69830	5872	11866	1340
Helianthemum raskebdanae M.A.Alonso, M.B.Crespo, Juan & L.Sáez	274	SAMN12818938	Subset / Full set	3.639	2.615 (71.873%)	1.023 (28.125%)	53 (0.001%)	72093	12597	21508	2993
Helianthemum ruficomum Spreng.	111	SAMN12818939	Full set	6.418	4.549 (70.884%)	1.868 (29.113%)	162 (0.003%)	238875	13000	41836	2592
Helianthemum salicifolium (L.) Mill.	76	SAMN12818940	Subset / Full set	3.293	2.289 (69.518%)	1.004 (30.479%)	86 (0.003%)	97060	6782	17194	1880
Helianthemum sancti-antonii Schweinf. ex Asch. & Schweinf.	389	SAMN12818941	Full set	1.519	0.747 (49.146%)	0.773 (50.853%)	9 (0.001%)	26012	5670	7529	2614
Helianthemum sanguineum (Lag.) Lag. ex Dunal	233	SAMN12818942	Subset / Full set	7.079	5.072 (71.652%)	2.007 (28.345%)	193 (0.003%)	469130	7243	16189	1813
Helianthemum sanguineum (Lag.) Lag. ex Dunal	295	SAMN12818943	Full set	2.824	2.137 (75.636%)	0.688 (24.356%)	231 (0.008%)	83157	6270	14563	1745
Helianthemum sauvagei Raynaud.	282	SAMN12818944	Subset / Full set	0.19	0.133 (69.691%)	0.058 (30.308%)	1 (0.001%)	7765	1768	236	135
Helianthemum scopulicolum L. Sáez, Alomar & Rosselló	363	SAMN12818945	Full set	3.329	2.236 (67.171%)	1.093 (32.828%)	33 (0.001%)	42833	10044	8333	1457

Helianthemum sessiliflorum Pers.	384	SAMN12818946	Full set	2.96	1.174 (39.669%)	1.786 (60.330%)	22 (0.001%)	47744	5770	7796	2527
Helianthemum sicanorum Brullo, Giusso & Sciandr.	297	SAMN12818947	Subset / Full set	2.038	1.332 (65.351%)	706 (34.646%)	49 (0.002%)	56653	7180	15076	3814
Helianthemum songaricum Zhao, Zhu & Cao	175	SAMN12818948	Subset / Full set	3.055	2.282 (74.681%)	774 (25.316%)	89 (0.003%)	127555	6565	9649	2324
Helianthemum sp nov 1*	405	SAMN12818949	Full set	2.728	1.500 (54.969%)	1.228 (45.027%)	86 (0.003%)	33749	9169	9210	1961
Helianthemum sp nov 2*	398	SAMN12818950	Full set	3.602	1.980 (54.984%)	1.621 (45.013%)	98 (0.003%)	32120	9674	11921	2528
Helianthemum sp nov 2*	432	SAMN12818951	Full set	1.399	0.800 (57.155%)	0.599 (42.843%)	25 (0.002%)	36092	8172	5078	1137
Helianthemum sp nov 3*	437	SAMN12818952	Full set	4.508	2.287 (50.726%)	2.221(49.272%)	71 (0.002%)	38025	10460	18163	3262
Helianthemum squamatum Pers.	57	SAMN12818953	Subset / Full set	4.703	3.392 (72.119%)	1.311 (27.878%)	129 (0.003%)	236017	5293	20225	1435
Helianthemum squamatum Pers.	46	SAMN12818954	Subset / Full set	3.034	2.071 (68.249%)	0.963 (31.749%)	47 (0.002%)	52529	4237	16811	1443
Helianthemum stipulatum C. Chr.	290	SAMN12818955	Subset / Full set	2.959	1.974 (66.720%)	0.985 (33.278%)	40 (0.001%)	77588	7598	19330	4387
Helianthemum syriacum (Jacq.) Dum.-Cours.	278 R1	SAMN12818956	Subset / Full set	1.612	1.122 (69.597%)	0.490 (30.402%)	10 (0.001%)	50419	4356	12316	1332
Helianthemum syriacum (Jacq.) Dum.-Cours.	278 R2	SAMN12818957	Subset / Full set	2.008	1.471 (73.242%)	0.537 (26.757%)	14 (0.001%)	59887	4434	13341	1458

Helianthemum syriacum (Jacq.) Dum.-Cours.	288	SAMN12818958	Subset / Full set	1.484	0.994 (66.988%)	0.490 (33.011%)	20 (0.001%)	57346	3913	7949	867
Helianthemum teneriffae Coss.	401	SAMN12818959	Full set	2.475	1.236 (49.929%)	1.239 (50.069%)	69 (0.003%)	42252	8306	6341	1450
Helianthemum tholiforme J. Ortega & B.Navarro	420	SAMN12818960	Full set	3.789	1.992 (52.583%)	1.796 (47.414%)	99 (0.003%)	40889	10059	13629	2798
Helianthemum thymiphyllum Svent.	375	SAMN12818961	Full set	9.847	7.065 (71.752%)	2.776 (28.192%)	5 (0.056%)	566793	8616	36055	4630
Helianthemum thymiphyllum Svent.	410	SAMN12818962	Full set	1.629	0.797 (48.965%)	0.831 (51.034%)	14 (0.001%)	34838	5641	8998	3010
Helianthemum tinetense M.Mayor & Fern.Benito	369	SAMN12818963	Full set	4.582	3.311 (72.270%)	1.270 (27.727%)	154 (0.003%)	280535	11006	10994	1458
Helianthemum ventosum Boiss.	387	SAMN12818964	Full set	3.67	1.626 (44.317%)	2.044 (55.682%)	28 (0.001%)	34908	6120	13841	3647
Helianthemum vesicarium Boiss.	388	SAMN12818965	Full set	2.241	1.004 (44.821%)	1.236 (55.178%)	26 (0.001%)	31666	6840	4179	618
Helianthemum violaceum Pers.	317	SAMN12818966	Subset / Full set	3.954	2.460 (62.227%)	1.493 (37.772%)	59 (0.001%)	88405	12255	14887	2431
Helianthemum virgatum (Desf.) Pers.	105	SAMN12818967	Subset / Full set	3.965	2.685 (67.718%)	1.280 (32.281%)	60 (0.002%)	75783	11941	11412	1860
Helianthemum viscarium Boiss. & Reut.	62	SAMN12818968	Subset / Full set	3.741	2.452 (65.561%)	1.288 (34.437%)	50 (0.001%)	81539	11772	12251	1690

Helianthemum viscidulum Boiss. subsp. ***raynaudii*** (Ortega Oliv., Romero García & C.Morales) G. López	70	SAMN12818969	Full set	9.847	7.065 (71.752%)	2.776 (28.192%)	5 (0.056%)	324873	5806	3742	332
Helianthemum viscidulum Boiss. subsp. ***viscidulum***	319	SAMN12818970	Full set	5.022	4.010 (79.856%)	1.011(20.134%)	468 (0.009%)	320980	7954	8140	1071
Tuberaria macrosepala (Coss.) Willk.	E008	SAMN12818971	Full set	6.505	4.619 (71.012%)	1.885 (28.985%)	153 (0.002%)	151570	1774	39225	393

* Non-described species (Santos-Guerra, 2014; Díaz-Bertrana com. pers)

Methods S1

DNA extraction, library preparation and Next-generation sequencing

Genotyping by sequencing (GBS) paired-end multiplexed libraries were constructed and sequencing by CNAG (Centro de Análisis Genómicos, Barcelona, Spain). Due to the limited previous experience within the genus *Helianthemum*, a small scale experiment was performed (pilot phase) using six samples to choose one of two different REs (restriction enzymes) to be used in the future large scale experiment (*Pst*I and *Ape*K1). Once the most suitable RE was selected based on the number of loci shared in all samples and SNPs recovered, the library preparation and sequencing of the rest of the samples were developed (large scale phase).

For the pilot phase, one sample with more than 7 µg of DNA and five samples above 2 µg were required (six samples in total). For the large scale phase, we obtained a minimum of 400 ng of DNA with a concentration between 50 and 200 ng/µl. The quality of the samples were evaluated by the absorbance ratios, being the optimal values between 1.8 and 2.0 and for the OD 260/280 and between 1.8 and 2.2 for the OD 260/230; and by integrity checked on agarose gel electrophoreses. Samples with small amount of smear or with slightly sticky DNA elutions were purified with the Genomic DNA Clean & Concentrator Kit (Zymo Research).

The library preparation and sequencing were developed following the protocol from Elshire et al. (2011) with improvements from Poland et al. (2012) and Sonah et al. (2013). In brief, the genomic DNA was again quantified by Quant-iT™ DNA High-Sensitivity Assay (Thermo Fisher Scientific) for the CNAG and 100ng/sample was used per reaction. In the pilot phase, each sample was digested in two parallel reactions with *ApeK*I (New England Biolabs) at 75ºC for 2hrs and *Pst*I (New England Biolabs) at 37ºC for 2hrs respectively. Adaptors compatible with Illumina sequencing were ligated using T4 DNA Ligase (New England Biolabs) to the 3 resp. 4 nucleotides overhangs left after the RE digestion. A titration to determine the adequate adaptor concentration was done in an independent experiment (described in Elshire et al. 2011 and Poland et al. 2012). Two different adaptors types combined together were used – the "indexed GBS adaptor" and the "common GBS adapter", each adaptor mix is specific to the RE used. After the AMPure XP beads (Agencourt, Beckman Coulter) purification the adaptor ligated reduced representation of the genome of all the 6 samples/RE were pooled and the pool was PCR amplified with 2x KAPA HiFi HS RM (Roche-Kapa Biosystems). The PCR primers were common to both RE specific GBS adaptors, one primer was the general primer and the second primer had integrated one of 8 Illumina barcodes. After the AMPure XP beads purification, the distribution of the fragment sizes within the pool of the sequencing libraries was assessed with an

Agilent 2100 Bioanalyzer DNA 7500 assay (Agilent). The six sequencing libraries corresponding to each RE protocol were sequenced on a MiSeq sequencer (Illumina) using a MiSeq v2 flow cell (Illumina) in paired end mode and with a read length of 2x150bp. After analysis the protocol using the *ApeK*I RE was selected for large scale reduced representation genome sequencing. The large scale GBS library preparation was processed within a 96 well plate following the *ApeK*I RE protocol as described above, only the pool of the adaptor ligated libraries consisted of up to 12 individuals and after the PCR amplification all 96 individuals of each plate were joined into the final sequencing pool. After the final quality control on the Bioanalyzer DNA 7500 chip the library pool was sequenced on an Illumina HiSeq2000 (Illumina) in paired-end mode with a read length of 2x125bp using the TruSeq SBS Kit v4, following manufacturer's protocol.

Methods S2

Description of the bioinformatic parameters explored in this study

- Data type (Merged vs. unmerged)

The result of sequencing paired-end GBS libraries is the forward and reverse strands of each target DNA fragment. If a given overlap exists, the corresponding paired-end reads can be merged into a fragment, diving rise to merged data. If no overlap exists, forward and reverse reads are treated independently and constituted unmerged data. Merging paired-end reads is the first processing step in a plethora of sequence analysis pipelines (Zhang et al., 2014).

- Clustering thresholds

This parameter represents the level of sequence similarity (coding as percentage of matches) at which two sequences are identified as being homologous, and thus cluster together to form a locus in the assembly (Eaton, 2014).

- Minimum sample coverage

This parameter represents the minimum number of reads per position that must present a sequence to form a locus in the assembly. Determine the reliability of each nucleotide position in the consensus sequence (Eaton, 2014).

- Statistical Base Calling

It is the standard way to do the consensus base calling throughout a minimum sample coverage value. To generate consensus sequences under the Statistical Base Calling method, pyRAD calculate the error rate (ϵ) and heterozygosity (π) are jointly estimated from the observed base counts across all sites in all clusters, by applying the maximum-likelihood equation of (Lynch, 2008). The mean ϵ is then used to assign consensus diploid genotypes for each site in each cluster by calculating the binomial probability the site is homozygous (aa or bb) versus heterozygous (ab) given the relative frequencies of observed bases at the site and ϵ (Li et al., 2008). If a base cannot be assigned with ≥ 95% probability it is replaced by N in the consensus sequence. Heterozygotic variation is recorded using appropriate ambiguity codes. The end result of this step is a set of consensus sequences of putative RAD loci for each barcoded DNA sample (Eaton, 2014).

- Majority-rule Base Calling

It is another way to do the consensus base calling when the aimed data set has very low coverage such that many clusters are excluded due to low sequencing depth. Majority-rule Base Calling can be an effective way to increase the amount of usable information in the data set. In this case, the consensus nucleotide is the one appearing more often than the rest (Eaton, 2014).

In order to disable this option (and developing a standard Statistical Base Calling) this is set to the same value as minimum sample coverage, such that only statistical base calls are made. If it is set lower, then sites with coverage >= minimum sample coverage will make majority rule calls. Care should be taken since majority rule consensus base calls will underestimate heterozygosity (Eaton, 2014).

- Minimum taxon coverage

The minimum number of samples that must have data at a given locus for it to be retained in the final data set. If inputting a number equal to the number of samples in the data set then it will return only loci that have data shared across all samples. By entering a lower value, it will return a more sparse matrix, including any loci for which at least the minimum taxon coverage established contain data (the rest is filling up as missing data) (Eaton, 2014).

References Methods S1-S2

Eaton, D. A. R. (2014). PyRAD: assembly of de novo RADseq loci for phylogenetic analyses. *Bioinformatics* 30: 1844–1849. doi: 10.1093/bioinformatics/btu121

Elshire, R. J., Glaubitz, J. C., Sun, Q., Poland, J. A., Kawamoto, K., Buckler, E. S. and Mitchell, S. E. (2011). A robust, simple Genotyping-by-Sequencing (GBS) approach for high diversity species. *PLOS ONE* 6: e19379. doi: 10.1371/journal.pone.0019379

Poland, J. A., Brown, P. J., Sorrells, M. E. and Jannink, J-L. (2012). Development of high-density genetic maps for barley and wheat using a novel two-enzyme genotyping-by-sequencing approach. *PLOS ONE* 7: e32253. doi: 10.1371/journal.pone.0032253

Sonah, H., Bastien, M., Iquira, E., Tardivel, A., Légaré, G., Boyle, B., et al. (2013) An Improved Genotyping by Sequencing (GBS) Approach Offering Increased Versatility and Efficiency of SNP Discovery and Genotyping. PLoS ONE 8(1): e54603. doi: 10.1371/journal.pone.0054603

Zhang, J, Kobert, K., Flouri, T., Stamatakis, A. (2014). PEAR: a fast and accurate Illumina Paired-End reAd mergeR. *Bioinformatics* 30: 614–620. doi: 10.1093/bioinformatics/btt593
